# Five Regions of the Pea Genome Co-Control Partial Resistance to *D. pinodes*, Tolerance to Frost, and Some Architectural or Phenological Traits

**DOI:** 10.3390/genes14071399

**Published:** 2023-07-04

**Authors:** Gilles Boutet, Clément Lavaud, Angélique Lesné, Henri Miteul, Marie-Laure Pilet-Nayel, Didier Andrivon, Isabelle Lejeune-Hénaut, Alain Baranger

**Affiliations:** 1 IGEPP, INRAE, Institut Agro, Université de Rennes, 35653 Le Rheu, France; 2 BioEcoAgro Joint Research Unit, INRAE, Université de Lille, Université de Liège, Université de Picardie Jules Verne, 80200 Estrées-Mons, France

**Keywords:** frost tolerance, *Ascochyta* blight, quantitative resistance, multi-stress, flowering, plant architecture, *Pisum sativum*, QTL, genetic mapping, colocalization, RILs

## Abstract

Evidence for reciprocal links between plant responses to biotic or abiotic stresses and architectural and developmental traits has been raised using approaches based on epidemiology, physiology, or genetics. Winter pea has been selected for years for many agronomic traits contributing to yield, taking into account architectural or phenological traits such as height or flowering date. It remains nevertheless particularly susceptible to biotic and abiotic stresses, among which *Didymella pinodes* and frost are leading examples. The purpose of this study was to identify and resize QTL localizations that control partial resistance to *D. pinodes*, tolerance to frost, and architectural or phenological traits on pea dense genetic maps, considering how QTL colocalizations may impact future winter pea breeding. QTL analysis revealed five metaQTLs distributed over three linkage groups contributing to both *D. pinodes* disease severity and frost tolerance. At these loci, the haplotypes of alleles increasing both partial resistance to *D. pinodes* and frost tolerance also delayed the flowering date, increased the number of branches, and/or decreased the stipule length. These results question both the underlying mechanisms of the joint control of biotic stress resistance, abiotic stress tolerance, and plant architecture and phenology and the methods of marker-assisted selection optimizing stress control and productivity in winter pea breeding.

## 1. Introduction

Controlling the effects of diseases, pests and climate variations on crop growth and yield will gain from a better understanding of the relationships between architectural and developmental traits on one hand and plant responses to biotic or abiotic stresses on the other hand. In the literature, reciprocal links between these different groups of traits have already been analyzed using epidemiology, physiology, or genetics.

Epidemiological processes and plant responses to diseases indeed interfere with plant or canopy architectural and developmental traits, which modify the receptivity and susceptibility of organs to pathogens/pests, the dispersal of propagules or individuals, and microclimate gradients within canopies. Some such processes include plant aging in relation to light interception; mechanical barriers to dispersion; and the establishment of optimal temperatures, humidity, and leaf wetness duration favoring pathogen development [1,2]. Dense and compact foliage structures enhance crop susceptibility to many foliar diseases, whereas well-ventilated, porous stands usually reduce disease severity. Such impacts can be either direct (higher density of susceptible tissue in dense canopies) or mediated by altered microclimate gradients within canopies [3]. Conversely, architectural traits can be modified by the development of epidemics as evidenced for the root architecture of tomato [4,5], pea [6], or *Medicago truncatula* [7]. Similar effects are noted in aerial diseases, leading to partial or complete defoliation of the host. Reciprocal links between plant architecture and responses to abiotic stresses have also been observed, although plant and canopy architectural traits are more likely to modify abiotic stresses in their impact than in their intensity. Some plant species are able to adapt their architecture in response to environmental conditions, such as autumnal short days and low temperatures, by developing a rosette architecture, characterized by small aerial organs and prostrate growth, which allows them to tolerate subsequent freezing periods [8,9,10]. Conversely, abiotic stresses are known to affect root and shoot morphology [11] and plant metabolism and growth [12,13].

Examining the physiological and metabolic mechanisms involved in the responses to either biotic or abiotic stresses allows for evidence of convergent signaling pathways to be determined, which are likely to interfere in multiple stress responses (see the review by Atkinson and Urwin [14]). Common regulatory pathways have especially been pointed out in stress signal transduction, such as the mitogen-activated protein kinase cascades, the production of reactive oxygen species, or the hormone signaling pathways [14,15]. The co-regulation of downstream response genes at the post-transcriptional level has also been mentioned [14,16]. Synergistic or antagonistic interactions of co-regulators for combined stress responses cannot, however, be generalized, and plants are more likely to face conflicting demands when exposed to multiple stresses, resulting in metabolic trade-offs [15]. Moreover, the inhibition of plant growth under stress also reflects additional compromises between growth and response to stresses [17].

Mendelian genetics approaches have revealed overlapping genomic regions that control both plant architecture or development and either biotic [18] or abiotic [19] stress response. Such colocalizations support the hypothesis that at least some loci responsible for quantitative disease resistance are based on genes controlling for plant architecture or development [20]. The pea (*Pisum sativum* L.) is a suitable model for investigating the genetic relationship between responses to abiotic or biotic stresses and architectural or developmental traits, as it exhibits a great architectural and developmental polymorphism in relation to its indeterminate growth, as well as a large genetic variability in its responses to many biotic and abiotic stresses. Exploring the genetic structure of both stress response and plant architecture is of particular interest for the winter pea crop, which suffers from two major stresses in temperate countries, i.e., *Ascochyta* blight, caused by *D. pinodes* [21], and frost [22], both of which hinder improvements in and the stability of seed yield. Overlapping quantitative trait loci (QTLs) have already been detected between stress responses and traits such as plant height, number of branches, date of flowering, aerial biomass, or stipule area [23,24,25,26,27,28,29,30], but a comprehensive assessment of colocalizations in diverse genetic backgrounds and mapping populations is still lacking.

Here, we gathered already published and new phenotypic data on three recombinant inbred line (RIL) populations, as well as new, improved genotyping to build a high-density consensus map in order to revisit the likely convergence in the genetic determinism of *D. pinodes* resistance, frost tolerance, and some architectural and developmental traits. Our aims were therefore (i) to identify the new, to confirm the old, and to resize all QTL localizations that control partial resistance to *D. pinodes*, tolerance to frost, and architectural or phenological traits on pea dense genetic maps; (ii) to identify colocalizations between QTLs for these traits, within and between RIL populations; and (iii) to discuss the impact of these colocalizations for winter pea breeding.

## 2. Materials and Methods

The genetic approach relies on three biparental RIL populations segregating for *D. pinodes* partial resistance, frost tolerance, and architectural traits. Two populations had previously been characterized for either frost tolerance and flowering in the field [30] and in controlled conditions [31], for resistance to a single *D. pinodes* isolate under controlled conditions, or for resistance to a mixture of isolates in the field [24]. In order to identify consistent QTLs for *D. pinodes* resistance likely to be independent of canopy architecture effects in the field, *D. pinodes* resistance assessment was enlarged, in this study, both to plantlets and to detached stipules under controlled conditions on all three RIL populations.

### 2.1. Fungal and Plant Material

Based on initial pathogenicity tests with six single *D. pinodes* monosporic strains on a set of 11 pea genotypes (Supplementary File S1), three *D. pinodes* monosporic isolates with high (Mp 91 31 12), low (Mp 97 WVF 421), or intermediate (Mp 94 01 3) aggressiveness on pea were chosen for further phenotyping. 

Parental lines for the production of three recombinant inbred line (RIL) populations for further genetic analysis were chosen within the initial set of 11 pea genotypes according to known polymorphism for architectural and developmental traits (including foliage types, alleles at major node elongation (*le*), or photoperiod responsive (*hr*) genes) (Supplementary File S1), partial resistance to *D. pinodes* [32,33], and frost tolerance [30,31]. All three populations, from the crosses Champagne x Terese (below called ChxTe; 151 F8 RILs, described in [30]), JI296 x DP (below called JIxDP; 120 F6 RILs, described in [24]), and JI296 x FP (below called JIxFP; 142 F7 RILs obtained from F2-derived lines produced under a greenhouse by single seed descent at INRAE Le Rheu, France) were therefore expected to segregate for several plant architectural traits, partial resistance to *D. pinodes*, and tolerance to frost.

### 2.2. Partial Resistance to D. pinodes Assessed on Whole Plantlets and Detached Stipules under Controlled Conditions

*D. pinodes* severity was assessed in climatic chambers both on 5-week-old whole plantlets and on detached stipules from plantlets. Plant growth, inoculation, incubation, and screening procedures are described in Onfroy et al. [34] and Onfroy et al. [32].

The test on whole plantlets was carried out following a completely randomized design of two seeded pots in 6 blocks (12 seeds per line) for most of the trials, with the exception of 9 blocks (18 seeds per line) for the screening of ChxTe RIL population with the Mp 94 01 3 strain. Plants grown in trays in a growth chamber kept for four weeks at 12 °C day/10 °C night with a 12 h photoperiod were further inoculated by spraying a suspension of spores from each of the monosporic strains at a concentration of 10^5^ spores/mL. Inoculated plantlets were then covered with plastic lids and temperature raised to 20 °C day/18 °C night with a 12 h photoperiod. Disease Severity (DS) was assessed twice a week on each of the first three internodes on stipules and on stems separately, during three consecutive weeks, using a 0 to 5 disease scale described in Onfroy et al. [34]. Areas Under Disease severity Progress Curves (AUDPC) from the inoculation to the end of the experiment were calculated with mean disease index per plant and per organ, as described by Shaner and Finney [35], separately on stipules (DS_STIP) and on stems (DS_STEM).

Growing plant conditions and experimental design prior to detached stipule tests were similar to plantlet tests until inoculation, except that a reduced set of 100 lines within each RIL population was randomly chosen. Four weeks after sowing, the two stipules of the third internode of each plant were cut and transferred to compartmented Petri dishes filled out with tap water. A drop of 10 μL suspension of spores, at a 2 × 10^5^ spores/mL concentration, was deposited on each stipule. Petri dishes containing inoculated stipules were then brought together in trays and covered with plastic lids to limit drop evaporation. After 18 h incubation in darkness at 20 °C, the growth chamber temperatures were set to 20 °C day/18 °C night with a 12 h photoperiod. Symptom development was assessed each day, from the 2nd to the 8th or 10th day following inoculation, using a 0 to 3 semi-quantitative scale estimating flecks development (number and coalescence) at days 2 and 3 after inoculation, and then daily mean values for lesion diameters (from the 4th day after inoculation). Both means of Flecks Coalescence (FC) per plant at 2 and 3 days after inoculation (further named FC_2D and FC_3D, respectively) as well as AUDPC of necrosis lesion expansion (further named DS_LE) from daily mean values of lesions diameter were calculated and used for QTL mapping.

For easier analysis and interpretation, all disease severity variables are suffixed with S1, S2, or S3 depending on the monosporic strain used for inoculation, i.e., Mp 91 31 12 (S1), Mp 94 01 3 (S2), and Mp 97 WVF 421 (S3), respectively.

### 2.3. Architectural Traits Assessments on Whole Plantlets or Detached Stipules under Controlled Conditions

Plant growing conditions and experimental designs for architectural traits assessments were similar to those used in the whole plantlets and detached stipules tests. Assessments on whole plantlets and on detached stipules were run on non-inoculated separate sets. After a 4-week growing period, height (from the soil substrate to the last deployed internode; Ht), internode number (NbNo), and number of primary branches (NbBr) were assessed on each plantlet and length of stipules (StLe) on both stipules of the three first internodes for ChxTe and JIxDP and both stipules of the third internode for JIxFP.

### 2.4. Frost Tolerance

Least square means (LSMeans) for each frost tolerance trait under field and controlled conditions from the ChxTe RIL population published, respectively, by Lejeune-Hénaut et al. [30] and Dumont et al. [31] were used for QTL analyses. The date of the beginning of flowering (DBF) previously assessed on this population (Lejeune-Hénaut et al. [30]) was also included.

### 2.5. Statistical Analyses for D. pinodes Disease Severity, Architectural Traits, and Frost Tolerance

The generalized linear model (PROC GLM) from the SAS software (SAS Institute Inc. 2000) was used to analyze phenotypic data through ANOVA, using a mixed model including genotype and block factors and their interaction. For each trait, the normality of residuals was checked with the Shapiro and Wilk’s test (PROC UNIVARIATE, *p* > 0.05), and the homogeneity of variances was checked using the Bartlett’s test (HOVTEST, *p* > 0.05). ANOVA results allowed the broad sense heritability of the different traits to be determined as h^2^ = σ^2^g/[σ^2^g + (σ^2^e/n)], with the genetic variance σ^2^g, the number of replicates per genotype n, and the residual variance σ^2^e. RILs’ LSMeans were calculated for each trait from ANOVA results and used for QTL analyses.

Pearson’s correlation coefficients were calculated between each trait within each population using the R function cor from the R software [36], and heatmaps of Pearson’s correlation coefficients were drawn and hierarchically clustered using the R function corrplot.

### 2.6. SNP KASP™ Genotyping Assays

Recently developed SNPs [37,38,39] were screened based on their genetic positions on the available maps in order to densify both genomic regions likely to contain QTLs of interest (2.5 SNPs/cM) and the genetic background (1 SNP/2 cM). The PsCam SNP markers [38] correspond to genes available via the pea Gene Atlas [40] and are anchored on the pea reference genome [41]. Their physical position is currently available via the public genome browser (https://urgi.versailles.inra.fr/jbrowse/gmod_jbrowse, accessed on 29 June 2023). The Ps1 SNP markers were developed by sequencing the complete whole genomic DNA (therefore including the non-coding regions) of four pea lines including Champagne and Terese, the parental lines of the ChxTe RIL population [37]. The resulting 1903 SNPs allowed the design of 1536-well plate KASP™ [42] assays. Genotyping was performed by LGC Genomics service lab, UK (http://www.lgcgenomics.com, accessed on 29 June 2023), as described in Boutet et al. [37].

### 2.7. Construction of Three High-Resolution Individual RIL Maps

To build individual maps for each of the 3 RIL populations, we added KASP™ genotyped SNPs to major genotyping data used for previous constructions of ChxTe [30,39,43], JIxDP [24,39,43], and JIxFP [39,43,44] individual and consensus genetic maps. The final ChxTe, JIxDP, and JIxFP genotyping data matrices comprised a total of 1920, 1544, and 1346 markers, including 1419, 1149, and 1066 new KASP™-genotyped SNPs markers, on 151, 120, and 142 RILs, respectively.

The 1:1 allelic segregation ratio for each marker within each RIL population was checked using a Chi-square test (*p* > 0.01 and *p* > 0.001). Genetic linkage analyses were performed using the “group” command of CAR_H_^T^AGENE software [45] with a minimum LOD score threshold of 7.0 and a recombination frequency < 0.3. The order of the markers was refined using the “annealing 100 100 0.1 0.9” command of CAR_H_^T^AGENE. The Haldane function was used to calculate cM distances between markers [46], and MapChart 2.2 was used to draw the maps [47].

### 2.8. QTL Analyses

R software [36] with the Package ‘qtl’ [48] was used to perform QTL analysis on each individual map, as a first approach with the composite interval mapping method (CIM), using a 5 cM window size. LOD thresholds were determined individually for each trait in each population and after 1000 permutations in order to identify significant one-trait QTLs corresponding to a 5% error risk of false positive all over the genome [49]. One to ten cofactors were tested for each trait in order to choose the best cofactors to use for each CIM analysis.

The fitqtl multiple-QTL model analysis was then used to validate each QTL showing an LOD score exceeding the threshold obtained with the CIM method. The addqtl function was subsequently used to scan for any additional QTL in the multiple-QTL model. The 1-LOD confidence intervals were finally defined for each validated QTL.

A composite map including the three individual maps with all their respective markers and QTLs was constructed using the BioMercator software [50]. Four different metaQTL analyses were performed in BioMercator with the Gerber and Goffinet meta-analysis model [51], choosing the smallest AIC value for QTL integration: a first analysis with all *D. pinodes* resistance one-trait QTLs generated Dp.x.x metaQTLs; a second analysis with all field and controlled conditions frost tolerance one-trait QTLs generated FR.x.x metaQTLs; a third analysis with all architectural and phenological one-trait QTLs (Ht, StLe, NbNo, NbBr, dflo/DBF) generated A.x.x metaQTLs; and finally, a joint analysis, carried out with all one-trait QTLs, generated MDA (standing for Meta Disease Architecture) or MDAF (standing for Meta Disease Architecture Frost) metaQTLs.

## 3. Results

### 3.1. High-Density Individual Genetic Maps

The three resulting individual genetic maps (below called CT-map, JD-map, and JF-map), covered 903, 693, and 901 cM, respectively, on seven (CT-map, JF-map) or eight (JD-map) linkage groups and had a marker density from 1.5 to 2.2 markers/cM. Positions of the mapped markers were generally collinear between the three maps (Supplementary Figure S1). However, the JD-map presented two particular features. First, 97 markers were grouped on only two closely linked (0.1 cM genetic distance) bins at the 39.3 and 39.4 cM positions on the LGVI of the JD-map, whereas they were ordered on 24 and 11 bins covering 24.5 cM and 28.6 cM on the CT- and JF-maps, respectively, revealing a likely chromosomal rearrangement of this region in the DP genome. Second, LGVII was subdivided into two subgroups on the JD-map: LGVII.1, collinear to the apical part of LGVII on the CT- and JF-maps, and LGVII.2, collinear to the distal part of LGVII on the CT- and JF-maps. Surprisingly there were no common markers between the CT- and JF-maps in this distal part of LGVII, but each had common markers with the LGVII.2 subgroup of the JD-map. Positions of KASP^TM^ SNPs on these three new maps were generally consistent with their previously published positions [37,38]. For each individual new map, mapping thousands of markers with the CAR_H_^T^AGENE annealing method allowed a highly accurate QTL detection for the large range of phenotyped traits of interest.

The high-density composite pea genetic map (Supplementary Figure S1), constructed from the three individual maps (CT-map, JD-map, and JF-map) comprised 2744 markers (including 1812 newly genotyped KASP^TM^ SNP markers) covering 915 cM, which is an intermediate size between the previously published pea composite (795 cM [38]) and consensus (1255 cM [39]) map sizes. The composite map showed 79 gaps larger than 2 cM and only 5 gaps larger than 5 cM between two contiguous markers (Table 1). Marker density ranged from 2.6 to 3.7 markers/cM (1.7 to 2.6 KASP^TM^ SNPs/cM) depending on linkage groups (Table 1). The positions of the mapped markers on the composite map were generally consistent with their positions on the three individual maps and with their previously published positions [30,37,38,39].

### 3.2. Phenotyping D. pinodes Severity and Architectural Traits Data on RIL Populations

The distributions of ANOVA residuals for all variables did not significantly deviate from normality, and homoscedasticity was validated for most variables (with the exception of FC_2D_S1FC_2D_S2, DS_STE_S2, and NbNo for both ChxTe and JIxFP; of DS_LE_S2 for ChxTe; and of NbBr for JIxFP). Genotype as well as genotype x block effects were significant for all the observed variables. However, in all cases, genotype x block effects could be considered as negligible compared to genotype individual effects. Broad sense heritability values ranged from 0.22 to 0.97, with an overall mean of 0.67 (Supplementary Table S1). They were high for all disease variables, but lower in JixFP than in the two other RIL populations. They were highest for architectural traits such as Ht, NbBr, and StLe. Compared to other traits, NbNo showed a much lower broad sense heritability value, probably related to a dominating effect of environmental conditions on this trait in all three populations (Supplementary Table S1).

### 3.3. Correlations

#### 3.3.1. Correlations between *D. pinodes* Disease Severity Traits

**Figure 1:**
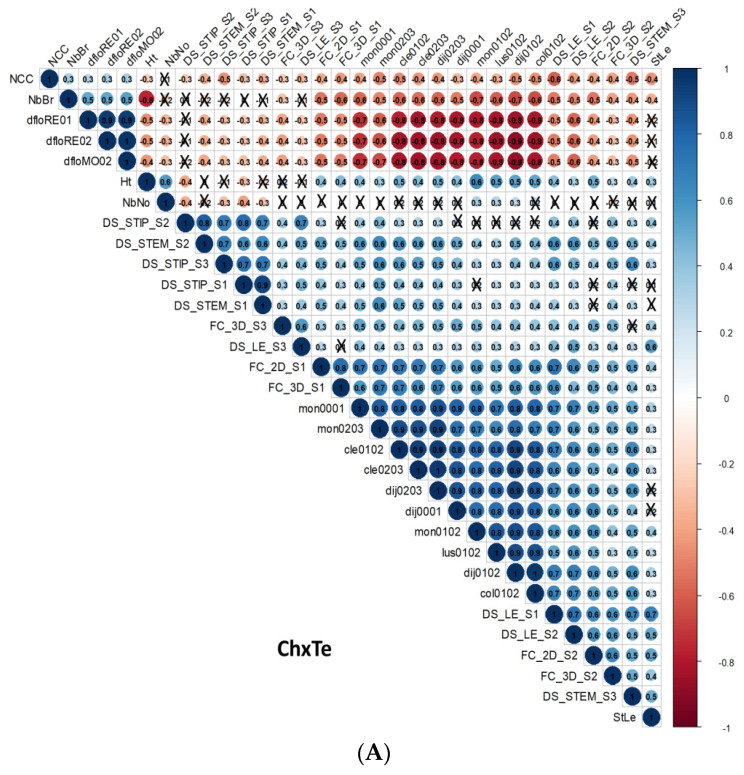

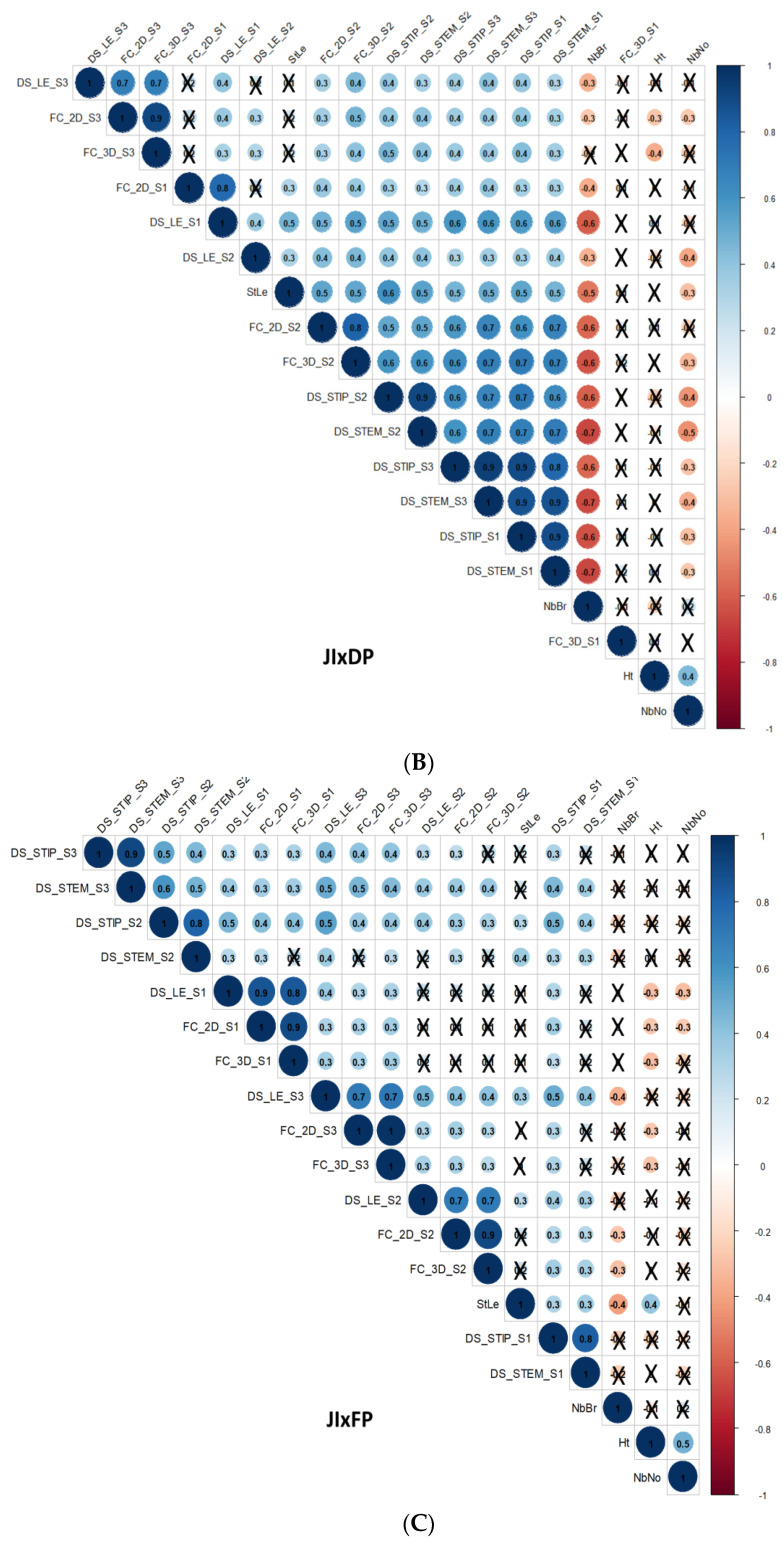
Heatmap of Pearson’s correlation coefficients between disease severity traits, between frost tolerance traits, between architectural traits, and between disease severity, frost tolerance, and architectural variables in the RIL populations (**A**) ChxTe (FC_2D_S3 missing), (**B**) JIxDP, and (**C**) JIxFP. Frost tolerance traits and flowering date are available only for ChxTe. Blue and red colors indicate positive and negative correlations, respectively. Crosses mark non-significant correlations (*p* > 0.01). Trait abbreviations are as mentioned in Section 2.2: DS: Disease Severity evaluated as the Area Under the Disease severity Progress Curve, assessed on whole plantlet stipules (STIP) or stems (STEM) or on detached stipules (LE); FC: flecks coalescence assessed on detached stipules two (2D) or three (3D) days after inoculation; Sx: *D. pinodes* strain of high (S1), intermediate (S2), or low (S3) aggressiveness on pea; Frost tolerance in controlled conditions (NCC) or in the field in Clermont-Ferrand (cle), Colmar (col), Dijon (dij), Lusignan (lus), Mons (mon), followed by year of evaluation; StLe: stipule length; Nbbr: number of branches; NbNo: number of nodes; Ht: plant height; dflo: date of beginning of flowering in Mons (MO) or Rennes (RE), followed by year of evaluation (stands for DBF [30]).

Significant positive correlation coefficients between most disease severity variables were observed in both plantlets and detached stipules assessments within each of the three RIL populations (Figure 1), showing the homogeneity of the disease severity evaluation, whatever the organ or the strain considered. A few exceptions were recorded, revealing strain specificity regarding some variables within one population or the other.

#### 3.3.2. Correlations between Architectural and Phenological Traits

Positive correlations between plant height and number of nodes and negative correlations between stipule size and number of branches were commonly observed within all three populations. For other traits, significant correlations were specific to one or two populations among three. The flowering date, assessed only in the ChxTe population, was positively correlated to the number of branches and negatively correlated to the plant height and the number of nodes.

#### 3.3.3. Correlations between *D. pinodes* Disease Severity Traits and Phenological or Architectural Traits

Disease severity was generally negatively correlated to plantlet height whether assessed on plantlets or detached stipules, significant values being sometimes specific to one strain and/or to one population. Conversely, disease severity was mostly positively correlated to stipule length within populations and strains. The coefficients were highest in the ChxTe population, suggesting a stronger link between disease severity and stipule size in this population. Finally, disease severity was generally negatively correlated to the number of branches and to the number of nodes, sometimes with a specificity regarding the strain.

#### 3.3.4. Correlations between Environments, Assessment Methods for Frost Tolerance, Architectural and Phenological Traits, and Disease Severity Assessments within the ChxTe Population

Correlation coefficients between environments (sites and years) for field frost tolerance variables within the ChxTe population were all positive, showing a high consistency of frost tolerance assessment across environments. Correlation coefficients between field [30] and controlled conditions (NCC) [31] were negative due to inverted scales (Lejeune-Hénaut, personal communication). Field frost tolerance variables were negatively correlated to the flowering dates and to the number of branches and positively correlated to plant height and to the number of nodes. Finally, correlation coefficients between frost tolerance variables in the field and disease variables, assessed both on whole plantlets and on detached stipules, were all positive.

### 3.4. QTLs Controlling D. pinodes Disease Severity, Frost Tolerance, and Some Architectural and Phenological Traits

#### 3.4.1. QTL Detection on Each Individual Map

One hundred and fifty-three one-trait QTLs were detected on the CT-, JD-, and JF-maps (Supplementary Figure S2). More than 80% of these clustered to regions covered less than 10% of each map size. The most significant regions suggested colocalizations between QTLs controlling *D. pinodes* severity and frost tolerance and included QTLs and/or major genes for architectural or phenological traits. Most of these QTLs were consistent between three or two maps, and only a few (on LGVII) were specific to one map (JD-) or the other (JF-).

#### 3.4.2. QTL Projection on the Composite Map and metaQTL Analysis by Trait

All 153 one-trait QTLs were subsequently projected onto the composite map, and three metaQTL analyses were performed separately for *D. pinodes* severity, architectural traits, and frost tolerance (Supplementary Table S2).

The projection on the composite map (Supplementary Figure S3) and the metaQTL analysis allocated the 80 QTLs controlling *D. pinodes* severity to 22 Dp metaQTLs (Supplementary Table S2). The allele contributing to lower *D. pinodes* severity was brought by the resistant parent (Champagne, DP or FP) in 17 of the 22 metaQTLs and by the susceptible parent (JI296 for Dp.1.3, Dp.3.5, Dp.3.6 and Terese for Dp.3.2, Dp.6.4) in the remaining five. Seventeen Dp metaQTLs among the twenty-two were specific to a single population. Only 10 Dp metaQTLs were detected only once (for a single trait and a single strain). Six consistent Dp metaQTLs (Dp.5.1, Dp.5.2/Dp.5.3, Dp.6.1/Dp.6.2, Dp.3.3), standing for 68% of the 80 QTLs, were detected in at least two of the three populations. They were neither specific to an organ, a phenotyping method, nor a strain. Additionally, two Dp metaQTLs (Dp.3.2 and Dp.6.3, standing for 6% of the 80 QTLs detected) were population-specific but neither organ nor strain specific, and four metaQTLs (Dp.3.1, Dp.3.9, Dp.7.1, and Dp7.3, standing for 13% of the 80 QTLs detected) were both specific to a single population and to a given organ.

The projection on the composite map (Supplementary Figure S3) and the metaQTL analysis allocated the 39 QTLs controlling phenological and architectural traits to 17 A metaQTLs (Supplementary Table S2). Eleven A metaQTLs among the seventeen were specific to one of the populations. The six remaining consistent A metaQTLs, standing for 67% of the 39 QTLs detected, were common to at least two of the three populations.

The projection on the composite map (Supplementary Figure S3) and the metaQTL analysis allocated the 34 QTLs controlling frost tolerance in ChxTe to 7 FR metaQTLs (Supplementary Table S2) in which frost tolerance was contributed by alleles from the tolerant parent Champagne, with the exception of FR.3.1, for which frost tolerance was contributed by alleles from the susceptible parent Terese.

#### 3.4.3. Joint metaQTL Analysis

Eighty-two percent of the 153 one-trait QTLs detected on the composite map (Supplementary Table S2) clustered to only 10 small-size regions (0.4 to 3.2 cM confidence intervals), when processed together with the joint meta-analysis. These regions displayed colocations either between QTLs controlling disease severity and QTLs controlling architectural traits (below called MDA QTLs, standing for Meta Disease Architecture QTLs) or between QTLs controlling disease severity, QTLs controlling architectural traits, and QTLs controlling frost tolerance (below called MDAF QTLs, standing for Meta Disease Architecture Frost QTLs) (Table 2). The remaining 18% of the 153 QTLs were metaQTLs or one-trait QTLs specifically controlling either disease severity or architectural traits (Supplementary Table S2).

**Figure 2:**
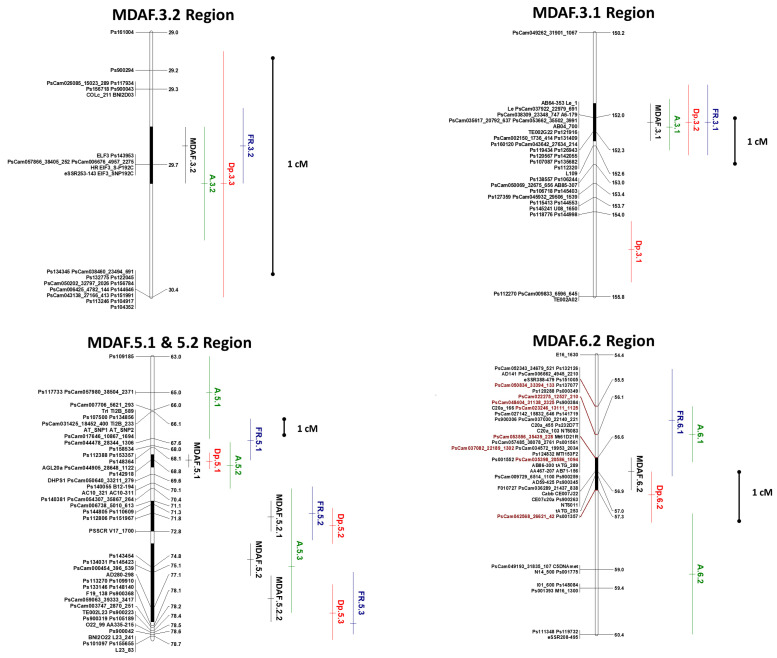
Position on the composite map of the MDAF regions and of corresponding disease resistance, frost tolerance, and architecture metaQTLs. MetaQTLs are represented by vertical bars on the right of the linkage groups, including a horizontal bar indicating the peak position. MDAF metaQTLs are in black, Dp metaQTLs in red, frost metaQTLs in blue, and architecture metaQTLs in green. Confidence intervals of MDAF metaQTLs are also represented by black bars inside the linkage group’s bar. Markers in brown correspond to frost tolerance GWAS markers presented in Beji et al., 2020 [52].

Five regions of the pea genome concomitantly controlled a large part of the variation in resistance to *D. pinodes*, frost tolerance, and architectural traits. MDAF.3.1, MDAF.3.2, MDAF.5.1, MDAF.5.2, and MDAF.6.2, gathered more than 70% of the 153 one-trait QTLs initially detected (Figure 2). The three trait categories, i.e., *D. pinodes* disease severity, frost tolerance, and architecture, showed colocalizing one-trait QTLs in two or three populations. At these five loci, the favorable allele for both stress responses was consistently the same (Table 2, Supplementary Table S2), i.e., an allele increasing *D. pinodes* resistance also increases frost tolerance. Moreover, these five loci also support constant relationships with some architectural traits (Table 3, Supplementary Table S2). Thus, favorable alleles for *D. pinodes* resistance and frost tolerance were also responsible for a delayed flowering date (MDAF.3.1, MDAF.3.2, and MDAF.6.2), a higher number of basal branches (MDAF.3.1, MDAF.3.2, MDAF.5.2, and MDAF.6.2), and smaller stipules (MDAF.3.1, MDAF.5.1, and MDAF.6.2). Finally, at MDAF.3.1, the favorable stress response alleles also contributed to reduced plant height and number of nodes.

At MDAF.5.2, the projection of the one-trait QTL CT_68_NbBr, represented by the corresponding metaQTL A.5.3 (Figure 2), resulted in two statistically equivalent options: (i) a single MDAF region (MDAF.5.2) or (ii) two MDAF regions (MDAF.5.2.1 and MDAF.5.2.2). The CT_68_NbBr QTL could have artificially linked these two regions due to its in-between position and its relatively large confidence interval (7 cM). Disregarding this one-trait QTL, the meta-analysis yielded two distinct metaQTLs, both controlling frost tolerance and disease resistance.

Major loci involved in phenological/architectural traits were mapped at the vicinity of these MDAF QTLs peaks. The major locus *Hr* (High response to photoperiod) was mapped on LGIII at 29.7 cM on the composite map, within the 0.3 cM MDAF.3.2 confidence interval. It was the peak position of each of the FR.3.1.2, Dp.3.3, and A.3.2 metaQTLs (Figure 2). Furthermore, the major locus *Le* was mapped on LGIII at the position 152.0 cM, within the 0.9 cM MDAF.3.1 confidence interval. It was the peak position for each of the FR.3.1, Dp.3.2, and A.3.1 metaQTLs (Figure 2). The flowering gene AGAMOUS-LIKE 20 (SOC1/AGL20) was mapped on LGV close to the peak positions of each of the FR.5.1, Dp.5.1, and A.5.2 metaQTLs (Figure 2).

Five additional MDA or MDAF regions contributed to the genetic determinism of the studied traits. Four MDA metaQTLs, i.e., MDA.1.1, MDA.6.1, MDA.7.1, and MDA.7.2, and one MDAF metaQTL, i.e., MDAF.6.3, gathered 11% of the 153 one-trait QTLs within small size confidence intervals. They mainly completed the view of the common genetic structure of *D. pinodes* resistance and architecture. The alleles contributing to *D. pinodes* resistance, frost tolerance, and architecture at these five additional loci were not repeatedly associated (as was the case for the MDAF positions presented in the previous section).

The four MDA metaQTLs were specific to a single population. The position of MDA.6.1 remains questionable, since its peak marker, i.e., PsCam035356_20546_778, was polymorphic only in the JD population, within the region being suspected to be rearranged between JD and the two other populations (see Section 3.1). Its projection on the composite map may therefore be approximate. This is also suggested by the physical position on the reference genome of the PsCam035356_20546_778 marker, polymorphic only in JIxDP at the peak of MDA.6.1, between the physical positions of the PsCam markers at the peaks of MDAF.6.2 and MDAF.6.3, respectively (Supplementary Table S3). The size of one-trait-QTLs confidence intervals could also question the projection within a metaQTL or another. This was the case for JD_17_NbBr, which exhibited a 32 cM confidence interval and was projected within MDAF.6.3, while the two other one-trait QTLs constituting the A.6.2 metaQTL were projected within MDAF.6.2 (Table 2, Supplementary Table S2).

One notable position of a major locus was identified, i.e., the RMS4 locus (Supplementary Figure S3), controlling basal branching, which matched to the peak position of JD_18_NbBr, a branching QTL projected to MDA.7.1.

## 4. Discussion

A large part of the variation in *D. pinodes* resistance and frost tolerance is controlled, in the selected populations, by common genomic regions which also control architectural and phenological traits. Most QTLs involved in moderate to high variation in *D. pinodes* disease severity were stable across RIL populations, strains, and organs and are consistent with the variation in architectural or phenological traits in these populations. They clustered in ten metaQTLs (MDA QTLs) corresponding to small-size genomic regions controlling both disease severity and architectural traits. Six of these ten metaQTLs (MDAF QTLs) also controlled frost tolerance and involved 75% and 65% of the QTLs controlling *D. pinodes* disease severity and architectural or phenological traits, respectively. Some additional genomic regions were specific to the control of either *D. pinodes* disease severity (Dp 3.1, Dp 3.9, and Dp7.3) or architectural traits (numerous), independently from known QTLs controlling frost tolerance.

### 4.1. New HD Genetic Maps and Phenotyping Data Improve QTL Detection Accuracy and Robustness

The new genomic data including transcriptomic PsCam and whole genomic Ps1 SNPs allowed to consistently reduce the size and to densify, with relevant markers, the confidence intervals of previously detected QTLs, which allowed to confirm or infirm their detection and to detect new QTLs. The JD-map, with 8-fold more markers than the previous map [24], allowed to re-detect QTLs controlling disease severity to the Mp 94 01 3 (S2) strain on plantlet stipules and stems on LGIII, LGV, LGVI, and LGVII (Table 2). However, it did not allow to re-detect mpII-1, mpII-2, nor any other QTL controlling *D. pinodes* disease severity on LGII, probably due to a higher stringency in QTL detection conditions. Marker densification also revealed a recombination gap in the central genomic region of LGVI (97 markers mapping at two very close bins), specific to the JD-map, suggesting a local rearrangement of chromosome structure in this region. This may be due to a paracentric inversion between the two parents of the population, such as the ones described by SNP-derived haplotype patterns in *Arabidopsis thaliana* [53] or by chromosome conformation capture sequencing in barley [54]. Evidence of similar inversions associated with a lack of recombination that contained key agronomic genes such as resistance to biotic stresses has also recently been demonstrated in *Brassica* genomes [55]. JI296 and DP are genetically distant [56], but both are considered as belonging to the same species, i.e., *P. sativum*. Translocations and transpositions have long been known to contribute to *Pisum* evolution [41], but reports of such an inversion in the genus are rare, apart from one in *P. sativum abyssinicum* [57], which is now classified as *P. abyssinicum,* i.e., as a species distinct from *P. sativum* [58].

The CT-map comprised 12-fold more markers than the one from Loridon et al. [43] previously used by Lejeune-Hénaut et al. [30] and Dumont et al. [31] to detect QTLs controlling frost tolerance and phenological traits. QTLs previously detected on LGIII (FR.3.1 and FR.3.2, corresponding to the WFD 3.2 and WFD 3.1 regions, respectively), LGV (FR.5.1 and FR.5.2, both included in the WFD 5.1 region), and LGVI (FR.6.1 and FR.6.2, both included in the WFD 6.1 region) were confirmed, whereas WFD1.1 and QTLs for frost tolerance on LGI [30] were not, probably due to more stringent conditions used here for QTL detection (Table 2).

The creation, genotyping, mapping, and phenotyping of a new population (JIxFP) that did not segregate for the Hr and Le major genes confirmed in a new genetic background the presence of QTLs controlling *D. pinodes* disease severity on LGV and LGVI, detected a new one on LGVII, but interestingly showed no QTL detection on LGIII, where the *Hr* and *Le* major genes map. It also allowed to detect QTLs controlling plant height specific to this population on LGIII and LGVII.

Finally, the new phenotypic data for *D. pinodes* disease severity (from two new strains and detached stipules conditions data for the JIxDP population, from all strains and all conditions for the other two populations) and for most architectural and phenological traits, combined with the high level of common and colinear markers between the three maps, allowed to strengthen QTL detection across strains, evaluation conditions, and populations (Table 2).

### 4.2. Genomic Regions Controlling D. pinodes Disease Severity Are Consistent across Strains and Organs, However, Depend upon Resistance Sources and Mapping Populations

This first report of *D. pinodes* disease severity QTLs in pea under controlled conditions using two different phenotyping methods (on whole plantlets and on detached stipules), three strains differing in their aggressiveness, and three different segregating RIL populations corresponding to different resistance sources, resulted in the detection of eighty QTLs. This analysis confirmed and extended earlier reports based on smaller sets of plant genotypes, pathogen strains, or resistance typing techniques. As previously reported [33,34], symptom development on stipules and stems of whole plantlets were highly correlated, whatever the strains and populations. The resulting QTLs were therefore generally stable between organs, enlarging a previous report [24] based on a single strain (S2) and a single segregating population (JIxDP). The QTL detection also confirmed that partial resistance on both organs at the plantlet stage is largely under a common genetic control. Flecks coalescence 2 and 3 days after inoculation and AUDPC for lesion extension on detached stipules were also, for each strain x population combination, highly correlated on a reduced set of independent genotypes [32], resulting in a number of QTLs common to both resistance components (Dp.3.2 and Dp.5.1 for S1 in ChxTe, Dp.3.3 for S3 in JIxDP, Dp.6.2 for S2 in ChxTe, Dp.7.3 for S1 in JIxFP). Lower but still significant correlations between traits assessed on detached stipules and on plantlets within RIL populations corroborated significant correlations previously reported on a smaller set of independent genotypes [32]. MetaQTLs belonging to MDAFs (Dp.3.2, Dp.3.3 and Dp5.1, Dp.5.2, [Dp.6.1/Dp.6.2], and Dp.6.3), detected with both assessment methods, suggest that disease severity assessment on whole plantlets partly depends upon the control of resistance components at the organ level. Other metaQTLs, however, seemed specific to one or the other phenotyping method, on whole plantlets (such as Dp.3.1, Dp.5.3, and Dp.7.1), or on detached stipules (such as Dp.3.9 and Dp.7.3).

Comparison of the 22 identified metaQTLs’ locations with previous reports on *D. pinodes* partial resistance under either controlled and/or field conditions was facilitated by the availability of common molecular markers between maps [24,27,28,59]. However, it remained hypothetical for other reports [23,26,60,61,62].

On LGI, Dp.1.1 and Dp.1.2, specific to the ChxTe population, and Dp.1.3, specific to the JIxDP population (with a contribution to reduced disease severity from the susceptible parent), correspond to no previously identified QTL by Prioul et al. [24] or Fondevilla et al. [27,59]. They may, however, match the Asc1.1 and abI-IV1 QTLs detected in Timmerman-Vaughan et al.’s [23,61] and Jha et al.’s [26] reports, respectively.

On LGIII, Dp.3.3 most likely corresponds to the mpIII-3 and MpIII.3 QTLs from Prioul et al. [24] and Fondevilla et al. [27,59], respectively, and may correspond to abIII-1 and to Asc.3.1 from Timmerman et al. [23,61,63] and Jha et al. [26,62], respectively. Dp3.4 on LGIII most likely corresponds to mpIII-1 identified by Prioul et al. [24] and may correspond to MpIII.1 identified by Fondevilla et al. [27,59]. Dp.3.1, specific to the ChxTe population, probably corresponds to abIII-2 from Jha et al. [26]. These two QTLs are genetically close to but apparently distinct from a QTL controlling plant height, while the closely linked Dp.3.2, also specific to the ChxTe population (and bringing resistance from the susceptible parent), mapped to a QTL controlling plant height tightly linked to the *Le* gene, which, therefore, could not correspond to abIII-2. Each of Dp3.1, Dp3.2, and Dp3.4 could correspond to the Asc3.2 QTL position identified by Timmerman et al. [61,63]. Dp3.7 and Dp3.8, both specific to the JIxDP population, most likely correspond to mpIII-4 and mpIII-5 from Prioul et al. [24] but were not identified in other studies. As of Dp3.5 and Dp3.6, both specific to the JIxFP population and with resistance brought by the susceptible parent, and Dp3.9, specific to the ChxTe population, they do not seem to match any counterpart in previous reports.

On LGV, Dp.5.1/Dp.5.2/Dp.5.3 all correspond to the mpVa-1 large region identified by Prioul et al. [24] and may well correspond to the MpV.1 and Asc5.1 regions identified by Fondevilla [27,59] and Timmerman [23,63], respectively.

On LGVI, Dp.6.1 likely corresponds to mpVI-1 identified by Prioul et al. [24] and may correspond to MbVI identified by Tar’an et al. [60]. The lack of recombination in this region and the subsequent approximate projection of the QTLs in this area on the composite map make it likely that Dp.6.1 and Dp.6.2 should have mapped at the same position (see Section 3.4.3).

On LGVII, Dp.7.1 corresponded to mpVII-1 identified by Prioul et al. [24], but due to the lack of common markers, its correspondence to the Asc7.1, Asc7.2, Asc7.3, or abVII.1 and abVII.2 QTLs identified by Timmerman et al. [61] and Jha et al. [26] remains hypothetical (Table 4).

Finally, unlike most previous reports [26,61], we detected no QTL controlling disease severity on LGII and LGIV.

Discrepancies between reports may be due to the nature of crosses and resistance progenitors, some considering interspecific crosses with *Pisum fulvum* [26] or *P. syriacum* [27,28,59] as a source, others dealing with crosses within *P. sativum* but involving distant cultivated types (forage or garden pea). They could also result from differences in experimental conditions, which ranged from multiple overlapping cycles of the whole “natural” *Ascochyta* blight complex on maturing plants in the field to monocyclic epidemics under controlled inoculations with monoporic *D. pinodes* strains on plantlets under controlled conditions.

Although many collocations between studies still remain hypothetical, the use of range of conditions (controlled and field), the mapping of common markers and their anchoring on the pea reference genome [41], as proposed for Asc QTLs by Timmerman-Vaughan et al. [61] and for ab QTLs by Jha et al. [26], and the inclusion of GWAS data will help to clarify the involvement of major genomic regions across studies, populations, assessment conditions, and strains (Table 4).

### 4.3. Co-Control of D. pinodes Disease Severity, Frost Tolerance, and Architectural or Phenological Traits in Five Regions of the Pea Genome

#### 4.3.1. *D. pinodes* Resistance and Frost Tolerance Were Associated with a Delayed Flowering and a Higher Number of Basal Branches

At four MDAF regions (MDAF.3.1, MDAF.3.2, MDAF.5.2, and MDAF.6.2), the same parental alleles controlled higher *D. pinodes* resistance, higher frost tolerance, and higher number of basal branches (Table 3). Such colocalizations between *D. pinodes* resistance and architectural traits were previously reported for two regions of LGIII, namely MpIII.1 and MpIII.3, where QTLs for *D. pinodes* resistance coincided with two QTLs controlling aerial plant biomass in the field, assessed as plant volume, leaf area, and stem area altogether using a unique visual evaluation [27]. The allelic co-variations were, however, partly different, since a higher biomass, associated with a higher number of branches, was correlated to a higher *D. pinodes* resistance only for MpIII.1 [27]. The *D. pinodes* resistance QTLs underlying the MDAF regions discussed here rely on the observation of isolated plantlets or detached stipules under experimental protocols which make the disease scoring independent from the canopy architecture effect. This result therefore supports the hypothesis of a pleiotropic effect of the four MDAF regions on both architecture and disease resistance. In any case, as MDAF.3.2, MDAF.5.2, and MDAF.6.2 coincide with positions identified under field conditions ([24], Table 2), these regions can be considered as significant genetic determinants of *D. pinodes* resistance.

Colocalizations between QTLs controlling disease partial resistance and flowering traits have been observed in many other pathosystems. The allele contributing to disease resistance was, in some cases, associated with earlier flowering, such as in *Fusarium* head blight resistance and heading date in barley [64], white mold disease severity (due to *Sclerotinia sclerotiorum)*, and flowering precocity in bean [65] or resistance to *Ascochyta* blight (*Ascochyta rabiei* (Pass.) Labr.) and flowering time in an interspecific cross within the *Cicer* genus [66]. In some other cases, the disease resistance allele was coupled with later flowering, such as for *Ascochyta* blight resistance in a *Cicer arietinum* intraspecific progeny [67]. In pea, lower *Ascochyta* blight severity has previously been observed in late and intermediate maturity cultivars [68]. Moreover, in the progeny of a cross between a *P. sativum* ssp. *syriacum* accession and the *P. sativum* spp. *sativum* cultivar Messire, Fondevilla et al. [27] identified three QTL colocalizing regions where alleles controlled both *D. pinodes* resistance and late flowering. Two of these regions (MpIII-1 and MpIII-3) likely correspond to the metaQTLs MDAF3.1 and MDAF3.2 identified in the present study, as evidenced by common or closely linked SSR markers (A6 for MDAF3.1, Figure 2; AA375 for MDAF3.2, Figure S1), while the third (MpVI-1) likely matches MDAF6.2 based on the central position of this QTL on LGVI on both maps. Allele associations reported by [27] were identical to those observed in the present study for the regions corresponding to MDAF3.2 and MDAF6.2, the parental line contributing disease resistance also contributing a later flowering date. Our study confirms that at least three regions, accounting together in our populations for about 55–56% of the variation in *D. pinodes* disease severity reduction and frost tolerance, respectively, also control a delay in flowering.

#### 4.3.2. *D. pinodes* Resistance and Frost Tolerance Are Associated with Small Stipules

At two metaQTLs (MDAF.5.1 and MDAF.6.2), the same parental alleles controlled higher *D. pinodes* resistance, higher frost tolerance and shorter stipule length (Table 3). Both metaQTLs were already known to control partial resistance to *D. pinodes* [24,59], frost tolerance [29], but also partial resistance to *Aphanomyces euteiches* [69] in pea, but their effect on stipule size was not yet described. A third metaQTL on LGI (MDA.1) did control *D. pinodes* resistance increase together with stipule length reduction, but not frost tolerance. Our observations tend to mirror those made in chickpea, where a lower *Ascochyta* blight (*D. rabiei*) severity was observed on pinnate-leaved genotypes compared to unifoliate types and attributed to a morphological disadvantage of the unifoliate, larger leaves, on which infection can spread without interruption [70]. Large-leaved clover accessions were similarly shown to be more susceptible to infection by *Stemphylium* sp. because their leaves capture more water and retain it longer, which increases disease incidence [71]. Frost tolerance is also known to be related to morphological parameters such as plant height, length of internodes, and leaf size [72]. Thus, under short days and low temperatures, frost-tolerant genotypes of herbaceous species may exhibit a rosette morphology, i.e., short internodes and small leaves, in flax [8], alfalfa [9], and pea [10,30], while susceptible cultivars grown in the same conditions have longer stems and larger leaves. In winter wheat, Jaskune et al. [73] showed that the dynamics of leaf elongation during the acclimation period was correlated with freezing tolerance, the slow-growing cultivars being more tolerant to frost than the fast-growing ones, sustaining the hypothesis of a genetic advantage to growth cessation at low temperatures increasing tolerance to subsequent frost stress. Histological differences could also contribute to the advantage conferred by smaller leaves, which show a higher vein length per leaf area, this trait being associated with a greater ability to transport water and a lower vulnerability to freezing and dehydration [74,75].

#### 4.3.3. The Major Gene *Le* Colocalizes with Both *D. pinodes* Resistance and Frost Tolerance QTLs

MetaQTLs Dp.3.2, A.3.1, and FR.3.1 all included the *Le* gene, controlling internode elongation, as their major peak. Both *D. pinodes* resistance and frost tolerance QTLs were detected in populations segregating for *Le* in this region. The favorable allele at this locus in population ChxTe was provided by Térèse, susceptible to both *D. pinodes* and frost, but was a carrier of the *le* (dwarfism) allele. The colocalization between *Le* and frost tolerance QTLs has also been observed in both biparental and association mapping populations in pea (Table 2). Beji et al. [52] showed that the *Le* gene bore one of the three significant markers identified at the corresponding frost tolerance locus, which makes it a potential causal candidate. A direct effect of small internodes on frost tolerance could rely on a position closer to the ground, allowing plants to benefit from milder temperatures during the winter [10]. Dwarfism of internodes and foliar organs is a component of the rosette morphology, mentioned in the previous section as an architectural advantage regarding frost tolerance. In pea, dwarfism of aerial organs is even observed during the winter period for frost-tolerant *Le* genotypes, like Champagne, the high phenotype of which is expressed only during the following spring. The effect of *Le* on *D. pinodes* resistance seems to be more complex. In the controlled conditions of the present study (individual plantlets or organs and assessment of a single cycle of the pathogen), the *le* allele is clearly associated with a higher *D. pinodes* resistance (Table 3, Supplementary Table S2). This potential favorable effect of the *le* allele may, however, have a limited effect on adult plants in canopy conditions in the field, where higher genotypes were found to limit pathogen dispersal through splashing [76] and to limit disease progression thanks to more porous canopies, where the microclimate is less favorable to the fungus [77]. In common bean (*Phaseolus vulgaris* L.), damages due to white mold (*S. sclerotiorum*) are also negatively correlated to canopy height and positively correlated to canopy porosity [78].

From a physiological point of view, the *Le* locus could have pleiotropic effects on plant architecture, *D. pinodes* resistance, and frost tolerance. The *Le* allele indeed encodes a gibberellin (GA) 3β-hydroxylase that is able to convert GA_20_ into the bioactive GA_1_ producing long internodes [79]. Impairment of *Le* expression, either due to the *le* mutation or to interactions with other genes or environmental conditions, would reduce bioactive GA levels, thus promoting the accumulation of DELLA proteins, which may in turn modify both plant architecture and stress responses. For example, during the cold acclimation process of *A. thaliana*, a reduction in bioactive GA was shown to allow a higher accumulation of DELLA proteins, which concomitantly restrained plant growth and promoted freezing tolerance [80]. In parallel, dwarf gain-of-function DELLA lines of barley and wheat were shown to be more resistant to necrotrophic pathogens (*Fusarium graminearum* and *Oculimacula* species) than their tall loss-of-function counterparts [81]. It is, however, observed that the effect of higher DELLA proteins levels may vary according to the pathogen trophic style (biotrophic, hemibiotrophic, or necrotrophic), and the trade-off between plant stature and disease resistance thus has to be carefully checked in plant breeding.

#### 4.3.4. The Major Gene *Hr* Colocalizes with Both *D. pinodes* Resistance and Frost Tolerance QTLs

The *Hr* (High response to photoperiod) gene was mapped on LGIII and corresponded to the peak of MDAF.3.2. and of each A.3.2, Dp.3.3 and FR.3.2 specific metaQTLs. *Hr* is an ortholog of *ELF3* (EARLY FLOWERING 3), a gene involved in circadian clock function [82]. Under short days, the *Hr* allele is known to delay floral initiation and flowering, to increase the number of branches, and to decrease leaf area, thus determining the rosette-type growth habit [30,82]. This genomic region is also known to govern resistance to biotic and abiotic stresses in pea, such as frost tolerance [29,30] and *D. pinodes* resistance [24], the corresponding QTLs peaking on the same group of markers of the composite map used for QTL detection in the present study (Figure 2). Moreover, QTLs for partial resistance to root rot diseases were also projected in the same region. Both an allele for resistance to *A. euteiches* [69] and an allele for resistance to *Fusarium solani* [83] correspond to the Champagne and DP alleles of the ssr marker AA175 and of the SNP marker Ps900043, respectively, both linked to *Hr*. The *Hr* gene is also a causal candidate for frost tolerance since the delayed floral initiation determined by the dominant allele under short days favors an escape mechanism to late winter freezing periods [84]. *Hr* has been more precisely shown to influence pea sensitivity to low red: far red ratio [82]. This role in light input to the circadian clock could be a common determinant of the cold and biotic stress responses, as suggested by Roeber et al. [85]. This potential signaling part of *Hr* in response to *D. pinodes* deserves further exploration. Interestingly, two other metaQTLs, namely MDAF.6.2 and MDAF.3.2, also show close patterns of control of resistance and tolerance with common architectural traits. All three of these regions showing tight colocalizations on three different LGs suggest either a pleiotropic effect of one or some major genes controlling two or three of these traits or tight genetic linkages. Further investigation of metaQTL underlying genes sequences and regulation will be needed to address this issue.

#### 4.3.5. Underlying Positional, Expressional, or Functional Candidate Genes in MDAFs’ Confidence Intervals

Many of the genetically mapped molecular markers that fall within MDAFs’ confidence intervals are SNPs (PsCam fromTayeh et al. [38], Ps0 and Ps9 from Duarte et al. [39]), eSSRs, or ESTs (Genoplante project, unpublished) developed from genes (or DNA sequences homolog to genes) of known functions and may thus be considered as both positional and/or functional candidates. Some of them, mainly corresponding to Ps9 markers, are expressional candidates under abiotic stress, i.e., winter hardiness, or biotic, i.e., fungal inoculation, contrasted conditions (Supplementary Table S3*).* Putative functions of these genes neighboring MDAFs’ peak position include hormonal signaling pathways, responses to oxidative and other stresses, responses to photoperiod or vernalization for flowering, and primary metabolism. Numerous other genes lie within MDAFs’ confidence intervals; therefore, assigning a role to one or the other of these genes on the observed phenotypes remains highly hypothetical. The anchoring of the PsCam markers on the Cameor reference genome [41] (Supplementary Table S3) is a first step to future links between MDAF regions and the pea physical map. It will allow positional candidate genes to be more reliably hypothesized as soon as a new version of the Cameor genome, as well as the release of Champagne and Terese genomes, becomes available. The coincidence of MDAF confidence intervals with four of the five DTF (days to flower) QTLs identified by Williams et al. [86] is, however, noticeable. On LGIII, MDAF.3.1 and MDAF.3.2 correspond to Williams’ DTF5b and DTF5a and highlight the same candidate genes involved in plant development and architecture, i.e., *Le* and *Hr*, respectively. On LGVI, MDAF.6.2 overlaps with the flowering QTL DTF1 and comprises the candidate FT (florigen) gene, namely *FTa3* identified in Williams et al. [86]. On LGV, MDAF.5.1 partially overlaps with Williams’ DTF3 but does not contain the candidate FT genes *FTa1* and *FTc*. Williams et al. [86] demonstrated that DTF1, DTF3, and DTF5a contribute to earlier flowering in a domesticated *P. sativum sativum* cultivar vs. a wild *P. sativum humile* line. In the present study, the forage parents Champagne and DP, which are genetically close to *P. humile* [56], could have inherited, from wild ancestors, the late flowering alleles at these QTLs. They could also have inherited frost tolerance and *D. pinodes* resistance alleles either by genetic linkage or by pleiotropic effect of the flowering genes when included in the QTL confidence intervals [86].

A pleiotropic effect of some candidate genes on the number of branches and on *D. pinodes* resistance must be considered to elucidate the genetic determinism of these traits. Any positive genetic relationship between a high number of branches and the level of *D. pinodes* resistance could, however, be counterbalanced by a negative effect of profuse basal branching at the adult stage on fungal infection [77].

### 4.4. Impact of Colocalizations for Breeding

The large observed colocalizations of, and allelic variations at, QTLs controlling partial resistance to *D. pinodes* and tolerance to frost with QTLs or genes controlling the architecture or development of the plant, whatever its origin (gene pleiotropy or genetic linkage), is a major element to be considered in future breeding programs. Our experimental conditions are in some cases restricted to scales (organs or individual plantlets for *D. pinodes* resistance and architectural traits) and conditions for the epidemics (*D. pinodes* monocycle in controlled conditions) that may be partly modified in field plots (adult plants in canopies, likely multiple cycles of the pathogen and interactions with other pathogens of the ascochyta complex, genotype interactions with the environment and agricultural practices). A large plasticity of both disease resistance and architectural traits in canopies in the field is thus likely, including the emergence of epidemiological mechanisms linked to canopy architectural traits, such as microclimate and plant ageing gradients within the canopy that modify disease severity assessments [3,77]. The comparison of identified MDAF regions with previously identified QTLs from field experiments, however, shows that considering them in breeding may be of interest to accumulate resistance alleles in pea varieties.

Our results clearly identify target genomic regions for breeding and support the choice of alleles at these regions to reach compromises allowing a multiplicity of challenges related to stress response in various environmental conditions to be tackled. Traits that may be of interest for the breeding of winter pea ideotypes include late flowering, short plants, a reduced number of nodes, short stipule size, and a high number of branches, which could indirectly promote both quantitative resistance to *D. pinodes* and frost tolerance. Finding the right compromises and the right type of association between partial resistance, tolerance, and architectural traits at each MDA and MDAF QTL will determine its interest in terms of breeding. Finally, several identified *D. pinodes* resistance population-specific QTLs which are not strongly linked to the studied architectural and phenological traits may also be useful in pyramiding favorable alleles in breeding programs.

## Figures and Tables

**Table 1:** Number and density of markers, number of gaps between contiguous markers, and length per linkage group and on the whole pea composite map constructed from the CT-, JD-, and JF-maps.

	LGI	LGII	LGII	LGIV	LGV	LGVI	LGVII	Whole
Number of markers	271	382	572	396	348	373	402	2744
Number of KASP SNPs	174	235	405	268	255	222	253	1812
Number of Markers/cM	2.7	2.9	3.7	2.9	2.6	3.3	2.8	3.0
Number of KASP SNPs/cM	1.7	1.8	2.6	2.0	1.9	2.0	1.8	2.0
Number of gaps > 2 cM between two contiguous markers	9	8	11	17	13	8	13	79
Number of gaps > 5 cM between two contiguous markers	0	0	0	0	3	0	2	5
Length (cM)	101.8	131.4	155.8	137	136.4	111.5	141.3	915.2

**Table 2:** MetaQTL detections, including for each metaQTL (MDA, MDAF, Dp, FR, A), the peak position and the size of the confidence interval [CI] in CM, the number and the type of underlying one-trait QTLs in each population, the parental resistant allele, and the maximal coefficient of determination R^2^ max as a percentage of the explained variance.

LG	MDA/MDAF QTL (Whole Analysis)	MDA/MDAF Peak (cM)	MDA/MDAF [CI] (cM)	MetaQTL (Analysis by Trait)	MetaQTL Peak (cM)	MetaQTL [CI] (cM)	Nb QTL/ChxTe	Nb QTL/JIxDP	Nb QTL/JIxFP	Parental Resistant Allele	R^2^ Max (%Var)
**LG I**	MDA.1.1	90	3	Dp.1.2	90	3	1	0	0	Champagne	27.5
				A.1	90	3	StLe 1	0	0	NA	38.9
**LG III**	MDAF.3.1	152.1	0.9	Dp.3.2	152.1	1.5	3	0	0	Terese	26.3
				A.3.1	152.2	1.1	dflo 2	*	*	NA	1.6
							NbBr 1	NbBr 1	0		54.1
							StLe 1	0	0		10.3
							Ht 1	Ht 1	0		91.6
							NbNo 1	NbNo 1	0		7.19
				FR.3.1	152.1	1.6	FRfd 2	*	*	Terese	7.4
	MDAF.3.2	29.6	0.3	Dp.3.3	29.8	1.4	3	5	0	Champagne DP	19
				A.3.2	29.8	0.6	dflo 3	*	*	NA	68.9
							0	NbBr 1	0		19.3
				FR.3.2	29.6	0.4	FRfd 11	*	*	Champagne	60.2
**LGV**	MDAF.5.1	68.9	0.8	Dp.5.1	68.9	0.9	6	1	6	Champagne DP FP	36.8
				A.5.1	65	4.6	0	0	StLe 1	NA	23.5
				A.5.2	69.1	2.5	StLe 1	StLe 1	0	NA	5.9
				FR.5.1	67.7	2.3	FRncc 1	*	*	Champagne	21.1
							FRfd 3	*	*	Champagne	7.8
	MDAF.5.2 (MDAF.5.2.1 + MDAF.5.2.2)	74.4 (72.0/76.6)	1.7 (1.6/2.6)	Dp.5.2	72.5	2.1	4	2	2	Champagne DP FP	19.6
				FR.5.2	71.8	3	FRfd 4	*	*	Champagne	13.3
				A.5.3	74.8	7	NbBr 1	0	0	NA	6.3
				Dp.5.3	77.4	3.1	0	5	0	DP	18.7
				FR.5.3	78	1	FRfd 1	*	*	Champagne	5.2
**LGVI**	MDA.6.1	34	3.2	Dp.6.1	34	3.3	0	7	0	DP	29.4
				A.6.1	56.1	1.2	0	StLe 1	0	NA	26.9
	MDAF.6.2	56.9	0.7	A.6.1	56.1	1.2	dflo 3	*	*	NA	3.5
							StLe 1	0	0		26.9
				Dp.6.2	57.4	1	5	0	9	Champagne FP	35.1
				FR.6.1	55.8	2.3	FRfd 10	*	*	Champagne	14.4
				A.6.2	59.1	2.6	0	0	StLe 1	NA	28.6
							NbBr 1	0	0		5.8
	MDAF.6.3	64.4	2.5	A.6.2	59.1	2.6	0	NbBr 1	0	NA	5.8
				FR.6.2	63.5	3.6	FRncc 1	*	*	Champagne	14.5
				Dp.6.3	65.3	3.6	2	0	0	Champagne	29.3
**LGVII**	MDA.7.1	68.3	1.3	Dp.7.1	68.3	1.3	0	2	0	DP	35
			1.3	A.7.1	68.3	6.7	0	NbBr 1	0	NA	11.2
	MDA.7.2	81.2	12.7	Dp.7.2	93	13.6	0	0	1	FP	13.9
			12.7	A.7.2	81.2	12.7	0	0	StLe 1	NA	14.6

FRncc = Frost controlled conditions; FRfd = Frost field conditions; other abbreviations are as in Figure 1. 0 = no QTL detected; * dflo and FR data not available for JIxDP and JDxFP; NA = Not applicable for A traits. Red, green, and blue backgrounds refer to D. pinodes, architecture, and frost data, respectively.

**Table 3:** Allelic co-variations between *D. pinodes* resistance, frost tolerance, and architectural traits at major MDAF QTLs. Described effects correspond to stress resistant/tolerant (Champagne, DP, and FP, in black) or susceptible (Terese, in red) parental alleles.

		Increase in *D. pinodes* Resistance and Frost Tolerance				
**Increase in**	**Number of branches**	MDAF.3.1	MDAF.6.2	MDAF.3.2	MDAF.5.2	
**Delay in**	**Flowering date**	MDAF.3.1	MDAF.6.2	MDAF.3.2		
**Decrease in**	**Stipule length**	MDAF.3.1	MDAF.6.2			MDAF.5.1
**Decrease in**	**Plant height**	MDAF.3.1				
**Decrease in**	**Number of nodes**	MDAF.3.1				

**Table 4:** Comparison of LG- locations of reported QTLs from biparental populations or gwas panels for *D. pinodes* resistance and frost tolerance in various assessment conditions.

Population(s)	GWAS	China x Cameor	Champagne x Terese	Champagne x Terese	JI296 x FP	JI296 x DP	JI296 x DP	P665 x Messire	P665 x Messire	A88 x Rovar	A26 x Rovar	A88 x Rovar/A26 x Rovar	Carneval x MP1401	P651 (*P.fulvum*) x Alfetta
**Disease assessment conditions**				**Controlled, inoculated, seedlings, **	**Controlled, inoculated, seedlings, **	**Controlled, inoculated, seedlings, **	**Controlled, Field inoculated, seedlings, adult plants **	**Controlled, Field inoculated, seedlings, adult plants **	**Controlled, Field inoculated, seedlings, adult plants **	**Field, natural epidemics, adult plants **	**Field, natural epidemics, adult plants **	**Field, natural epidemics, adult plants **	**Field, natural epidemics, adult plants **	Field, natural epidemics, adult plants ** + Controlled, inoculated, seedlings ***
**Frost assessment conditions**	**Field and Controlled**	**Field**	**Field and Controlled**	**Field and Controlled**										
**LG I**			(WFD 1.1)							Asc1.1		Asc1.1		abI-IV-1 **
	LDBlock I.1													
				(Dp.1.1)										
				(Dp.1.2)										
						(Dp.1.3 *)								
**LG II**							mpII-1, mpII-2	MpII.1	MpII.1	(Asc2.1)	Asc2.1, Asc2.2	Asc2.1	MbII	
**LG III**		III.1		Dp.3.1				MpIII.1			Asc3.2 ?	Asc3.2 ?		abIII-2 ***
	LDBlock III.1	III.1	WFD 3.2 *	FR.3.1 */Dp.3.2 *										
									MpIII.1					
						(Dp.3.4)	mpIII-1							
					(Dp.3.5 *)									
					(Dp.3.6 *)				MpIII.2					
						(Dp.3.7)	mpIII.4 ?		MpIII.4					
						(Dp.3.8)	mpIII-5							
			WFD 3.1	FR.3.2/Dp.3.3		Dp.3.3	mpIII-3	MpIII.3	MpIII.3	Asc3.1 ?	Asc3.1 ?	Asc3.1 ?		abIII-1 **
				Dp.3.9										
							mpIII-2		MpIII.5 ?					
**LG IV**								MpIV.1	MpIV.1	Asc4.1, Asc4.2, Asc4.3	Asc4.1	Asc4.2, Asc4.3	MbIV	abI-IV-2 **, abI-IV-3 **, abI-IV-4 **, abI-IV-5 ***
**LG V**		V.1												
	LDBlock V.1		WFD 5.1											
				FR.5.1/Dp.5.1	Dp.5.1	Dp.5.1	mpVa-1	Mp.V.1	MpV.3	Asc5.1	Asc5.1			
				FR.5.2/Dp.5.2	Dp.5.2	Dp.5.2								
						Dp.5.3			MpV.2					
		V.2							VpV.1					
			(WFD 5.2)	(FR.5.3)										
**LG VI**														
	LDBlock VI.1/LDBlock VI.2	VI.1	WFD 6.1	FR.6.1/Dp.6.2	Dp.6.2	Dp.6.1	mpVI-1	mp.VI.1	mp.VI.1				MbVI	
				FR.6.2/Dp.6.3										
				(Dp.6.4 *)										
**LG VII**		VII.2								Asc7.1	Asc7.3, Asc7.1, Asc7.2			abVII-1 **, abVII-2 ***
	LDBlock VII.1													
						Dp.7.1	mpVII-1							
					(Dp.7.2)									
					Dp.7.3									
**LG I**	Beji et al. 2020 [52]	Klein et al. 2014 [29]	Lejeune et al. 2008 [30] /Dumont et al. 2009 [31]	This study			Prioul et al. 2004 [24]	Fondevilla et al. 2008 [59] / Fondevilla et al. 2011 [27]	Carrillo et al. 2014 [28]	Timmerman-Vaughan et al. 2002 [23]	Timmerman-Vaughan et al. 2004 [63]	Timmerman-Vaughan et al. 2016 [61]	Ta’ran et al. 2003 [60]	Jha et al. 2016 [26]

( ): not reliable or weak reproducibility across conditions (only one variable, i.e., only one strain in one condition on one population); ?: Approximate position or no common marker to confirm colocalization; *: Stress resistance/tolerance allele is carried by the susceptible parent; **: Field, natural epidemics (Jha et al., 2016 [26]); ***: Controlled, inoculated (Jha et al., 2016 [26]). Data corresponding to frost tolerance previous publications are in blue. Data corresponding to *D. pinodes* resistance previous publications are in red.

## Data Availability

The phenotyping raw data presented in this study are associated with previous publications and are available on request from the corresponding author. The newly generated RILs’ genotyping raw data are not publicly available due to privacy restrictions.

## Supplementary Materials

The following supporting information can be downloaded at: https://www.mdpi.com/article/10.3390/genes14071399/s1, Supplementary File S1—Pathogenicity tests; Figure S1—Colinearity of the markers common to the CT-(left) JF-(center) and JF-(right) maps. Distances are in cM (Haldane); Table S1—Broad sense heritability for disease severity under controlled conditions and architectural traits variables for ChxTe, JIxDP, and JIxFP RIL populations; Figure S2—QTL projection on each JD-(left) CT-(center) and JF-(right) maps. Distances are in cM (Haldane). QTL nomenclature is “Map-Id_Trait-Id_R^2^”. *D. pinodes* resistance QTLs are in red, frost tolerance QTLs are in blue, and architectural/phenological QTLs are in green; Table S2—Detailed information for each of the 153 detected QTLs, including: linkage group name, corresponding metaQTL, QTL name, position, confidence interval, LOD score, R^2^ and parental effect, corresponding MDA/MDAF metaQTL if any; Figure S3—QTL projection on the composite map. Distances are in cM (Haldane). QTL nomenclature is “Map-Id_Trait-Id_R^2^”. *D. pinodes* resistance QTLs are in red, frost tolerance QTLs are in blue, and architectural/phenological QTLs are in green; Table S3—Putatives genes coresponding to molecular markers closest to the MDAF peaks. Information including genetic position, putative corresponding gene and bibliographic reference, for each marker, physical position for PsCam markers.
