# Supplementary material for: Five Regions of the Pea Genome Co-Control Partial Resistance to D. pinodes, Tolerance to Frost, and Some Architectural or Phenological Traits
Source: Genes (Basel). 2023 Jul 4;14(7):1399. doi: 10.3390/genes14071399 (PMC10379203; doi:10.3390/genes14071399)
Supplement: Supplementary file 1 [file genes-14-01399-s001.zip › genes-2286623 - Table S2.pdf]

LG name	MetaQTL (Individual stress analysis)	QTL name	QTL position (cM)	Start Confidence Interval (cM)	End Confidence Interval (cM)	Confidence Interval size (cM)	LOD score	r2 (%Var)	Parental effect [LsMeans"R parent" - LsMeans"S parent"]	MDA/MDAF-QTL
LGI	Dp.1.1	CT_1_DS_STIP_S2	85.0	80.7	87.1	6.4	5.4	7.8%	-5.5	none
LGI	A.1.1	CT_70_StLe	90.0	87.5	90.9	3.4	39.8	38.9%	-6.3	MDA.1.1
LGI	Dp.1.2	CT_31_DS_LE_S3	90.1	87.5	93.8	6.3	6.7	27.5%	-28.8	
LGI	Dp.1.3	JD_8_FC_2D_S3	95.5	91.5	100.4	8.9	4.9	19.3%	0.2	none
LGII	A.2.1	CT_78_dfloRE02	50.0	49.4	52.1	2.7	13.0	4.5%	10.3	none
LGII	A.2.1	CT_81_dfloMO02	52.1	49.4	54.8	5.4	18.6	7.0%	7.5	none
LGII	A.2.2	JF_25_NbBr	72.0	69.7	78.1	8.4	6.2	10.7%	0.4	none
LGIII	Dp.3.9	CT_28_DS_LE_S2	18.9	16.7	22.6	5.9	4.8	16.0%	-15.7	none
LGIII	Dp.3.9	CT_32_DS_LE_S3	20.1	16.7	20.8	4.1	4.2	16.3%	-21.2	none
LGIII	Dp.3.3	CT_18_FC_2D_S1	27.4	25.3	32.8	7.5	4.9	15.0%	-0.3	
LGIII	Dp.3.3	JD_5_FC_3D_S2	29.2	28.2	31.9	3.7	5.0	15.6%	-0.4	
LGIII	FR.3.2	CT_40_mon0203	29.3	28.2	30.8	2.6	13.0	23.0%	-0.9	
LGIII	FR.3.2	CT_61_lus0102	29.3	29.1	29.8	0.7	34.9	60.2%	-1.4	
LGIII	FR.3.2	CT_37_mon0102	29.4	29.1	30.5	1.4	23.5	31.2%	-1.4	
LGIII	Dp.3.3	JD_9_FC_2D_S3	29.4	26.8	32.8	6.0	4.2	16.3%	-0.2	
LGIII	Dp.3.3	JD_11_DS_LE_S3	29.4	26.8	32.8	6.0	3.9	18.3%	-39.1	
LGIII	FR.3.2	CT_43_cle0102	29.8	28.8	30.5	1.7	30.6	45.7%	-1.8	
LGIII	FR.3.2	CT_46_cle0203	29.8	29.1	30.5	1.4	32.3	50.5%	-1.9	
LGIII	FR.3.2	CT_52_dij0102	29.8	29.4	30.5	1.1	35.8	41.4%	-1.7	
LGIII	FR.3.2	CT_55_dij0203	29.8	29.1	30.5	1.4	28.2	42.9%	-1.6	
LGIII	FR.3.2	CT_58_col0102	29.8	29.4	30.5	1.1	30.4	47.9%	-1.7	MDAF.3.2
LGIII	A.3.2	CT_79_dfloRE02	29.8	29.4	30.5	1.1	66.3	67.4%	39.4	
LGIII	Dp.3.3	JD_2_DS_LE_S1	29.8	28.6	31.5	2.9	5.8	18.7%	-62.9	
LGIII	A.3.2	JD_15_NbBr	29.8	28.6	32.8	4.2	9.4	19.4%	0.9	
LGIII	Dp.3.3	CT_8_DS_STIP_S1	29.9	29.4	31.8	2.4	10.7	18.6%	-8.7	
LGIII	FR.3.2	CT_33_mon0001	29.9	29.1	30.8	1.7	14.8	26.8%	-0.9	
LGIII	A.3.2	CT_76_dfloRE01	29.9	29.4	30.5	1.1	39.9	68.9%	31.2	
LGIII	FR.3.2	CT_49_dij0001	29.9	29.1	30.5	1.4	20.0	40.3%	-1.6	
LGIII	A.3.2	CT_82_dfloMO02	29.9	29.4	30.5	1.1	65.9	64.8%	23.8	
LGIII	Dp.3.3	CT_12_DS_STEM_S1	30.5	29.4	32.8	3.4	8.0	19.0%	-5.1	
LGIII	FR.3.2	CT_85_cle0001	30.5	27.9	31.8	4.0	3.2	10.3%	-0.8	
LGIII	Dp.3.3	JD_23_DS_STEM_S1	32.8	29.4	38.0	8.7	4.6	11.4%	-5.4	
LGIII	Dp.3.8	JD_7_DS_LE_S2	45.4	43.4	46.9	3.5	3.8	18.5%	-26.2	none
LGIII	A.3.4	JF_22_Ht	74.3	69.0	74.8	5.8	14.1	26.7%	-36.6	none
LGIII	Dp.3.7	JD_26_DS_STIP_S2	75.4	70.2	78.6	8.3	5.3	9.0%	-6.8	none
LGIII	A.3.3	JD_12_Ht	80.1	77.9	83.4	5.5	5.8	4.8%	0.4	none
LGIII	Dp.3.6	JF_15_FC_3D_S1	99.8	95.0	105.5	10.5	4.2	15.1%	0.3	none
LGIII	Dp.3.5	JF_13_FC_2D_S1	120.7	118.7	122.6	3.9	4.2	14.6%	0.3	none
LGIII	Dp.3.4	JD_10_FC_3D_S3	142.8	136.3	152.1	15.8	3.8	18.0%	-0.3	none
LGIII	A.3.1	JD_14_NbNo	146.8	136.3	153.5	17.2	7.1	25.6%	0.6	
LGIII	Dp.3.2	CT_16_DS_STEM_S3	152.1	150.3	154.1	3.8	5.9	14.4%	4.2	
LGIII	Dp.3.2	CT_22_DS_LE_S1	152.1	150.3	152.4	2.1	8.1	15.9%	31.5	
LGIII	FR.3.1	CT_36_mon0102	152.1	150.3	153.1	2.8	7.5	7.4%	0.9	
LGIII	FR.3.1	CT_51_dij0102	152.1	150.3	153.1	2.8	6.1	4.1%	0.9	
LGIII	A.3.1	CT_65_HT	152.1	150.3	152.4	2.1	77.4	91.6%	16.5	
LGIII	A.3.1	CT_67_NbBr	152.1	150.3	153.1	2.8	30.8	54.1%	-1.2	MDAF.3.1
LGIII	A.3.1	JD_13_HT	152.1	150.3	152.6	2.4	42.1	82.5%	15.1	
LGIII	A.3.1	CT_83_dfloRE02	152.1	150.3	153.1	2.8	4.5	1.6%	-15.5	
LGIII	Dp.3.2	CT_20_FC_3D_S1	152.3	150.2	153.0	2.8	6.3	26.3%	0.3	
LGIII	A.3.1	CT_71_StLe	152.3	150.2	153.0	2.8	16.4	10.3%	2.5	
LGIII	A.3.1	CT_84_dfloMo02	152.3	150.2	155.8	5.6	4.9	1.4%	-9.1	
LGIII	A.3.1	JD_16_NbBr	153.0	147.1	153.7	6.6	6.4	12.2%	-0.3	
LGIII	A.3.1	CT_66_NbNo	154.8	150.2	155.8	5.6	7.2	18.0%	0.4	
LGIII	Dp.3.1	CT_2_DS_STIP_S2	154.8	153.7	155.8	2.1	12.7	20.9%	-5.9	none
LGIII	Dp.3.1	CT_7_DS_STIP_S1	154.8	153.0	155.8	2.8	14.6	27.7%	-10.5	none
LGIII	Dp.3.1	CT_11_DS_STEM_S1	154.8	153.7	155.8	2.1	6.8	15.6%	-4.4	none
LGV	A.5.1	JF_28_StLe	65.0	63.0	67.6	4.6	9.3	23.5%	-2.9	
LGV	FR.5.1	CT_59_col0102	67.0	65.0	70.4	5.4	4.7	4.6%	-0.7	
LGV	FR.5.1	CT_63_NCC	67.6	66.1	69.6	3.5	8.7	21.1%	-0.4	
LGV	FR.5.1	CT_56_dij0203	68.0	66.1	71.1	5.0	7.5	7.8%	-0.8	
LGV	FR.5.1	CT_34_mon0001	68.1	66.1	71.1	5.0	5.0	7.6%	-0.6	
LGV	Dp.5.1	JD_3_DS_LE_S1	68.1	59.9	71.3	11.4	4.9	15.4%	-5.7	
LGV	Dp.5.1	JF_9_DS_STIP_S3	68.5	67.6	71.8	4.2	9.1	24.3%	-5.4	
LGV	Dp.5.1	JF_5_DS_STIP_S2	68.8	68.1	70.1	2.0	14.8	32.8%	-4.6	
LGV	Dp.5.1	JF_7_DS_STEM_S2	68.8	68.1	70.1	2.0	16.1	36.8%	-3.2	MDAF.5.1
LGV	Dp.5.1	CT_14_DS_STIP_S3	68.8	68.1	70.4	2.3	11.0	26.0%	-8.7	
LGV	Dp.5.1	CT_17_DS_STEM_S3	68.8	66.1	70.4	4.3	8.1	20.5%	-5.3	
LGV	Dp.5.1	CT_19_FC_2D_S1	68.8	66.1	71.8	5.7	6.0	18.9%	-0.3	
LGV	Dp.5.1	CT_26_FC_3D_S2	68.8	67.6	74.8	7.2	5.4	18.9%	-0.1	
LGV	A.5.2	CT_72_StLe	68.8	67.6	70.4	2.8	10.5	5.9%	-3.7	
LGV	Dp.5.1	JF_11_DS_STEM_S3	68.8	68.1	71.8	3.7	11.5	29.7%	-6.0	
LGV	Dp.5.1	CT_23_DS_LE_S1	69.0	68.1	70.4	2.3	12.7	28.6%	-51.6	
LGV	Dp.5.1	JD_4_FC_2D_S2	69.9	67.6	72.8	5.2	5.1	17.1%	-0.4	
LGV	Dp.5.1	JF_1_DS_STIP_S1	70.1	68.1	74.8	6.7	5.5	14.2%	-2.9	
LGV	Dp.5.1	CT_21_FC_3D_S1	70.4	67.6	74.8	7.2	4.8	19.4%	-0.4	
LGV	A.5.2	JD_19_StLe	71.1	68.1	74.8	6.7	6.3	16.9%	-2.6	
LGV	Dp.5.2	CT_9_DS_STIP_S1	71.1	68.8	74.8	6.0	8.0	13.2%	-9.1	
LGV	FR.5.2	CT_38_mon0102	71.1	69.6	74.8	5.2	9.5	9.8%	-0.8	
LGV	FR.5.2	CT_53_dij0102	71.1	68.1	74.8	6.7	8.2	5.7%	-0.7	
LGV	Dp.5.2	JD_1_FC_2D_S1	71.3	68.1	74.8	6.7	3.6	17.0%	-0.4	
LGV	Dp.5.2	JD_27_DS_STIP_S2	71.3	68.1	74.8	6.7	6.2	10.8%	-8.0	MDAF.5.2.1
LGV	Dp.5.2	JF_3_DS_STEM_S1	71.8	68.1	74.8	6.7	7.5	19.6%	-2.6	
LGV	FR.5.2	CT_44_cle0102	72.0	68.8	74.8	6.0	8.0	7.9%	-0.9	
LGV	Dp.5.2	CT_3_DS_STIP_S2	72.8	69.6	74.8	5.2	7.3	10.8%	-4.6	
LGV	Dp.5.2	CT_5_DS_STEM_S2	72.8	68.8	74.8	6.0	7.2	18.5%	-2.6	
LGV	Dp.5.2	CT_13_DS_STEM_S1	73.0	68.1	74.8	6.7	7.1	16.4%	-5.6	
LGV	FR.5.2	CT_41_mon0203	73.0	68.8	74.8	6.0	8.2	13.3%	-0.7	
LGV	Dp.5.2	JF_20_DS_LE_S3	73.5	70.7	76.6	5.9	5.0	15.8%	-25.8	
LGV	Dp.5.3	JD_30_DS_STEM_S2	74.7	71.3	78.1	6.8	6.8	10.6%	-6.7	
LGV	A.5.3	CT_68_NbBr	74.8	70.4	77.4	7.0	5.7	6.3%	0.4	
LGV	Dp.5.3	JD_35_DS_STEM_S3	77.0	74.8	78.7	3.9	6.5	18.7%	-5.8	
LGV	FR.5.3	CT_47_cle0203	78.0	75.1	78.6	3.5	5.5	5.2%	-0.6	MDAF.5.2.2
LGV	Dp.5.3	JD_21_DS_STIP_S1	78.1	74.8	99.6	24.8	5.2	12.4%	-4.3	
LGV	Dp.5.3	JD_24_DS_STEM_S1	78.1	74.8	99.6	24.8	5.4	13.8%	-6.1	
LGV	Dp.5.3	JD_33_DS_STIP_S3	78.1	74.8	80.2	5.4	5.1	15.3%	-4.0	
LGV	FR.5.4	CT_xx_lus0102	91.0	84.8	94.2	9.4	4.7	4.6%	-0.1	none
LGV	A.5.4	JF_26_NbBr	125.5	122.3	134.8	12.5	5.7	9.8%	0.3	none
LGV	A.5.4	CT_yy_dfloRE02	130.5	129.1	133.8	4.7	4.8	1.7%	3.4	none
LGV	A.5.4	CT_zz_dfloMo02	133.0	131.2	134.1	2.9	11.7	3.9%	3.4	none
LGVI	Dp.6.1	JD_34_DS_STIP_S3	33.8	30.8	64.3	33.5	8.3	27.1%	-5.2	
LGVI	Dp.6.1	JD_36_DS_STEM_S3	33.8	30.8	34.7	3.9	9.0	27.7%	-6.8	
LGVI	Dp.6.1	JD_6_FC_3D_S2	34.3	32.2	64.3	32.1	5.6	17.5%	-0.3	
LGVI	Dp.6.1	JD_22_DS_STIP_S1	34.3	32.2	47.0	14.7	10.8	29.4%	-5.5	MDA.6.1
LGVI	Dp.6.1	JD_25_DS_STEM_S1	34.3	32.2	41.1	8.9	8.1	21.9%	-6.3	
LGVI	Dp.6.1	JD_28_DS_STIP_S2	34.5	32.2	47.0	14.7	11.8	23.4%	-8.6	
LGVI	Dp.6.1	JD_31_DS_STEM_S2	34.5	32.2	47.0	14.7	12.1	21.1%	-7.9	
LGVI	A.6.1	JD_20_StLe	34.7	34.3	67.2	32.9	9.4	26.9%	-3.2	
LGVI	Dp.6.2	CT_4_DS_STIP_S2	54.4	50.1	56.6	6.5	6.7	10.0%	-4.0	
LGVI	Dp.6.2	CT_6_DS_STEM_S2	54.4	50.1	56.6	6.5	4.8	11.9%	-2.1	
LGVI	A.6.1	CT_80_dfloRE02	55.5	54.4	57.3	2.9	8.7	2.8%	12.5	
LGVI	Dp.6.2	CT_25_FC_2D_S2	55.5	54.4	59.0	4.6	7.4	28.9%	-0.4	
LGVI	FR.6.1	CT_35_mon0001	55.5	50.1	59.0	8.9	8.9	14.4%	-0.7	
LGVI	FR.6.1	CT_50_dij0001	55.5	50.1	56.6	6.5	7.5	12.0%	-1.0	
LGVI	FR.6.1	CT_54_dij0102	55.5	50.1	56.6	6.5	12.3	9.1%	-0.9	
LGVI	FR.6.1	CT_60_col0102	55.5	50.1	57.3	7.2	9.6	10.3%	-0.9	
LGVI	FR.6.1	CT_62_lus0102	55.5	50.1	59.0	8.9	7.6	7.9%	-0.5	
LGVI	Dp.6.2	CT_15_DS_STIP_S3	56.1	50.1	59.0	8.9	5.9	12.8%	-6.2	
LGVI	FR.6.1	CT_39_mon0102	56.1	50.1	59.0	8.9	5.9	5.7%	-0.7	
LGVI	FR.6.1	CT_42_mon0203	56.1	50.1	56.6	6.5	8.8	14.4%	-0.7	
LGVI	FR.6.1	CT_45_cle0102	56.1	50.1	56.6	6.5	11.2	11.6%	-1.0	
LGVI	FR.6.1	CT_48_cle0203	56.1	50.1	56.9	6.8	11.8	12.6%	-1.0	
LGVI	FR.6.1	CT_57_dij0203	56.1	50.1	56.6	6.5	11.4	12.7%	-1.0	MDAF.6.2
LGVI	A.6.1	CT_73_StLe	56.1	55.5	56.9	1.4	17.8	11.4%	-3.7	
LGVI	Dp.6.2	JF_10_DS_STIP_S3	56.6	50.1	57.3	7.2	4.4	10.9%	-2.8	
LGVI	Dp.6.2	JF_12_DS_STEM_S3	56.6	50.1	57.3	7.2	4.5	10.3%	-2.6	
LGVI	Dp.6.2	CT_29_DS_LE_S2	56.6	54.4	59.0	4.6	9.0	34.1%	-22.7	
LGVI	A.6.1	CT_77_dfloRE01	56.9	54.4	59.4	5.0	4.6	3.1%	9.8	
LGVI	A.6.1	CT_83_dfloMO02	56.9	54.4	59.0	4.6	10.6	3.5%	8.3	
LGVI	Dp.6.2	JF_21_DS_LE_S3	57.0	50.1	59.0	8.9	9.8	35.1%	-57.1	
LGVI	Dp.6.2	JF_6_DS_STIP_S2	57.3	56.2	59.0	2.8	11.5	24.0%	-3.7	
LGVI	Dp.6.2	JF_8_DS_STEM_S2	57.3	55.1	58.2	3.1	9.1	18.4%	-1.9	
LGVI	Dp.6.2	CT_24_DS_LE_S1	57.5	54.4	60.4	6.0	9.1	18.2%	-57.7	
LGVI	Dp.6.2	JF_2_DS_STIP_S1	58.2	56.2	59.4	3.2	7.3	19.2%	-3.8	
LGVI	Dp.6.2	JF_4_DS_STEM_S1	58.2	57.3	59.4	2.1	6.2	15.9%	-2.2	
LGVI	Dp.6.2	JF_18_DS_LE_S2	58.2	56.6	59.0	2.4	5.1	19.9%	-17.9	
LGVI	A.6.2	CT_69_NbBr	58.5	56.1	60.4	4.3	5.3	5.8%	0.5	
LGVI	A.6.2	JF_29_StLe	59.4	58.2	61.5	3.4	10.9	28.6%	-3.1	
LGVI	A 6.2	JD_17_NbBr	63.7	35.3	67.2	31.9	9.5	19.5%	0.6	
LGVI	FR.6.2	CT_64_NCC	63.5	61.5	65.1	3.6	6.2	14.5%	-0.3	MDAF.6.3
LGVI	Dp.6.3	CT_27_FC_3D_S2	64.5	62.2	66.5	4.3	7.7	29.3%	-0.2	
LGVI	Dp.6.3	CT_10_DS_STIP_S1	67.2	64.5	71.1	6.6	6.3	10.1%	-6.8	
LGVI	A.6.3	JF_27_NbBr	75.1	72.9	75.7	2.8	15.1	30.5%	0.8	none
LGVI	Dp.6.4	CT_30_FC_3D_S3	105.3	102.8	107.7	4.9	3.4	17.3%	0.3	none
LGVII	A7.1	JD_18_NbBr	68.3	67.5	74.2	6.7	5.9	11.2%	0.9	
LGVII	Dp7.1	JD_29_DS_STIP_S2	68.3	64.6	70.5	5.9	8.6	15.9%	-11.6	MDA.7.1
LGVII	Dp7.1	JD_32_DS_STEM_S2	68.3	67.5	68.9	1.3	17.7	35.0%	-15.6	
LGVII	A7.2	JF_30_StLe	81.2	78.7	91.4	12.7	6.2	14.6%	2.4	MDA.7.2
LGVII	Dp.7.2	JF_19_FC_3D_S3	93.0	82.1	95.7	13.6	3.2	13.9%	-0.2	
LGVII	A.7.3	JF_23_HT	99.0	98.6	99.3	0.7	13.8	26.0%	35.8	none
LGVII	A.7.3	JF_24_NbNo	99.3	98.6	100.9	2.3	6.6	19.3%	0.3	none
LGVII	Dp.7.3	JF_14_FC_2D_S1	102.1	99.3	105.8	6.5	5.1	17.9%	-0.3	*none*
LGVII	Dp.7.3	JF_16_FC_3D_S1	102.1	99.3	105.8	6.5	5.1	18.6%	-0.3	none
LGVII	Dp.7.3	JF_17_DS_LE_S1	102.1	99.3	105.8	6.5	4.3	18.4%	-56.7	none
