# Supplementary material for: Five Regions of the Pea Genome Co-Control Partial Resistance to D. pinodes, Tolerance to Frost, and Some Architectural or Phenological Traits
Source: Genes (Basel). 2023 Jul 4;14(7):1399. doi: 10.3390/genes14071399 (PMC10379203; doi:10.3390/genes14071399)
Supplement: Supplementary file 1 [file genes-14-01399-s001.zip › genes-2286623 - Table S3.pdf]

MDAF	Marker mapped near MDAF peak	SNP genetic position (cM)	SNP physical position (b)*	Putative corresponding gene and/or predicted protein function	Reference
MDAF.3.1	Le_1 / Le	152.0		Le ; giberellin 3beta hydroxylase	Lester et al, 1997
	PsCam037922_22979_691	152.0	chr5LG3:567367194..567367193	Protein SRG1 / gibberellin 3-oxidase 1	Tayeh et al 2015 / Table S9
	PsCam038309_23348_747	152.0	chr5LG3:566441966..566441965	damaged DNA binding;DNA-directed DNA polymerases	Tayeh et al 2015 / Table S9
	PsCam035617_20792_637	152.0	chr5LG3:566953569..566953568	Receptor-like protein kinase	Tayeh et al 2015 / Table S9
	PsCam053662_35502_3991	152.0	chr5LG3:568153271..568153270	Pleiotropic drug resistance protein	Tayeh et al 2015 / Table S9
	TE002G22	152.3		TE002G22 ; EST TE.002G22F011204 ; Mtr protein LURP-one-related 12 (LOC11420985), mRNA	Genoplante SNPEA (Unpublished)
	PsCam002150_1736_414	152.3	chr5LG3:570321158..570321157	unknown	Tayeh et al 2015 / Table S9
	PsCam043642_27634_214	152.3	chr5LG3:569700788..569700787	unknown	Tayeh et al 2015 / Table S9
MDAF.3.2	ELF3 / HR / ElF3_S-P192C	29.3		Hr/Elf3	Weller et al 2012
	eSSR253-143	29.3		Ps shoot apical meristem EST ; Genebank EX569215.1 ; Mtr AP2-like ethylene responsive transcription factor	Mohamadi (Unpublished)
	PsCam057866_38405_252	29.3	chr5LG3:74499484..74499483	Chaperone DnaJ-domain superfamily protein	Tayeh et al 2015 / Table S9
	PsCam006676_4957_2275	29.3	chr5LG3:89445038..89445037	Endo-1,4-beta-xylanase / glycosyl hydrolase family	Tayeh et al 2015 / Table S9
MDAF.5.1	AGL20a	68.8		AGAMOUS Like 20 ; SOC1 [Arabidopsis thaliana], flowering regulation	Hecht et al 2005
	PsCam044905_28648_1122	68.8	chr3LG5:218287591..218287590	Phospholipase D	Tayeh et al 2015 / Table S9
	PsCam044478_28344_1306	67.6	chr3LG5:216354077..216354076	(+)-neomenthol dehydrogenase	Tayeh et al 2015 / Table S9
MDAF.5.2.1	PsCam054307_35867_264	70.4	chr3LG5:225671077..225671076	putative UPF0481 protein At3g02645-like	Tayeh et al 2015 / Table S9
	PsCam006738_5010_613	71.1	chr3LG5:220265687..220265686	Transducin/WD40 repeat-like superfamily protein	Tayeh et al 2015 / Table S9
MDAF.5.2	PsCam000454_396_539	75.1	chr3LG5:235855203..235855202	Pentatricopeptide repeat (PPR) superfamily protein	Tayeh et al 2015 / Table S9
MDAF.5.2.2	Ps900368 (P5NI1F19_138)	78.1		P5NI1F19 ; EST 552NI.001F19F.040526(T7.A1) ; 552NI.001F19F 552NI Pisum sativum cDNA clone 552NI001F19, mRNA sequence ; Trifolium pratense abscisic acid receptor PYL4-like (LOC123889688), mRNA	Genoplante SNPEA (Unpublished)
	PsCam059063_39333_3417	78.1	chr3LG5:240640184..240640183	GDA2 protein / Kelch-like protein	Tayeh et al 2015 / Table S9
	PsCam003747_2870_251	78.2	chr3LG5:244520422..244520421	unknown	Tayeh et al 2015 / Table S9
	Ps900223 (TE002L23_83)	78.4		TE002L23 ; genebank CD860534.1 ; EST TE.002L23F.011204 ; AT3G10940 protein phosphatase-related [Arabidopsis thaliana]	Genoplante SNPEA (Unpublished)
	TE002L23	78.4		TE002L23 ; genebank CD860534.1 ; EST TE.002L23F.011204 ; AT3G10940 protein phosphatase-related [Arabidopsis thaliana]	Genoplante SNPEA (Unpublished)
	Ps900319 (BNI2O22_99)	78.4		BNI2O22 ; EST BACNI.002O22F.040527(T7.A1) ; BACNI.002O22F BACNI Pisum sativum cDNA clone BACNI002O22, mRNA sequence ; Trifolium pratense alcohol dehydrogenase-like 3 (LOC123888930), mRNA	Genoplante SNPEA (Unpublished)
MDAF.6.2	PsCam050834_33394_133	55.5	chr1LG6:165743845..165743844	Two-component response regulator ARR1	Beji et al 2020 / Table S3 - Tayeh et al 2015 / Table S9
	PsCam052343_34679_521	55.5	chr1LG6:162492168..162492167	Superoxide dismutase	Tayeh et al 2015 / Table S9
	PsCam006662_4945_2210	55.5	chr1LG6:151907653..151907652	YTH domain family protein	Tayeh et al 2015 / Table S9
	Ps000349	55.5		Chalcone and stilbene synthases	Duarte et al.2014 / Additional file 6_tableS2
	eSSR388-479	55.5		Genebank FG530657.1 ; Pisum sativum Shoot apical meristem ESTs Pisum sativum cDNA clone MERIP0005J23.B, mRNA sequence ; Medicago truncatula dehydrin HIRD11 (LOC25495841), mRNA	Mohamadi (Unpublished)
	PsCam022275_12527_210	56.1	chr1LG6:167098866..167098865	unknown	Beji et al 2020 / Table S3 - Tayeh et al 2015 / Table S9
	C20a_166 / C20a_455 / C20a_103 / Ps900384 / Ps900263 / Ps900345	56.6		CE07C20a ; EST CE.007C20F010727 ; Medicago truncatula hypersensitive-induced response protein 4 (LOC25497124), mRNA	Genoplante SNPEA (Unpublished)
	Ps900306 (CNE007E16F010727_402)	56.6		CNE.007E16F010727 ; Medicago truncatula chlorophyll a-b binding protein CP26, chloroplastic (LOC25496571), mRNA	Genoplante SNPEA (Unpublished)
	PsCam053556_35439_228	56.6	chr1LG6:166361064..166361063	unknown	Beji et al 2020 / Table S3 - Tayeh et al 2015 / Table S9
	PsCam057485_38078_2761	56.6	chr1LG6:167388674..167388673	Pyruvate kinase	Tayeh et al 2015 / Table S9
	PsCam023246_13111_1125	56.6	chr1LG6:167654791..167654790	BAHD acyltransferase DCR	Tayeh et al 2015 / Table S9
	PsCam048404_31138_2325	56.6	chr1LG6:170956086..170956085	glutathione reductase	Beji et al 2020 / Table S3 - Tayeh et al 2015 / Table S9
	PsCam037082_22189_1302	56.6	chr1LG6:181965322..181965321	SET domain-containing protein	Beji et al 2020 / Table S3 - Tayeh et al 2015 / Table S9
	PsCam037030_22140_221	56.6	chr1LG6:186962069..186962068	dehydration response element B1A	Tayeh et al 2015 / Table S9
	PsCam035398_20586_1094	56.6	chr1LG6:194261395..194261394	ABC transporter B family member	Beji et al 2020 / Table S3 - Tayeh et al 2015 / Table S9
	PsCam027142_15832_646	56.6	chr1LG6:202829085..202829084	Cytochrome P450	Tayeh et al 2015 / Table S9
	PsCam034572_19953_2034	56.6	chr1LG6:211986743..211986742	unknown	Tayeh et al 2015 / Table S9
	Ps001552	56.6		General substrate transporter; Sucrose/H+ symporter	Duarte et al.2014 / Additional file 6_tableS2
	Ps001561	56.6		Per1-like chr06_pseudomolecule	Duarte et al.2014 / Additional file 6_tableS2
	Cabb	56.9		Cabb, Chlorophyll binding protein	Tayeh et al 2013
	Ps900259 (NT6011_238)	56.9		NT6011 ; receptor-like protein kinase	Tayeh et al 2013
	PsCam009729_6514_1100	56.9	chr1LG6:211341522..211341521	Hypersensitive-induced response protein	Tayeh et al 2015 / Table S9
	PsCam036289_21437_838	56.9	scaffold01545:150063..150062	brassinosteroid receptor / LRR receptor-like serine/threonine-protein kinase GSO2-like	Tayeh et al 2015 / Table S9
	PsCam042568_26621_42	57.3	chr1LG6:213838599..213838598	ATP-dependent Clp protease	Beji et al 2020 / Table S3 - Tayeh et al 2015 / Table S9
	Ps001357	57.3		Kunitz inhibitor ST1-like	Duarte et al.2014 / Additional file 6_tableS2
(MDA.6.1)	PsCam035356_20546_778	34.7	chr1LG6:221502038..221502037	unknown	Beji et al 2020 / Table S3 - Tayeh et al 2015 / Table S9
MDAF.6.3	PsCam040925_25551_719	62.2	chr1LG6:257177181..257177180	VQ motif-containing protein	Tayeh et al 2015 / Table S9
	PsCam040301_25080_740	64.3	chr1LG6:263433113..263433112	RING finger protein	Tayeh et al 2015 / Table S9
	Ps900089 (TC43620-225)	64.5		TC43620 ; genebank AJ311624.1 ; Mtr germin-like protein	Hamon et al 2010

***https://urgi.versailles.inra.fr/jbrowse/gmod_jbrowse/**
