# Supplementary material for: Five Regions of the Pea Genome Co-Control Partial Resistance to D. pinodes, Tolerance to Frost, and Some Architectural or Phenological Traits
Source: Genes (Basel). 2023 Jul 4;14(7):1399. doi: 10.3390/genes14071399 (PMC10379203; doi:10.3390/genes14071399)
Supplement: Supplementary file 1 [file genes-14-01399-s001.zip › Supplementary File S1 - Pathogenicity tests.pdf]

genes MDPI

Additional File S1 - Pathogenicity tests

1. Material and Methods:

A set of 11 pea lines was used for pathogenicity tests (Table S4), including spring (Ballet, Terese, Torsdag) or winter (Kazar, FP) field pea cultivars or breeding lines, spring garden pea cultivars (Cameor, JI296), or fodder pea lines (Champagne, DP, Melrose, VavD265). Most cultivars and lines are fully described in Baranger et al [1]. JI296 (cv. Chemin Long, Vilmorin) is a dwarf garden pea cultivar with white flowers, conventional leaves, early flowering. FP (synonym CE101, [1]) is a dwarf dry breeding line with white flowers, conventional leaves. DP and Champagne are fodder pea lines with purple flowers, indeterminate growth habit, and conventional leaves. Terese is a dry pea line with white flowers and afila foliage. DP, FP, Champagne and Melrose are known to be partially resistant to *D.pinodes*, whereas JI296 and Terese are highly susceptible [2].

Table S4. End-use, foliage type (af: afila or Af: conventional), internode length (LE: long or le: short), sensitivity to photoperiod (HR: highly responsive or hr: not responsive) and susceptibility level to *D. pinodes* of the eleven pea genotypes assessed for partial resistance under controlled conditions (full description in Baranger et al. 2004).

Name	**End-use**	***Af* / *af***	***Le* / *le***	***Hr* / *hr***	**Susceptibility to *D.pinodes* (Onfroy et al. 2007)**
Ballet	Dry	*af*	-	*hr*	-
Cameor	Garden	*Af*	-	*hr*	-
Champagne	Fodder	*Af*	*Le*	*Hr*	Partially resistant
DP	Fodder	*Af*	*Le*	*Hr*	Partially resistant
FP	Dry	*Af*	-	*hr*	Partially resistant
JI296	Garden	*Af*	*le*	*hr*	Susceptible
Kazar	Dry	*Af*	-	*hr*	-
Melrose	Fodder	*Af*	-	*Hr*	Partially resistant
Terese	Dry	*af*	*le*	*hr*	Susceptible
Torsdag	Dry	*Af*	*Le*	-	-
Vav D265	Fodder	-	-	-	-

Six single monosporic strains of *D. pinodes* (Table S5) were confronted to a set of eleven pea genotypes including pea RILs parents (Table S4) for the pathogenicity test under controlled conditions. This allowed to identify three strains with contrasting aggressiveness that were subsequently used to screen the three RIL populations for their susceptibility level to *D. pinodes*. Isolation conditions of the strains are described in Onfroy et al [3].

Table S5. Year of isolation, country of origin and level of aggressiveness of the six *D. pinodes* strains used in pathogenicity tests under controlled conditions.

Name	Year of isolation	Country of origin	Aggressiveness	References
Mp 91 31 12	1991	France	+++	*Onfroy et al 1999*
Mp 94 01 3	1994	France	++	*Onfroy et al 1999*
Mp 94 22 2	1994	France	-	*Onfroy et al 1999*
Mp 96 Ca 5	1996	Canada	-	-
Mp 96 Ma 2	1996	Morocco	-	-
Mp 97 WVF 421	1997	Australia	+	-

2. Results

Table 0. threshold and not significant compared to strain and genotype individual main effects (data not shown). Differences in pathogenicity were therefore mainly due to differences in aggressiveness (Table S6).

Table S6. Main and interaction factor effects obtained from ANOVA on disease severity assessed through AUDPC on stipules and stems for the pathogenicity test carried on plantlets with a set of eleven pea genotypes and six *D.pinodes* strains

Effects	Degree of freedom	Sum of squares (SS)	p-value	Sum of squares (SS)	p-value
		DS_STIP		DS_STEM	
Genotype	10	16768.49	<.0001	8899.65	<.0001
Strain	5	4955.63	<.0001	2426.71	<.0001
Block	3	2247.62	<.0001	1322.53	<.0001
Strain x Genotype	50	810.36	0.0322	704.82	0.0568
Genotype x Block	30	363.26	0.3786	476.82	0.037
Strain x Block	15	2175.76	<.0001	1620.81	<.0001

Disease severities on stipules and on stems were highly correlated (r = 0.86) for all strains, and symptom development was always weaker on stems than on stipules.

Significant differences in disease severities separated the six strains individually on stipules and into four groups on stems (Table S7). Aggressiveness rankings between strains were similar for disease severity on stipules and on stems. Mean disease severity assessed as AUDPC ranged from 9.72 to 17.87 on stipules, and from 5.50 to 10.28 on stems depending on the strain across the set of eleven genotypes. From these results a subset of three strains (Mp 91 31 12, Mp 94 01 3 and Mp 97 WVF 421 further named S1, S2 and S3 respectively) with contrasting levels of decreasing aggressiveness was chosen to perform subsequent disease severity tests on RIL populations. Significant differences in susceptibility to *D.pinodes* were also observed within the set of eleven pea genotypes (Table S4, Table S5). Genotypes previously described as susceptible (JI296 and Terese) showed high values of AUDPC on stipules and on stems. In the same way, genotypes previously described as partially resistant (Champagne, DP, FP and Melrose) showed the lowest values of AUDPC on stipules and stems among the set. Among genotypes which had not been previously described for resistance, Ballet and VavD265 showed high susceptibility, whereas Kazar showed some partial resistance both on stipules and stems.

Table S7. Mean disease severity and rankings between strains using SNK grouping on stipules and stems.

Strain	N	DS_STIP mean	DS_STIP SNK grouping	DS_STEM mean	DS_STEM SNK grouping
**Mp 91 31 12**	144	17.87	A	10.28	A
Mp 94 22 2	139	16.06	B	10.77	A
**Mp 94 01 3**	153	15.08	C	9.25	B
Mp 96 Ma 2	152	13.22	D	7.19	C
**Mp 97 WVF 421**	148	10.53	E	6.47	C
Mp 96 Ca 5	150	9.72	F	5.50	D
**In bold : strains selected for controlled conditions experiments on RIL populations**					

References

1. Baranger A, Aubert G, Arnau G, Lainé AL, Deniot G, Potier J, Weinachter C, Lejeune-Hénaut I, Lallemand J, Burstin J: **Genetic diversity within *Pisum sativum* using protein- and PCR-based markers**. *Theor Appl Genet* 2004, **108**(7):1309-1321.

2. Onfroy C, Baranger A, Tivoli B: **Biotic factors affecting the expression of partial resistance in pea to ascochyta blight in a detached stipule assay**. In: *Ascochyta blights of grain legumes.* Springer; 2007: 13-27.
3. Onfroy C, Tivoli B, Corbiere R, Bouznad Z: **Cultural, molecular and pathogenic variability of Mycosphaerella pinodes and Phoma medicaginis var. pinodella isolates from dried pea (Pisum sativum) in France**. *Plant Pathology (United Kingdom)* 1999.
