# Supplementary material for: Five Regions of the Pea Genome Co-Control Partial Resistance to D. pinodes, Tolerance to Frost, and Some Architectural or Phenological Traits
Source: Genes (Basel). 2023 Jul 4;14(7):1399. doi: 10.3390/genes14071399 (PMC10379203; doi:10.3390/genes14071399)
Supplement: Supplementary file 1 [file genes-14-01399-s001.zip › genes-2286623 - Figure S2.pdf]

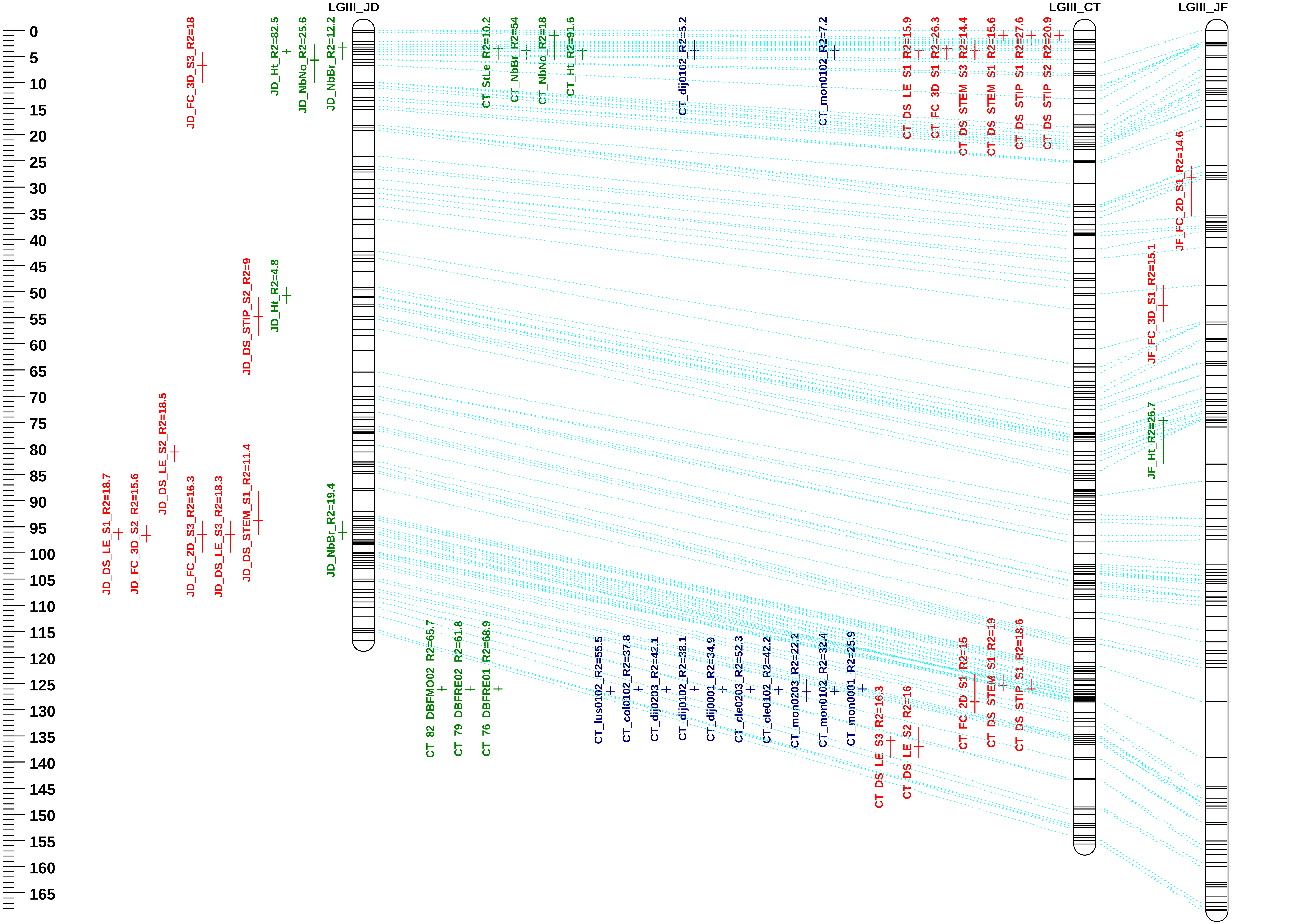

LGIII_JD
LGIII_CT
LGIII_JF
JD_FC_3D_S3_R2=18
JD_Ht_R2=82.5
JD_NbNo_R2=25.6
JD_NbBr_R2=12.2
JD_DS_STIP_S2_R2=9
JD_Ht_R2=4.8
JD_DS_LE_S2_R2=18.5
JD_DS_LE_S1_R2=18.7
JD_FC_3D_S2_R2=15.6
JD_FC_2D_S3_R2=16.3
JD_DS_LE_S3_R2=18.3
JD_DS_STEM_S1_R2=11.4
JD_NbBr_R2=19.4
CT_StLe_R2=10.2
CT_NbBr_R2=54
CT_NbNo_R2=18
CT_Ht_R2=91.6
CT_dij0102_R2=5.2
CT_mon0102_R2=7.2
CT_DS_LE_S1_R2=15.9
CT_FC_3D_S1_R2=26.3
CT_DS_STEM_S3_R2=14.4
CT_DS_STEM_S1_R2=15.6
CT_DS_STIP_S1_R2=27.6
CT_DS_STIP_S2_R2=20.9
CT_82_DBFMO02_R2=65.7
CT_79_DBFRE02_R2=61.8
CT_76_DBFRE01_R2=68.9
CT_lus0102_R2=55.5
CT_col0102_R2=37.8
CT_dij0203_R2=42.1
CT_dij0102_R2=38.1
CT_dij0001_R2=34.9
CT_cle0203_R2=52.3
CT_cle0102_R2=42.2
CT_mon0203_R2=22.2
CT_mon0102_R2=32.4
CT_mon0001_R2=25.9
CT_DS_LE_S3_R2=16.3
CT_DS_LE_S2_R2=16
CT_FC_2D_S1_R2=15
CT_DS_STEM_S1_R2=19
CT_DS_STIP_S1_R2=18.6
JF_FC_2D_S1_R2=14.6
JF_FC_3D_S1_R2=15.1
JF_Ht_R2=26.7

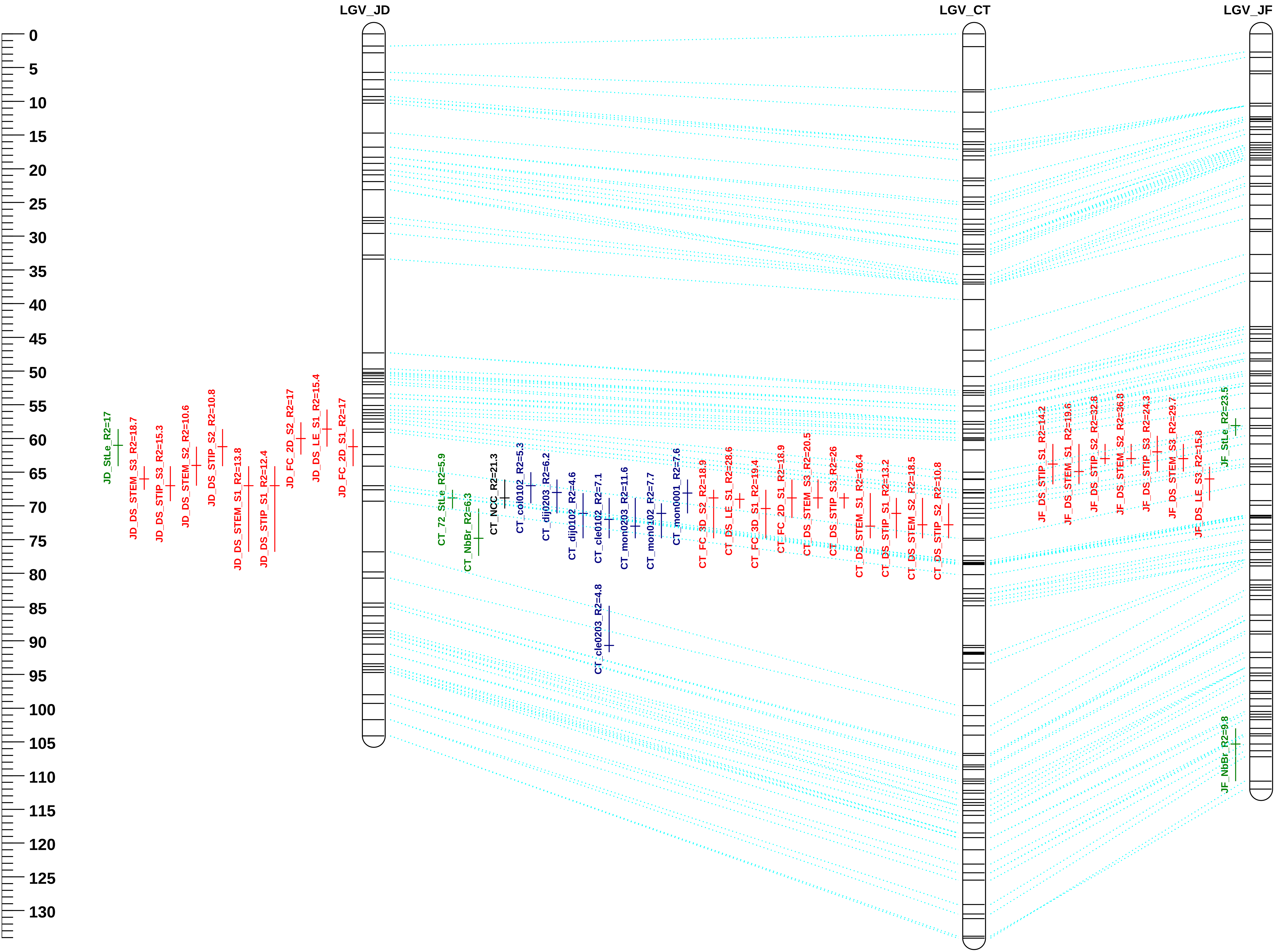

LGV_JD
LGV_CT
LGV_JF
0
5
10
15
20
25
30
35
40
45
50
55
60
65
70
75
80
85
90
95
100
105
110
115
120
125
130
JD_StLe_R2=17
JD_DS_STEM_S3_R2=18.7
JD_DS_STIP_S3_R2=15.3
JD_DS_STEM_S2_R2=10.6
JD_DS_STIP_S2_R2=10.8
JD_DS_STEM_S1_R2=13.8
JD_DS_STIP_S1_R2=12.4
JD_FC_2D_S2_R2=17
JD_DS_LE_S1_R2=15.4
JD_FC_2D_S1_R2=17
CT_72_StLe_R2=5.9
CT_NbBr_R2=6.3
CT_NCC_R2=21.3
CT_col0102_R2=5.3
CT_dij0203_R2=6.2
CT_dij0102_R2=4.6
CT_cle0102_R2=7.1
CT_cle0203_R2=4.8
CT_mon0203_R2=11.6
CT_mon0102_R2=7.7
CT_mon0001_R2=7.6
CT_FC_3D_S2_R2=18.9
CT_DS_LE_S1_R2=28.6
CT_FC_3D_S1_R2=19.4
CT_FC_2D_S1_R2=18.9
CT_DS_STEM_S3_R2=20.5
CT_DS_STIP_S3_R2=26
CT_DS_STEM_S1_R2=16.4
CT_DS_STIP_S1_R2=13.2
CT_DS_STEM_S2_R2=18.5
CT_DS_STIP_S2_R2=10.8
JF_DS_STIP_S1_R2=14.2
JF_DS_STEM_S1_R2=19.6
JF_DS_STIP_S2_R2=32.8
JF_DS_STEM_S2_R2=36.8
JF_DS_STIP_S3_R2=24.3
JF_DS_STEM_S3_R2=29.7
JF_DS_LE_S3_R2=15.8
JF_StLe_R2=23.5
JF_NbBr_R2=9.8

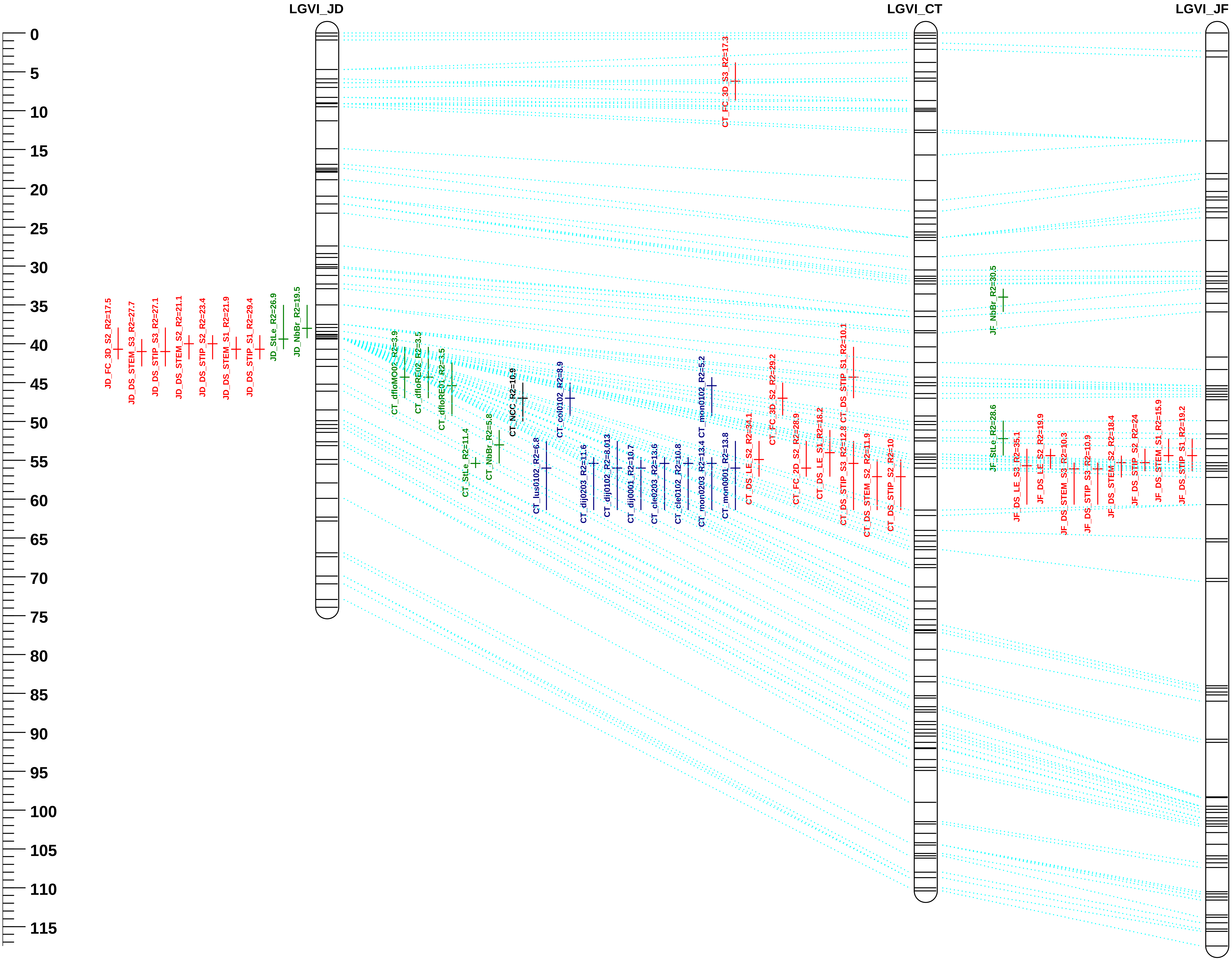
LGVI_JD
LGVI_CT
LGVI_JF
0
5
10
15
20
25
30
35
40
45
50
55
60
65
70
75
80
85
90
95
100
105
110
115
JD_FC_3D_S2_R2=17.5
JD_DS_STEM_S3_R2=27.7
JD_DS_STIP_S3_R2=27.1
JD_DS_STEM_S2_R2=21.1
JD_DS_STIP_S2_R2=23.4
JD_DS_STEM_S1_R2=21.9
JD_DS_STIP_S1_R2=29.4
JD_StLe_R2=26.9
JD_NbBr_R2=19.5
CT_dfloMO02_R2=3.9
CT_dfloRE02_R2=3.5
CT_dfloRE01_R2=3.5
CT_StLe_R2=11.4
CT_NbBr_R2=5.8
CT_NCC_R2=10.9
CT_lus0102_R2=6.8
CT_col0102_R2=8.9
CT_dij0203_R2=11.6
CT_dij0102_R2=8.013
CT_dij0001_R2=10.7
CT_cle0203_R2=13.6
CT_cle0102_R2=10.8
CT_mon0203_R2=13.4
CT_mon0102_R2=5.2
CT_mon0001_R2=13.8
CT_DS_LE_S2_R2=34.1
CT_FC_3D_S3_R2=17.3
CT_FC_3D_S2_R2=29.2
CT_FC_2D_S2_R2=28.9
CT_DS_LE_S1_R2=18.2
CT_DS_STIP_S3_R2=12.8
CT_DS_STIP_S1_R2=10.1
CT_DS_STEM_S2_R2=11.9
CT_DS_STIP_S2_R2=10
JF_NbBr_R2=30.5
JF_StLe_R2=28.6
JF_DS_LE_S3_R2=35.1
JF_DS_LE_S2_R2=19.9
JF_DS_STEM_S3_R2=10.3
JF_DS_STIP_S3_R2=10.9
JF_DS_STEM_S2_R2=18.4
JF_DS_STIP_S2_R2=24
JF_DS_STEM_S1_R2=15.9
JF_DS_STIP_S1_R2=19.2

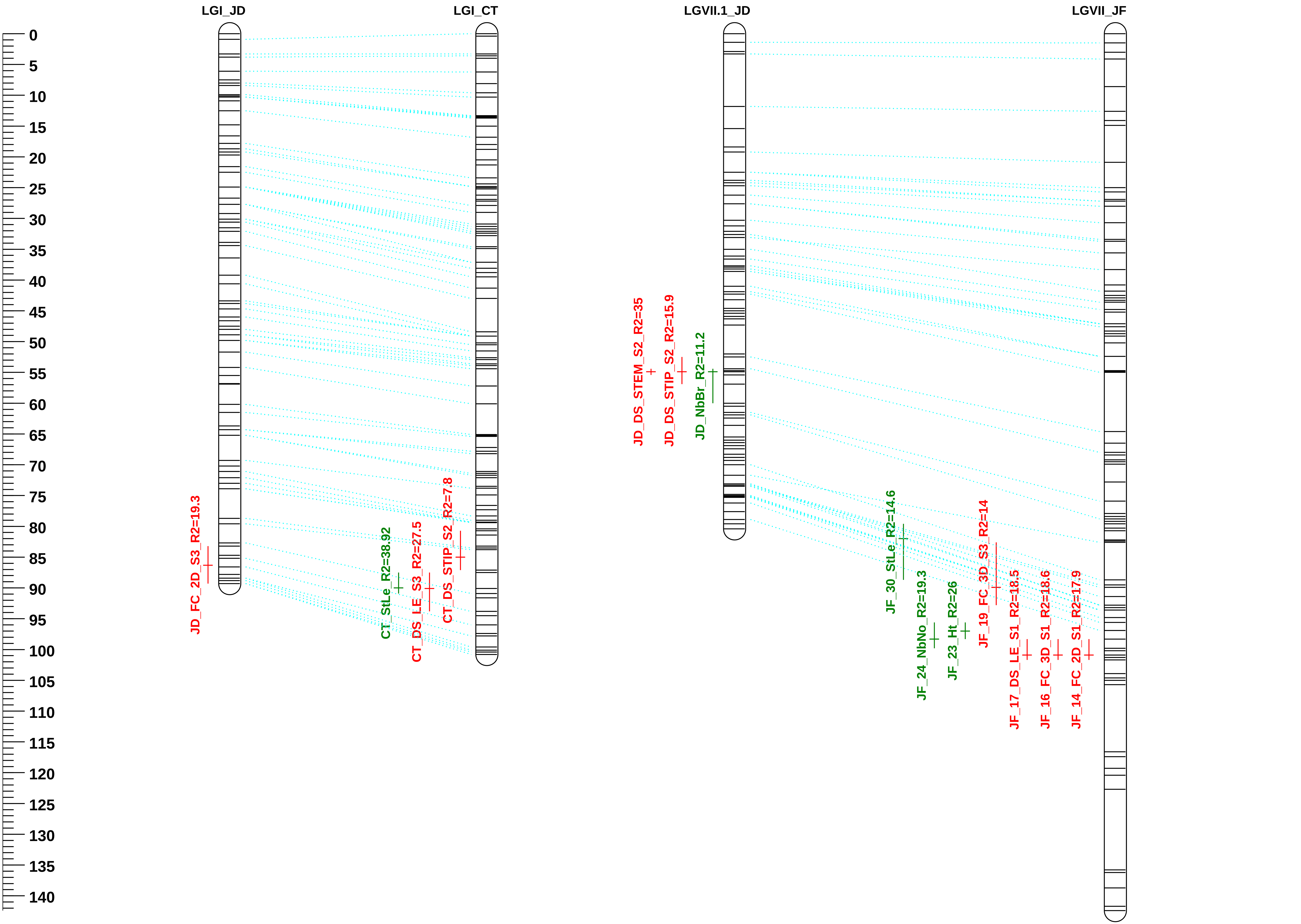
LGI_JD
LGI_CT
LGVII.1_JD
LGVII_JF
0
5
10
15
20
25
30
35
40
45
50
55
60
65
70
75
80
85
90
95
100
105
110
115
120
125
130
135
140
JD_FC_2D_S3_R2=19.3
CT_StLe_R2=38.92
CT_DS_LE_S3_R2=27.5
CT_DS_STIP_S2_R2=7.8
JD_DS_STEM_S2_R2=35
JD_DS_STIP_S2_R2=15.9
JD_NbBr_R2=11.2
JF_30_StLe_R2=14.6
JF_24_NbNo_R2=19.3
JF_23_Ht_R2=26
JF_19_FC_3D_S3_R2=14
JF_17_DS_LE_S1_R2=18.5
JF_16_FC_3D_S1_R2=18.6
JF_14_FC_2D_S1_R2=17.9
