# Supplementary material for: Five Regions of the Pea Genome Co-Control Partial Resistance to D. pinodes, Tolerance to Frost, and Some Architectural or Phenological Traits
Source: Genes (Basel). 2023 Jul 4;14(7):1399. doi: 10.3390/genes14071399 (PMC10379203; doi:10.3390/genes14071399)

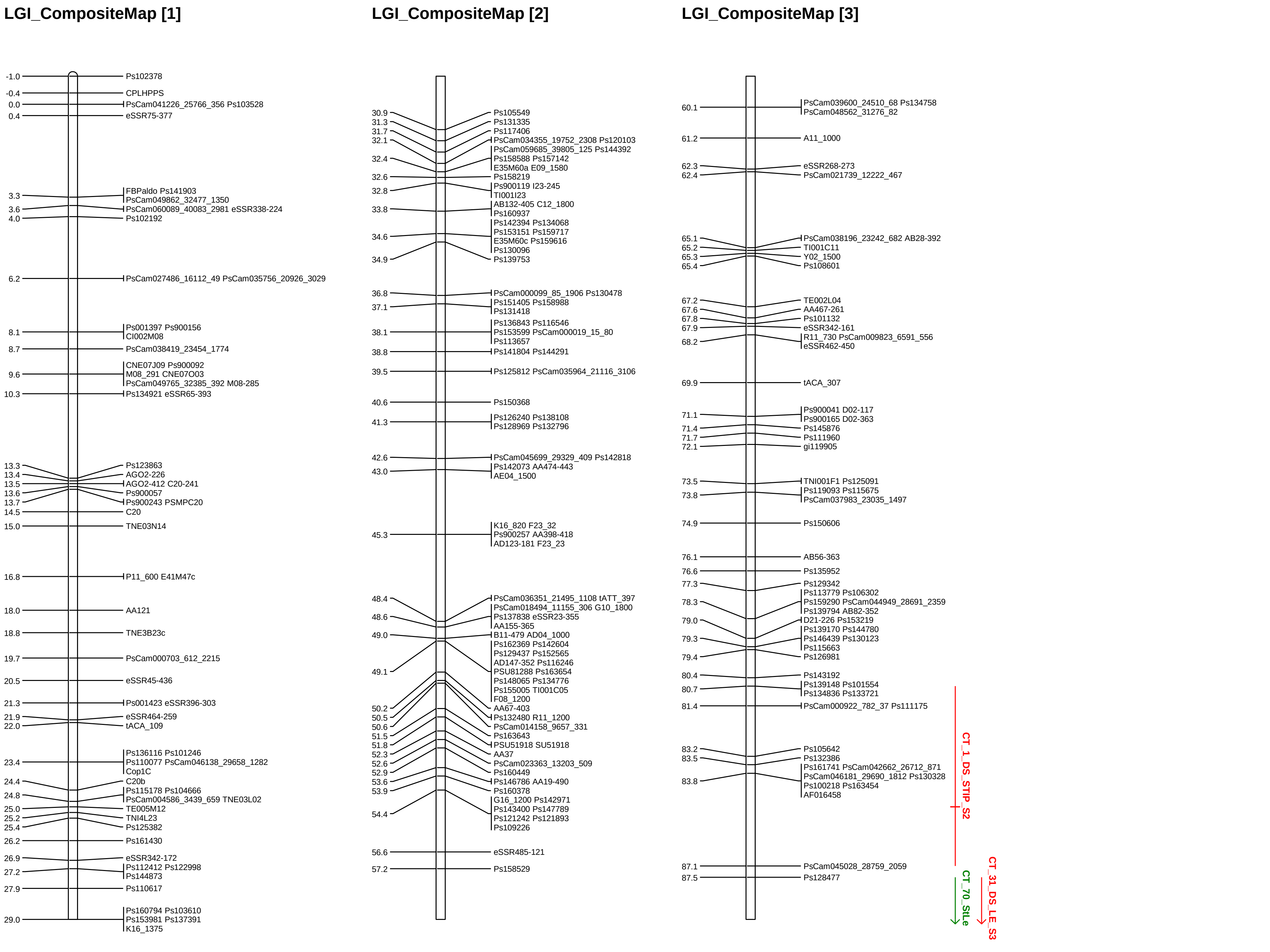

LGI_CompositeMap [1]
-1.0 Ps102378
-0.4 CPLHPPS
0.0 PsCam041226_25766_356 Ps103528
0.4 eSSR75-377
3.3 FBPaldo Ps141903
PsCam049862_32477_1350
3.6 PsCam060089_40083_2981 eSSR338-224
4.0 Ps102192
6.2 PsCam027486_16112_49 PsCam035756_20926_3029
8.1 Ps001397 Ps900156
CI002M08
8.7 PsCam038419_23454_1774
9.6 CNE07J09 Ps900092
M08_291 CNE07O03
PsCam049765_32385_392 M08-285
10.3 Ps134921 eSSR65-393
13.3 Ps123863
13.4 AGO2-226
13.5 AGO2-412 C20-241
13.6 Ps900057
13.7 Ps900243 PSMPC20
14.5 C20
15.0 TNE03N14
16.8 P11_600 E41M47c
18.0 AA121
18.8 TNE3B23c
19.7 PsCam000703_612_2215
20.5 eSSR45-436
21.3 Ps001423 eSSR396-303
21.9 eSSR464-259
22.0 tACA_109
23.4 Ps136116 Ps101246
Ps110077 PsCam046138_29658_1282
Cop1C
24.4 C20b
24.8 Ps115178 Ps104666
PsCam004586_3439_659 TNE03L02
25.0 TE005M12
25.2 TNI4L23
25.4 Ps125382
26.2 Ps161430
26.9 eSSR342-172
27.2 Ps112412 Ps122998
Ps144873
27.9 Ps110617
29.0 Ps160794 Ps103610
Ps153981 Ps137391
K16_1375
LGI_CompositeMap [2]
30.9 Ps105549
31.3 Ps131335
31.7 Ps117406
32.1 PsCam034355_19752_2308 Ps120103
32.4 PsCam059685_39805_125 Ps144392
Ps158588 Ps157142
E35M60a E09_1580
32.6 Ps158219
32.8 Ps900119 I23-245
TI001I23
33.8 AB132-405 C12_1800
Ps160937
34.6 Ps142394 Ps134068
Ps153151 Ps159717
E35M60c Ps159616
Ps130096
34.9 Ps139753
36.8 PsCam000099_85_1906 Ps130478
37.1 Ps151405 Ps158988
Ps131418
38.1 Ps136843 Ps116546
Ps153599 PsCam000019_15_80
Ps113657
38.8 Ps141804 Ps144291
39.5 Ps125812 PsCam035964_21116_3106
40.6 Ps150368
41.3 Ps126240 Ps138108
Ps128969 Ps132796
42.6 PsCam045699_29329_409 Ps142818
43.0 Ps142073 AA474-443
AE04_1500
45.3 K16_820 F23_32
Ps900257 AA398-418
AD123-181 F23_23
48.4 PsCam036351_21495_1108 tATT_397
48.6 PsCam018494_11155_306 G10_1800
Ps137838 eSSR23-355
AA155-365
49.0 B11-479 AD04_1000
49.1 Ps162369 Ps142604
Ps129437 Ps152565
AD147-352 Ps116246
PSU81288 Ps163654
Ps148065 Ps134776
Ps155005 TI001C05
F08_1200
50.2 AA67-403
50.5 Ps132480 R11_1200
50.6 PsCam014158_9657_331
51.5 Ps163643
51.8 PSU51918 SU51918
52.3 AA37
52.6 PsCam023363_13203_509
52.9 Ps160449
53.6 Ps146786 AA19-490
53.9 Ps160378
54.4 G16_1200 Ps142971
Ps143400 Ps147789
Ps121242 Ps121893
Ps109226
56.6 eSSR485-121
57.2 Ps158529
LGI_CompositeMap [3]
60.1 PsCam039600_24510_68 Ps134758
PsCam048562_31276_82
61.2 A11_1000
62.3 eSSR268-273
62.4 PsCam021739_12222_467
65.1 PsCam038196_23242_682 AB28-392
65.2 TI001C11
65.3 Y02_1500
65.4 Ps108601
67.2 TE002L04
67.6 AA467-261
67.8 Ps101132
67.9 eSSR342-161
68.2 R11_730 PsCam009823_6591_556
eSSR462-450
69.9 tACA_307
71.1 Ps900041 D02-117
Ps900165 D02-363
71.4 Ps145876
71.7 Ps111960
72.1 gi119905
73.5 TNI001F1 Ps125091
73.8 Ps119093 Ps115675
PsCam037983_23035_1497
74.9 Ps150606
76.1 AB56-363
76.6 Ps135952
77.3 Ps129342
78.3 Ps113779 Ps106302
Ps159290 PsCam044949_28691_2359
Ps139794 AB82-352
79.0 D21-226 Ps153219
79.3 Ps139170 Ps144780
Ps146439 Ps130123
Ps115663
79.4 Ps126981
80.4 Ps143192
80.7 Ps139148 Ps101554
Ps134836 Ps133721
81.4 PsCam000922_782_37 Ps111175
83.2 Ps105642
83.5 Ps132386
83.8 Ps161741 PsCam042662_26712_871
PsCam046181_29690_1812 Ps130328
Ps100218 Ps163454
AF016458
87.1 PsCam045028_28759_2059
87.5 Ps128477
CT_1_DS_STIP_S2
CT_70_StLe
CT_31_DS_LE_S3

LGI_CompositeMap [4]

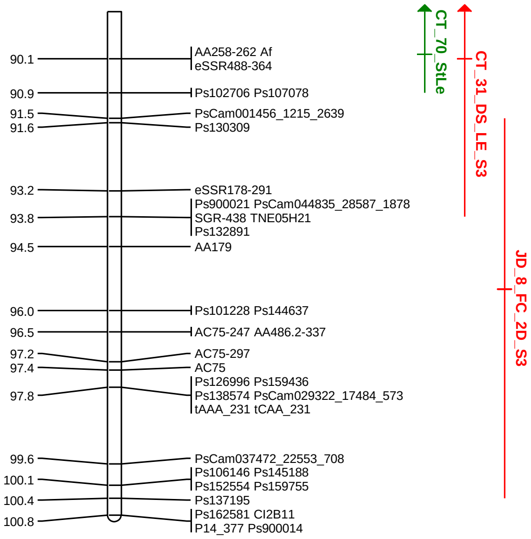

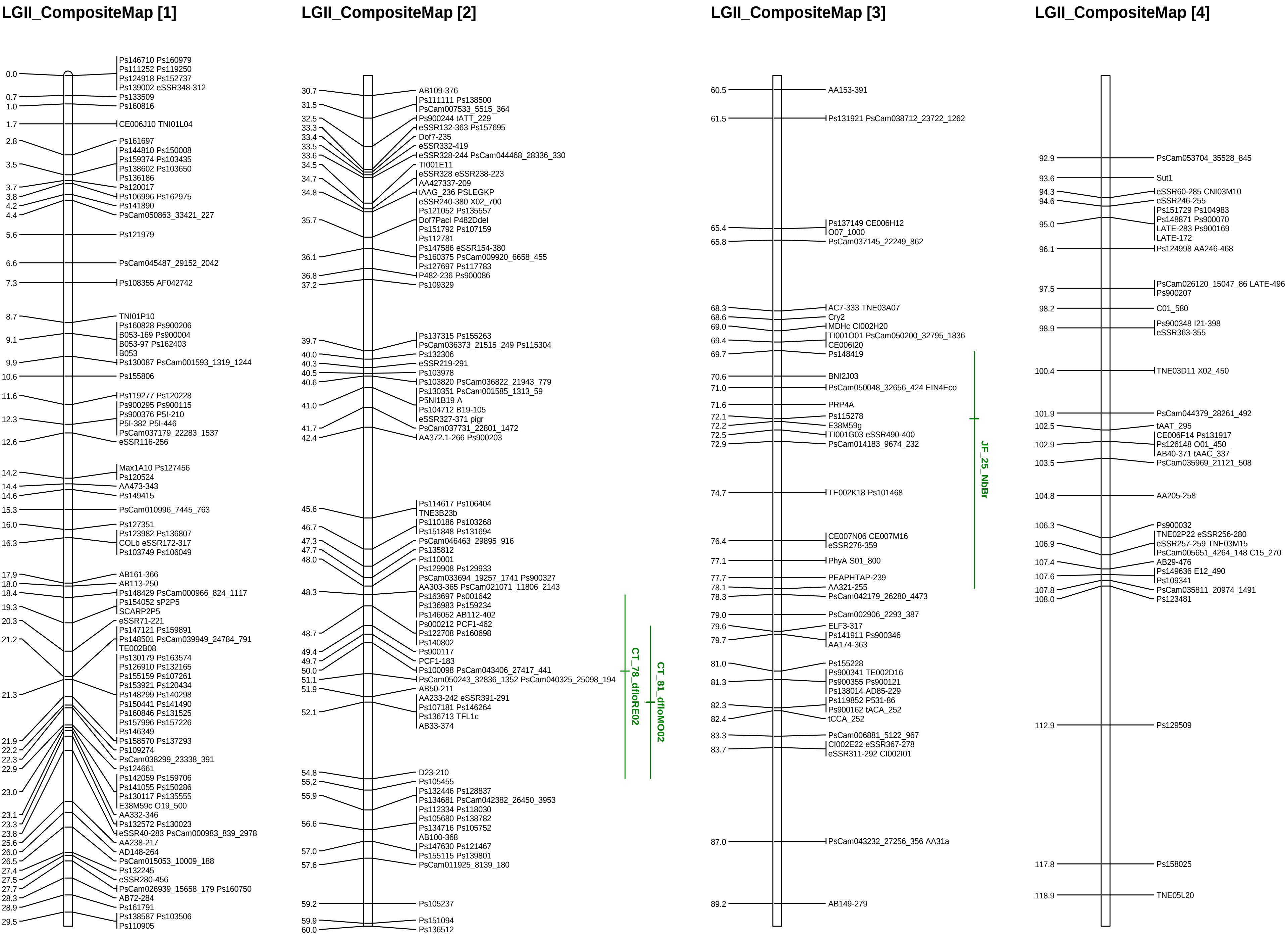
LGII_CompositeMap [1]
0.0 Ps146710 Ps160979
Ps111252 Ps119250
Ps124918 Ps152737
Ps139002 eSSR348-312
0.7 Ps133509
1.0 Ps160816
1.7 CE006J10 TNI01L04
2.8 Ps161697
3.5 Ps144810 Ps150008
Ps159374 Ps103435
Ps138602 Ps103650
Ps136186
3.7 Ps120017
3.8 Ps106996 Ps162975
4.2 Ps141890
4.4 PsCam050863_33421_227
5.6 Ps121979
6.6 PsCam045487_29152_2042
7.3 Ps108355 AF042742
8.7 TNI01P10
9.1 Ps160828 Ps900206
B053-169 Ps900004
B053-97 Ps162403
B053
9.9 Ps130087 PsCam001593_1319_1244
10.6 Ps155806
11.6 Ps119277 Ps120228
12.3 Ps900295 Ps900115
Ps900376 P5I-210
P5I-382 P5I-446
PsCam037179_22283_1537
12.6 eSSR116-256
14.2 Max1A10 Ps127456
Ps120524
14.4 AA473-343
14.6 Ps149415
15.3 PsCam010996_7445_763
16.0 Ps127351
16.3 Ps123982 Ps136807
COLb eSSR172-317
Ps103749 Ps106049
17.9 AB161-366
18.0 AB113-250
18.4 Ps148429 PsCam000966_824_1117
19.3 Ps154052 sP2P5
SCARP2P5
20.3 eSSR71-221
21.2 Ps147121 Ps159891
Ps148501 PsCam039949_24784_791
TE002B08
21.3 Ps130179 Ps163574
Ps126910 Ps132165
Ps155159 Ps107261
Ps153921 Ps120434
Ps148299 Ps140298
Ps150441 Ps141490
Ps160846 Ps131525
Ps157996 Ps157226
Ps146349
21.9 Ps158570 Ps137293
22.2 Ps109274
22.3 PsCam038299_23338_391
22.9 Ps124661
23.0 Ps142059 Ps159706
Ps141055 Ps150286
Ps130117 Ps135555
E38M59c O19_500
23.1 AA332-346
23.3 Ps132572 Ps130023
23.8 eSSR40-283 PsCam000983_839_2978
25.6 AA238-217
26.0 AD148-264
26.5 PsCam015053_10009_188
27.4 Ps132245
27.5 eSSR280-456
27.7 PsCam026939_15658_179 Ps160750
28.3 AB72-284
28.9 Ps161791
29.5 Ps138587 Ps103506
Ps110905
LGII_CompositeMap [2]
30.7 AB109-376
31.5 Ps111111 Ps138500
PsCam007533_5515_364
32.5 Ps900244 tATT_229
33.3 eSSR132-363 Ps157695
33.4 Dof7-235
33.5 eSSR332-419
33.6 eSSR328-244 PsCam044468_28336_330
34.5 TI001E11
34.7 eSSR328 eSSR238-223
AA427337-209
34.8 tAAG_236 PSLEGKP
35.7 eSSR240-380 X02_700
Ps121052 Ps135557
Dof7PacI P482DdeI
Ps151792 Ps107159
Ps112781
36.1 Ps147586 eSSR154-380
Ps160375 PsCam009920_6658_455
Ps127697 Ps117783
36.8 P482-236 Ps900086
37.2 Ps109329
39.7 Ps137315 Ps155263
PsCam036373_21515_249 Ps115304
40.0 Ps132306
40.3 eSSR219-291
40.5 Ps103978
40.6 Ps103820 PsCam036822_21943_779
41.0 Ps130351 PsCam001585_1313_59
P5NI1B19 A
Ps104712 B19-105
eSSR327-371 pigr
41.7 PsCam037731_22801_1472
42.4 AA372.1-266 Ps900203
45.6 Ps114617 Ps106404
TNE3B23b
46.7 Ps110186 Ps103268
Ps151848 Ps131694
47.3 PsCam046463_29895_916
47.7 Ps135812
48.0 Ps110001
48.3 Ps129908 Ps129933
PsCam033694_19257_1741 Ps900327
AA303-365 PsCam021071_11806_2143
Ps163697 Ps001642
Ps136983 Ps159234
Ps146052 AB112-402
48.7 Ps000212 PCF1-462
Ps122708 Ps160698
Ps140802
49.4 Ps900117
49.7 PCF1-183
50.0 Ps100098 PsCam043406_27417_441
51.1 PsCam050243_32836_1352 PsCam040325_25098_194
51.9 AB50-211
52.1 AA233-242 eSSR391-291
Ps107181 Ps146264
Ps136713 TFL1c
AB33-374
54.8 D23-210
55.2 Ps105455
55.9 Ps132446 Ps128837
Ps134681 PsCam042382_26450_3953
56.6 Ps112334 Ps118030
Ps105680 Ps138782
Ps134716 Ps105752
AB100-368
57.0 Ps147630 Ps121467
Ps155115 Ps139801
57.6 PsCam011925_8139_180
59.2 Ps105237
59.9 Ps151094
60.0 Ps136512
CT_78_dfloRE02
CT_81_dfloMO02
LGII_CompositeMap [3]
60.5 AA153-391
61.5 Ps131921 PsCam038712_23722_1262
65.4 Ps137149 CE006H12
O07_1000
65.8 PsCam037145_22249_862
68.3 AC7-333 TNE03A07
68.6 Cry2
69.0 MDHc CI002H20
69.4 TI001O01 PsCam050200_32795_1836
CE006I20
69.7 Ps148419
70.6 BNI2J03
71.0 PsCam050048_32656_424 EIN4Eco
71.6 PRP4A
72.1 Ps115278
72.2 E38M59g
72.5 TI001G03 eSSR490-400
72.9 PsCam014183_9674_232
74.7 TE002K18 Ps101468
76.4 CE007N06 CE007M16
eSSR278-359
77.1 PhyA S01_800
77.7 PEAPHTAP-239
78.1 AA321-255
78.3 PsCam042179_26280_4473
79.0 PsCam002906_2293_387
79.6 ELF3-317
79.7 Ps141911 Ps900346
AA174-363
81.0 Ps155228
81.3 Ps900341 TE002D16
Ps900355 Ps900121
Ps138014 AD85-229
82.3 Ps119852 P531-86
Ps900162 tACA_252
82.4 tCCA_252
83.3 PsCam006881_5122_967
83.7 CI002E22 eSSR367-278
eSSR311-292 CI002I01
87.0 PsCam043232_27256_356 AA31a
89.2 AB149-279
JF_25_NbBr
LGII_CompositeMap [4]
92.9 PsCam053704_35528_845
93.6 Sut1
94.3 eSSR60-285 CNI03M10
94.6 eSSR246-255
95.0 Ps151729 Ps104983
Ps148871 Ps900070
LATE-283 Ps900169
LATE-172
96.1 Ps124998 AA246-468
97.5 PsCam026120_15047_86 LATE-496
Ps900207
98.2 C01_580
98.9 Ps900348 I21-398
eSSR363-355
100.4 TNE03D11 X02_450
101.9 PsCam044379_28261_492
102.5 tAAT_295
102.9 CE006F14 Ps131917
Ps126148 O01_450
AB40-371 tAAC_337
103.5 PsCam035969_21121_508
104.8 AA205-258
106.3 Ps900032
106.9 TNE02P22 eSSR256-280
eSSR257-259 TNE03M15
PsCam005651_4264_148 C15_270
107.4 AB29-476
107.6 Ps149636 E12_490
Ps109341
107.8 PsCam035811_20974_1491
108.0 Ps123481
112.9 Ps129509
117.8 Ps158025
118.9 TNE05L20

LGII_CompositeMap [5]

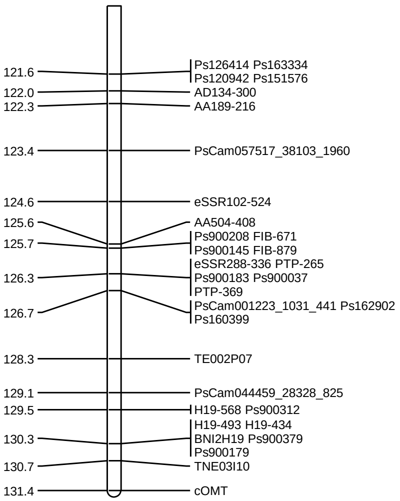

LGIII_CompositeMap [1]

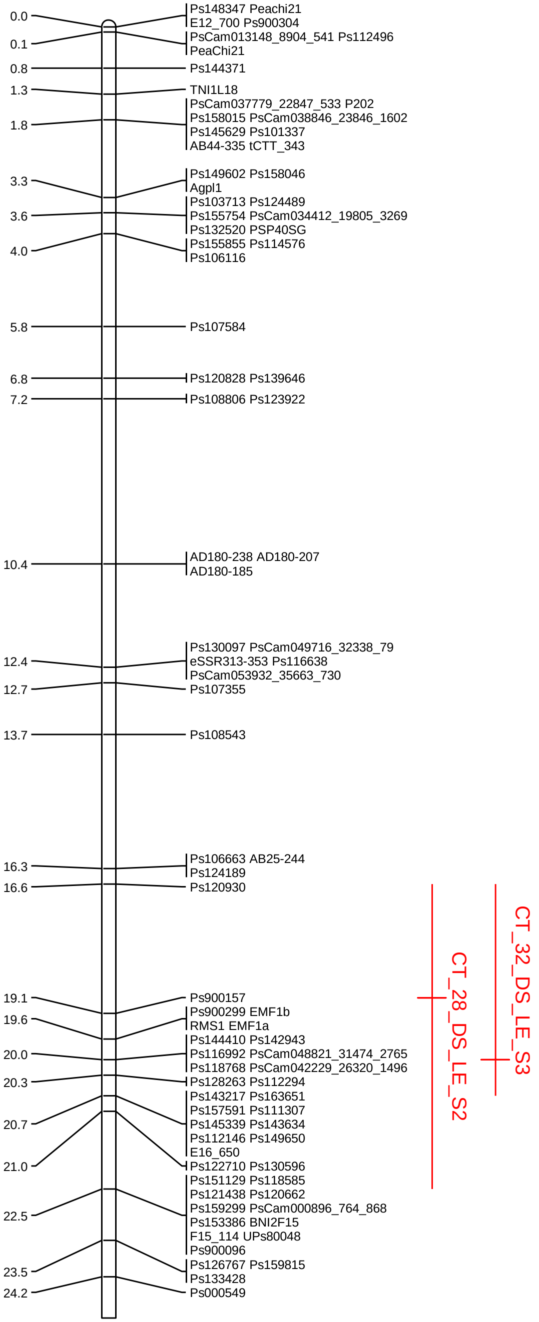

LGIII_CompositeMap [2]

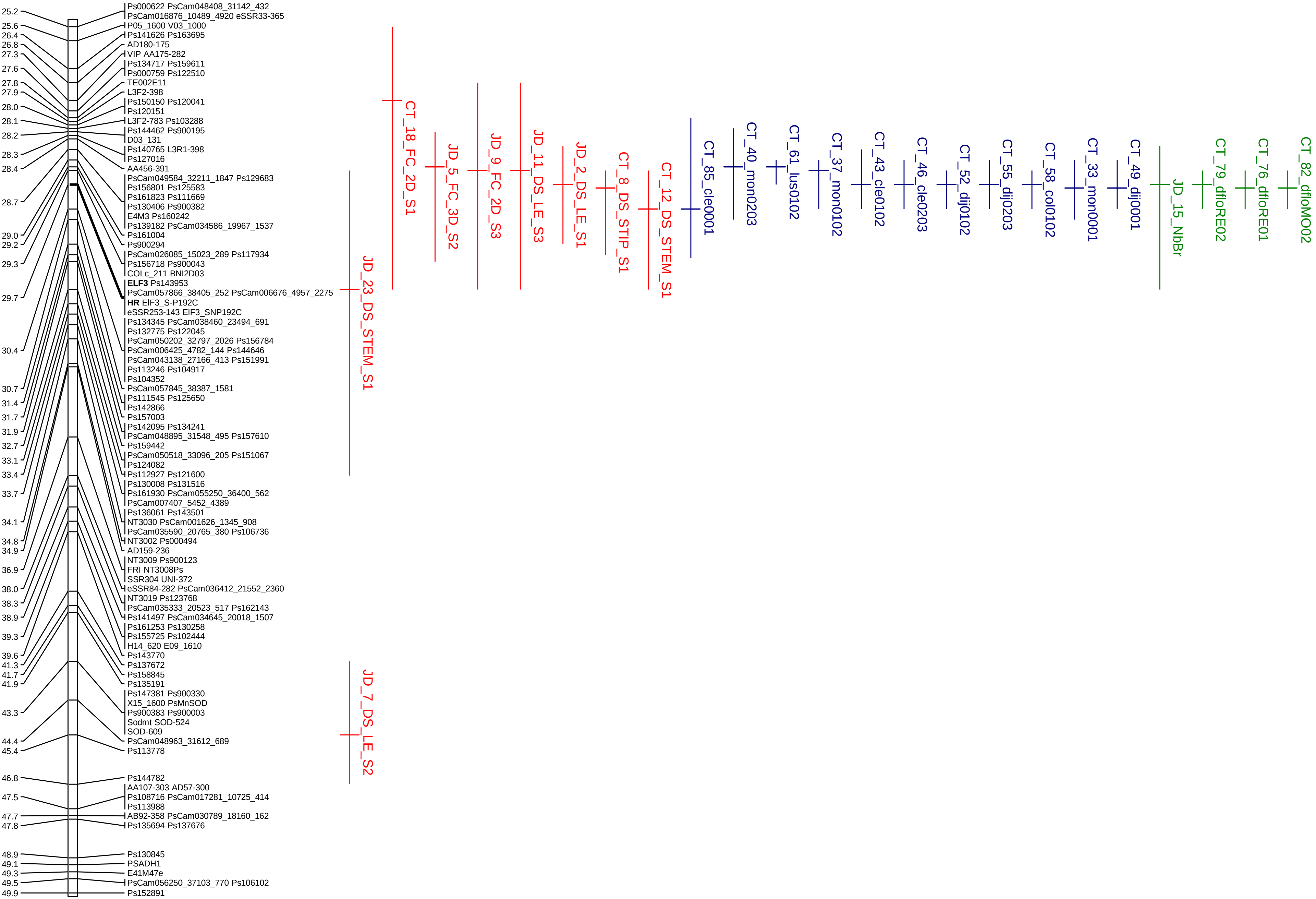

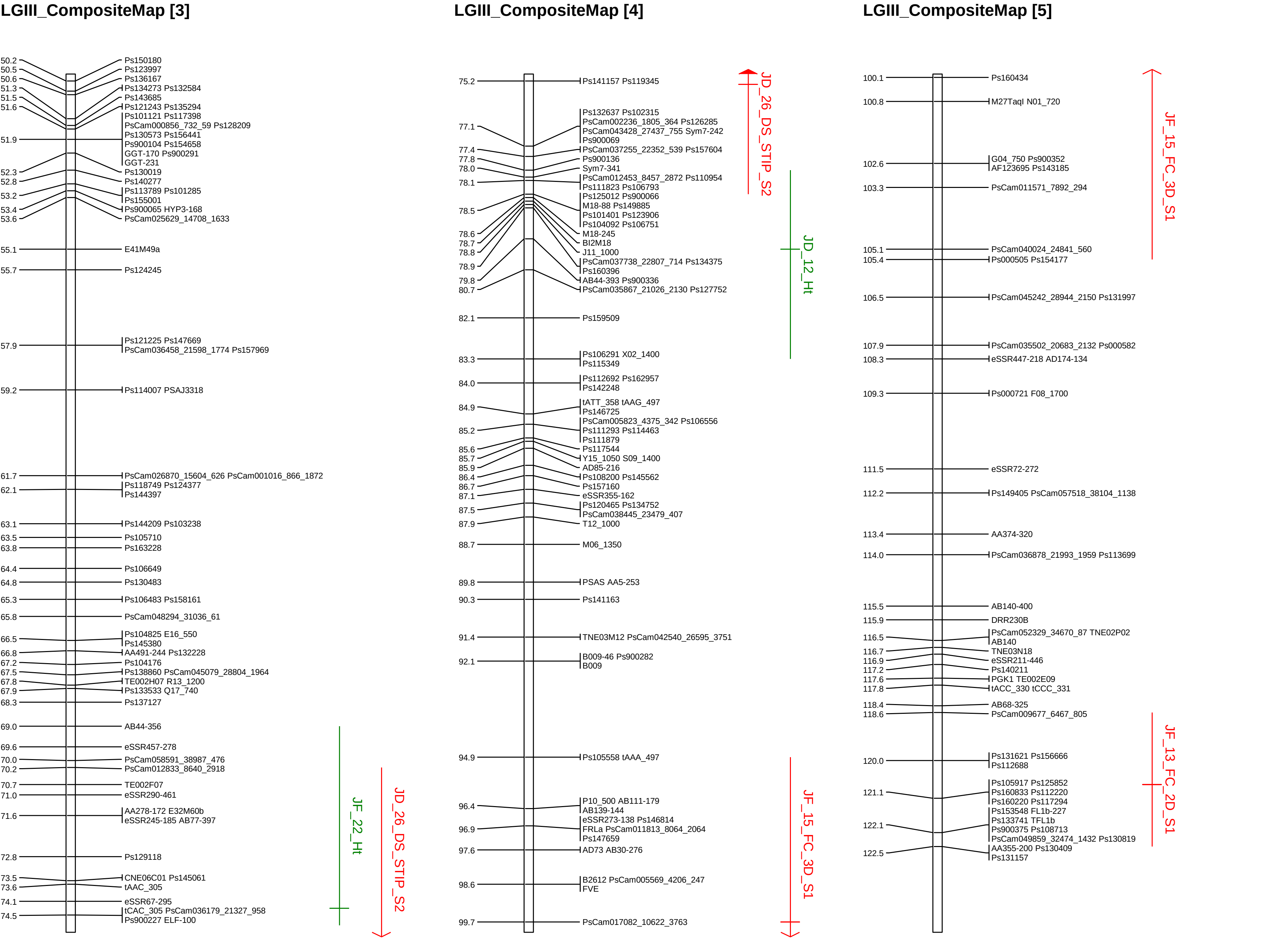

LGIII_CompositeMap [3]
50.2
50.5
50.6
51.3
51.5
51.6
51.9
52.3
52.8
53.2
53.4
53.6
55.1
55.7
57.9
59.2
61.7
62.1
63.1
63.5
63.8
64.4
64.8
65.3
65.8
66.5
66.8
67.2
67.5
67.8
67.9
68.3
69.0
69.6
70.0
70.2
70.7
71.0
71.6
72.8
73.5
73.6
74.1
74.5
Ps150180
Ps123997
Ps136167
Ps134273 Ps132584
Ps143685
Ps121243 Ps135294
Ps101121 Ps117398
PsCam000856_732_59 Ps128209
Ps130573 Ps156441
Ps900104 Ps154658
GGT-170 Ps900291
GGT-231
Ps130019
Ps140277
Ps113789 Ps101285
Ps155001
Ps900065 HYP3-168
PsCam025629_14708_1633
E41M49a
Ps124245
Ps121225 Ps147669
PsCam036458_21598_1774 Ps157969
Ps114007 PSAJ3318
PsCam026870_15604_626 PsCam001016_866_1872
Ps118749 Ps124377
Ps144397
Ps144209 Ps103238
Ps105710
Ps163228
Ps106649
Ps130483
Ps106483 Ps158161
PsCam048294_31036_61
Ps104825 E16_550
Ps145380
AA491-244 Ps132228
Ps104176
Ps138860 PsCam045079_28804_1964
TE002H07 R13_1200
Ps133533 Q17_740
Ps137127
AB44-356
eSSR457-278
PsCam058591_38987_476
PsCam012833_8640_2918
TE002F07
eSSR290-461
AA278-172 E32M60b
eSSR245-185 AB77-397
Ps129118
CNE06C01 Ps145061
tAAC_305
eSSR67-295
tCAC_305 PsCam036179_21327_958
Ps900227 ELF-100
JF_22_Ht
JD_26_DS_STIP_S2
LGIII_CompositeMap [4]
75.2
77.1
77.4
77.8
78.0
78.1
78.5
78.6
78.7
78.8
78.9
79.8
80.7
82.1
83.3
84.0
84.9
85.2
85.6
85.7
85.9
86.4
86.7
87.1
87.5
87.9
88.7
89.8
90.3
91.4
92.1
94.9
96.4
96.9
97.6
98.6
99.7
Ps141157 Ps119345
Ps132637 Ps102315
PsCam002236_1805_364 Ps126285
PsCam043428_27437_755 Sym7-242
Ps900069
PsCam037255_22352_539 Ps157604
Ps900136
Sym7-341
PsCam012453_8457_2872 Ps110954
Ps111823 Ps106793
Ps125012 Ps900066
M18-88 Ps149885
Ps101401 Ps123906
Ps104092 Ps106751
M18-245
BI2M18
J11_1000
PsCam037738_22807_714 Ps134375
Ps160396
AB44-393 Ps900336
PsCam035867_21026_2130 Ps127752
Ps159509
Ps106291 X02_1400
Ps115349
Ps112692 Ps162957
Ps142248
tATT_358 tAAG_497
Ps146725
PsCam005823_4375_342 Ps106556
Ps111293 Ps114463
Ps111879
Ps117544
Y15_1050 S09_1400
AD85-216
Ps108200 Ps145562
Ps157160
eSSR355-162
Ps120465 Ps134752
PsCam038445_23479_407
T12_1000
M06_1350
PSAS AA5-253
Ps141163
TNE03M12 PsCam042540_26595_3751
B009-46 Ps900282
B009
Ps105558 tAAA_497
P10_500 AB111-179
AB139-144
eSSR273-138 Ps146814
FRLa PsCam011813_8064_2064
Ps147659
AD73 AB30-276
B2612 PsCam005569_4206_247
FVE
PsCam017082_10622_3763
JD_26_DS_STIP_S2
JD_12_Ht
JF_15_FC_3D_S1
LGIII_CompositeMap [5]
100.1
100.8
102.6
103.3
105.1
105.4
106.5
107.9
108.3
109.3
111.5
112.2
113.4
114.0
115.5
115.9
116.5
116.7
116.9
117.2
117.6
117.8
118.4
118.6
120.0
121.1
122.1
122.5
Ps160434
M27TaqI N01_720
G04_750 Ps900352
AF123695 Ps143185
PsCam011571_7892_294
PsCam040024_24841_560
Ps000505 Ps154177
PsCam045242_28944_2150 Ps131997
PsCam035502_20683_2132 Ps000582
eSSR447-218 AD174-134
Ps000721 F08_1700
eSSR72-272
Ps149405 PsCam057518_38104_1138
AA374-320
PsCam036878_21993_1959 Ps113699
AB140-400
DRR230B
PsCam052329_34670_87 TNE02P02
AB140
TNE03N18
eSSR211-446
Ps140211
PGK1 TE002E09
tACC_330 tCCC_331
AB68-325
PsCam009677_6467_805
Ps131621 Ps156666
Ps112688
Ps105917 Ps125852
Ps160833 Ps112220
Ps160220 Ps117294
Ps153548 FL1b-227
Ps133741 TFL1b
Ps900375 Ps108713
PsCam049859_32474_1432 Ps130819
AA355-200 Ps130409
Ps131157
JF_15_FC_3D_S1
JF_13_FC_2D_S1

LGIII_CompositeMap [6]

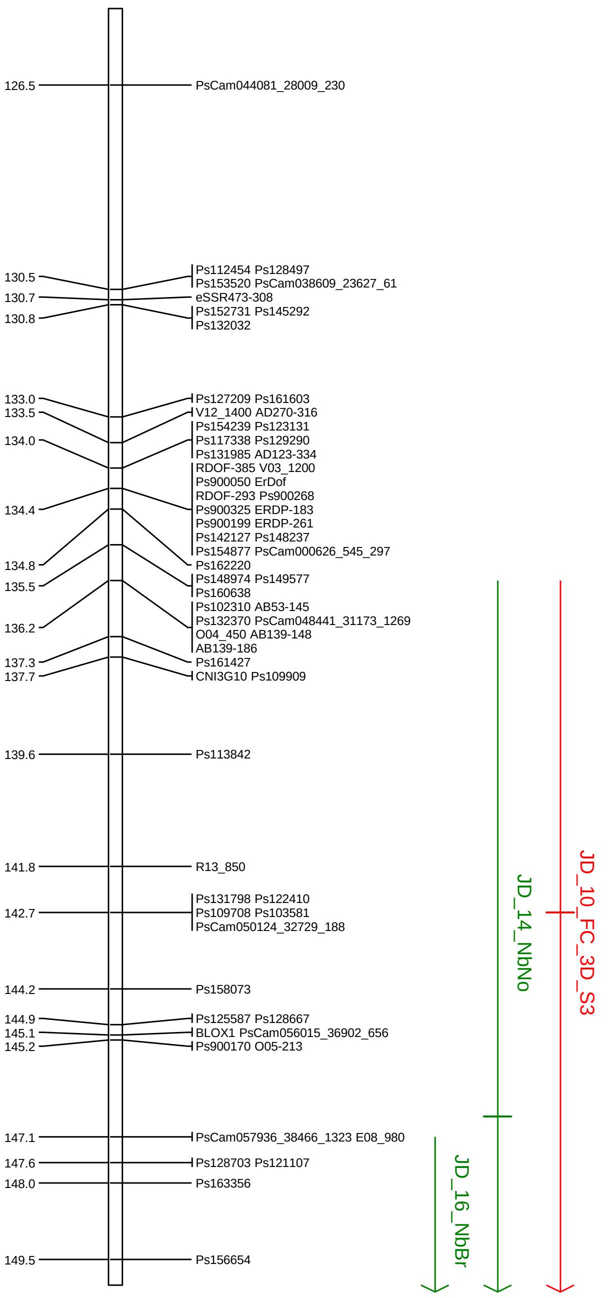

LGIII_CompositeMap [7]

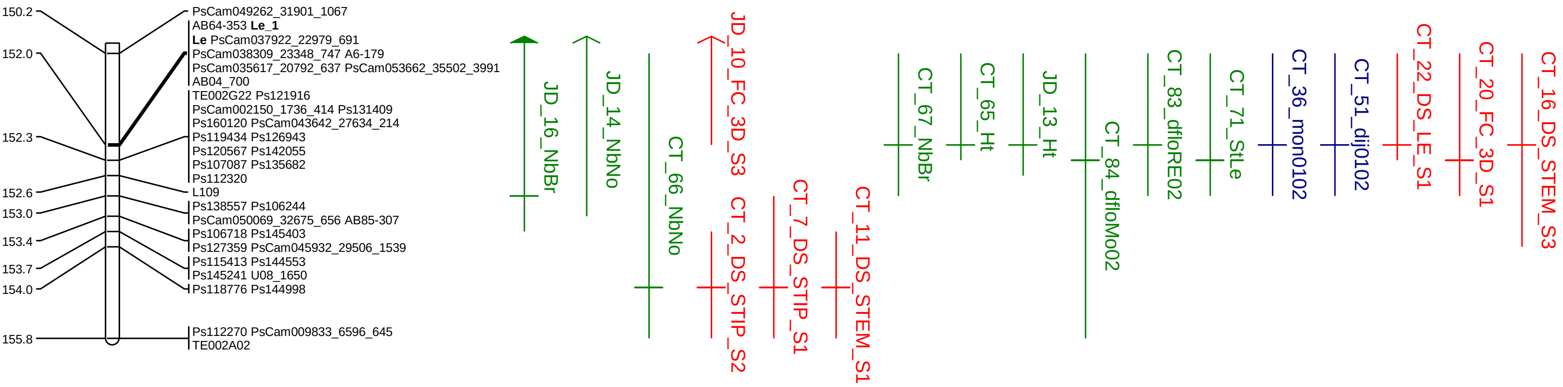

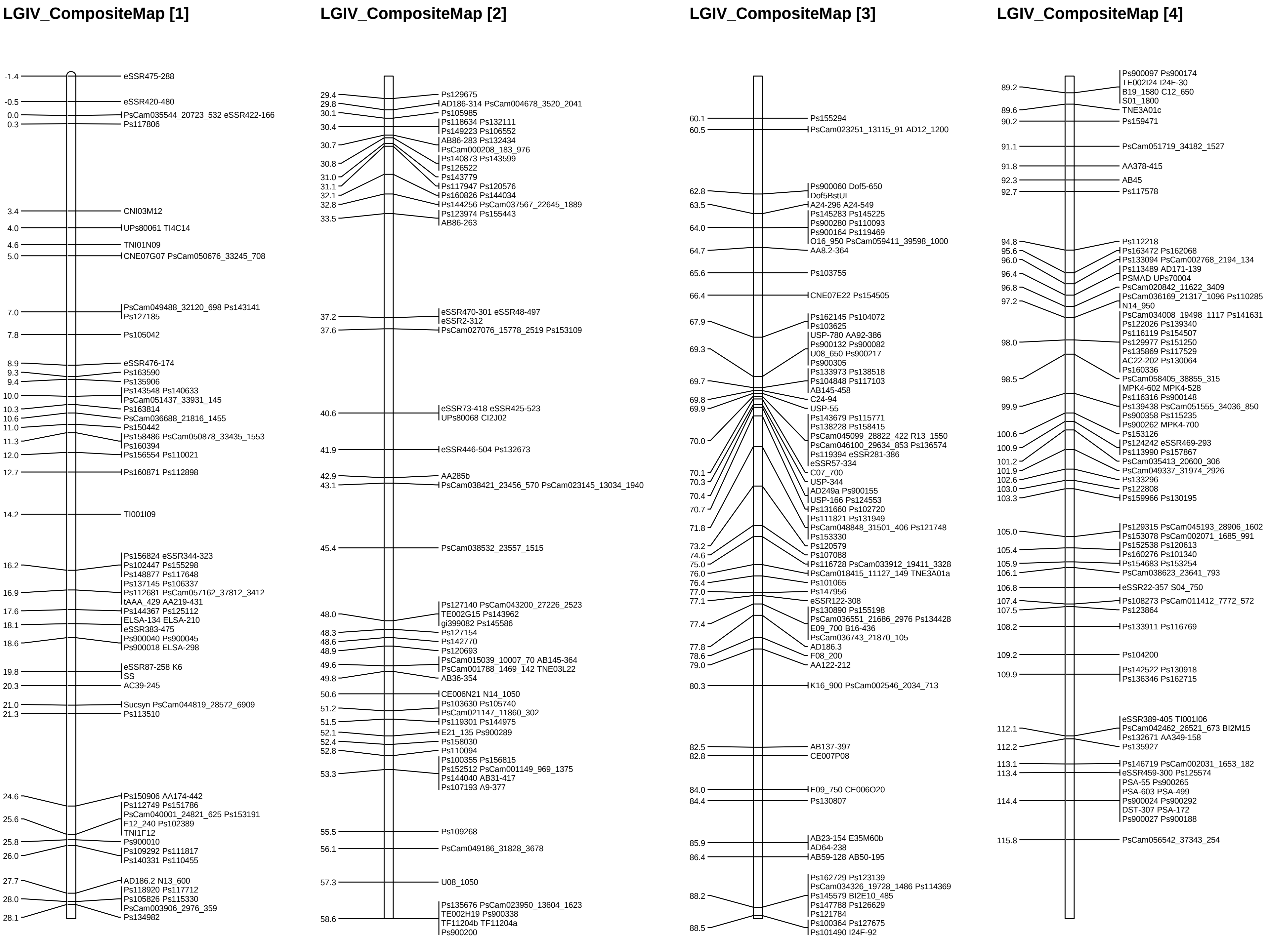
LGIV_CompositeMap [1]
-1.4 eSSR475-288
-0.5 eSSR420-480
0.0 PsCam035544_20723_532 eSSR422-166
0.3 Ps117806
3.4 CNI03M12
4.0 UPs80061 TI4C14
4.6 TNI01N09
5.0 CNE07G07 PsCam050676_33245_708
7.0 PsCam049488_32120_698 Ps143141
Ps127185
7.8 Ps105042
8.9 eSSR476-174
9.3 Ps163590
9.4 Ps135906
10.0 Ps143548 Ps140633
PsCam051437_33931_145
10.3 Ps163814
10.6 PsCam036688_21816_1455
11.0 Ps150442
11.3 Ps158486 PsCam050878_33435_1553
Ps160394
12.0 Ps156554 Ps110021
12.7 Ps160871 Ps112898
14.2 TI001I09
Ps156824 eSSR344-323
16.2 Ps102447 Ps155298
Ps148877 Ps117648
Ps137145 Ps106337
16.9 Ps112681 PsCam057162_37812_3412
tAAA_429 AA219-431
17.6 Ps144367 Ps125112
18.1 ELSA-134 ELSA-210
eSSR383-475
18.6 Ps900040 Ps900045
Ps900018 ELSA-298
19.8 eSSR87-258 K6
SS
20.3 AC39-245
21.0 Sucsyn PsCam044819_28572_6909
21.3 Ps113510
24.6 Ps150906 AA174-442
Ps112749 Ps151786
25.6 PsCam040001_24821_625 Ps153191
F12_240 Ps102389
TNI1F12
25.8 Ps900010
26.0 Ps109292 Ps111817
Ps140331 Ps110455
27.7 AD186.2 N13_600
Ps118920 Ps117712
28.0 Ps105826 Ps115330
PsCam003906_2976_359
28.1 Ps134982
LGIV_CompositeMap [2]
29.4 Ps129675
29.8 AD186-314 PsCam004678_3520_2041
30.1 Ps105985
30.4 Ps118634 Ps132111
Ps149223 Ps106552
30.7 AB86-283 Ps132434
PsCam000208_183_976
30.8 Ps140873 Ps143599
Ps126522
31.0 Ps143779
31.1 Ps117947 Ps120576
32.1 Ps160826 Ps144034
32.8 Ps144256 PsCam037567_22645_1889
33.5 Ps123974 Ps155443
AB86-263
37.2 eSSR470-301 eSSR48-497
eSSR2-312
37.6 PsCam027076_15778_2519 Ps153109
40.6 eSSR73-418 eSSR425-523
UPs80068 CI2J02
41.9 eSSR446-504 Ps132673
42.9 AA285b
43.1 PsCam038421_23456_570 PsCam023145_13034_1940
45.4 PsCam038532_23557_1515
Ps127140 PsCam043200_27226_2523
48.0 TE002G15 Ps143962
gi399082 Ps145586
48.3 Ps127154
48.6 Ps142770
48.9 Ps120693
49.6 PsCam015039_10007_70 AB145-364
PsCam001788_1469_142 TNE03L22
49.8 AB36-354
50.6 CE006N21 N14_1050
51.2 Ps103630 Ps105740
PsCam021147_11860_302
51.5 Ps119301 Ps144975
52.1 E21_135 Ps900289
52.4 Ps158030
52.8 Ps110094
Ps100355 Ps156815
53.3 Ps152512 PsCam001149_969_1375
Ps144040 AB31-417
Ps107193 A9-377
55.5 Ps109268
56.1 PsCam049186_31828_3678
57.3 U08_1050
Ps135676 PsCam023950_13604_1623
58.6 TE002H19 Ps900338
TF11204b TF11204a
Ps900200
LGIV_CompositeMap [3]
60.1 Ps155294
60.5 PsCam023251_13115_91 AD12_1200
62.8 Ps900060 Dof5-650
Dof5BstUI
63.5 A24-296 A24-549
Ps145283 Ps145225
64.0 Ps900280 Ps110093
Ps900164 Ps119469
O16_950 PsCam059411_39598_1000
64.7 AA8.2-364
65.6 Ps103755
66.4 CNE07E22 Ps154505
67.9 Ps162145 Ps104072
Ps103625
USP-780 AA92-386
69.3 Ps900132 Ps900082
U08_650 Ps900217
Ps900305
69.7 Ps133973 Ps138518
Ps104848 Ps117103
AB145-458
69.8 C24-94
69.9 USP-55
Ps143679 Ps115771
Ps138228 Ps158415
70.0 PsCam045099_28822_422 R13_1550
PsCam046100_29634_853 Ps136574
Ps119394 eSSR281-386
eSSR57-334
70.1 C07_700
70.3 USP-344
70.4 AD249a Ps900155
USP-166 Ps124553
70.7 Ps131660 Ps102720
71.8 Ps111821 Ps131949
PsCam048848_31501_406 Ps121748
Ps153330
73.2 Ps120579
74.6 Ps107088
75.0 Ps116728 PsCam033912_19411_3328
76.0 PsCam018415_11127_149 TNE3A01a
76.4 Ps101065
77.0 Ps147956
77.1 eSSR122-308
Ps130890 Ps155198
77.4 PsCam036551_21686_2976 Ps134428
E09_700 B16-436
PsCam036743_21870_105
77.8 AD186.3
78.6 F08_200
79.0 AA122-212
80.3 K16_900 PsCam002546_2034_713
82.5 AB137-397
82.8 CE007P08
84.0 E09_750 CE006O20
84.4 Ps130807
85.9 AB23-154 E35M60b
AD64-238
86.4 AB59-128 AB50-195
Ps162729 Ps123139
88.2 PsCam034326_19728_1486 Ps114369
Ps145579 BI2E10_485
Ps147788 Ps126629
Ps121784
88.5 Ps100364 Ps127675
Ps101490 I24F-92
LGIV_CompositeMap [4]
Ps900097 Ps900174
89.2 TE002I24 I24F-30
B19_1580 C12_650
S01_1800
89.6 TNE3A01c
90.2 Ps159471
91.1 PsCam051719_34182_1527
91.8 AA378-415
92.3 AB45
92.7 Ps117578
94.8 Ps112218
95.6 Ps163472 Ps162068
96.0 Ps133094 PsCam002768_2194_134
96.4 Ps113489 AD171-139
PSMAD UPs70004
96.8 PsCam020842_11622_3409
97.2 PsCam036169_21317_1096 Ps110285
N14_950
PsCam034008_19498_1117 Ps141631
Ps122026 Ps139340
Ps116119 Ps154507
98.0 Ps129977 Ps151250
Ps135869 Ps117529
AC22-202 Ps130064
Ps160336
98.5 PsCam058405_38855_315
MPK4-602 MPK4-528
Ps116316 Ps900148
99.9 Ps139438 PsCam051555_34036_850
Ps900358 Ps115235
Ps900262 MPK4-700
100.6 Ps153126
100.9 Ps124242 eSSR469-293
Ps113990 Ps157867
101.2 PsCam035413_20600_306
101.9 PsCam049337_31974_2926
102.6 Ps133296
103.0 Ps122808
103.3 Ps159966 Ps130195
105.0 Ps129315 PsCam045193_28906_1602
Ps153078 PsCam002071_1685_991
105.4 Ps152538 Ps120613
Ps160276 Ps101340
105.9 Ps154683 Ps153254
106.1 PsCam038623_23641_793
106.8 eSSR22-357 S04_750
107.4 Ps108273 PsCam011412_7772_572
107.5 Ps123864
108.2 Ps133911 Ps116769
109.2 Ps104200
109.9 Ps142522 Ps130918
Ps136346 Ps162715
eSSR389-405 TI001I06
112.1 PsCam042462_26521_673 BI2M15
Ps132671 AA349-158
112.2 Ps135927
113.1 Ps146719 PsCam002031_1653_182
113.4 eSSR459-300 Ps125574
PSA-55 Ps900265
PSA-603 PSA-499
114.4 Ps900024 Ps900292
DST-307 PSA-172
Ps900027 Ps900188
115.8 PsCam056542_37343_254

LGIV_CompositeMap [5]

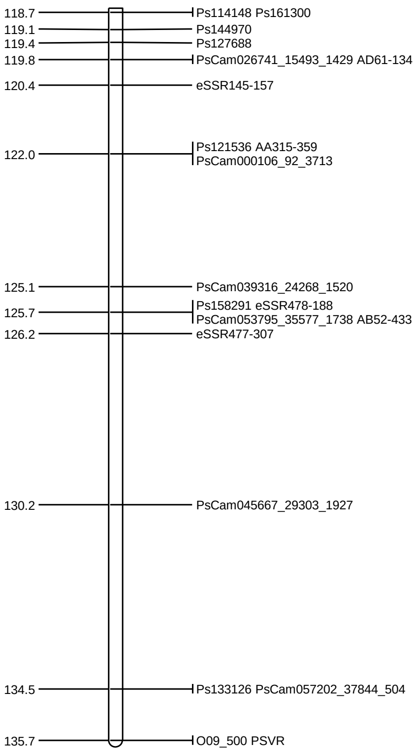

LGV_CompositeMap [1]

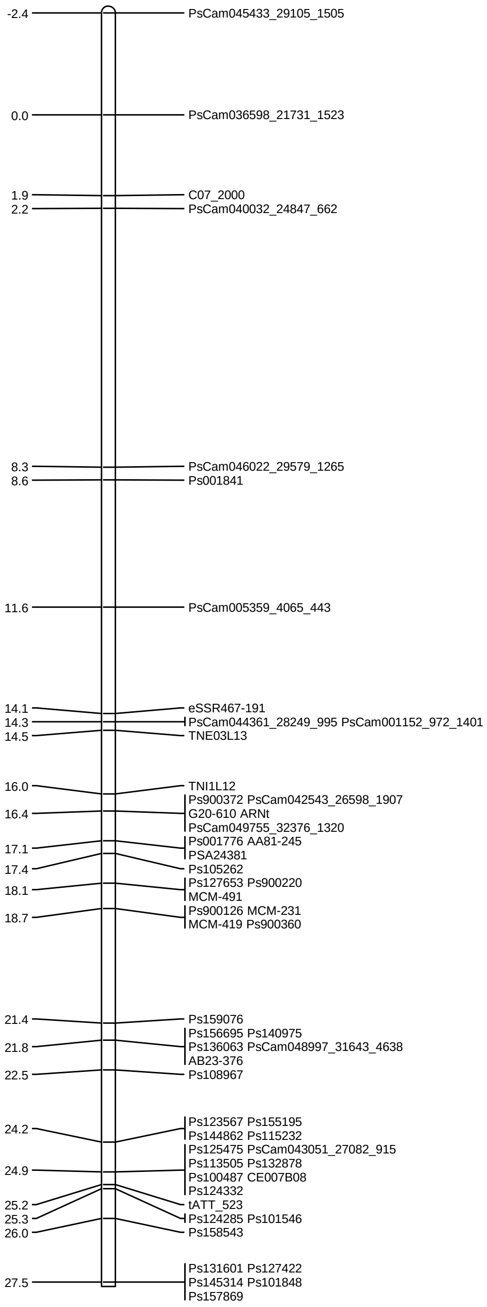

LGV_CompositeMap [2]

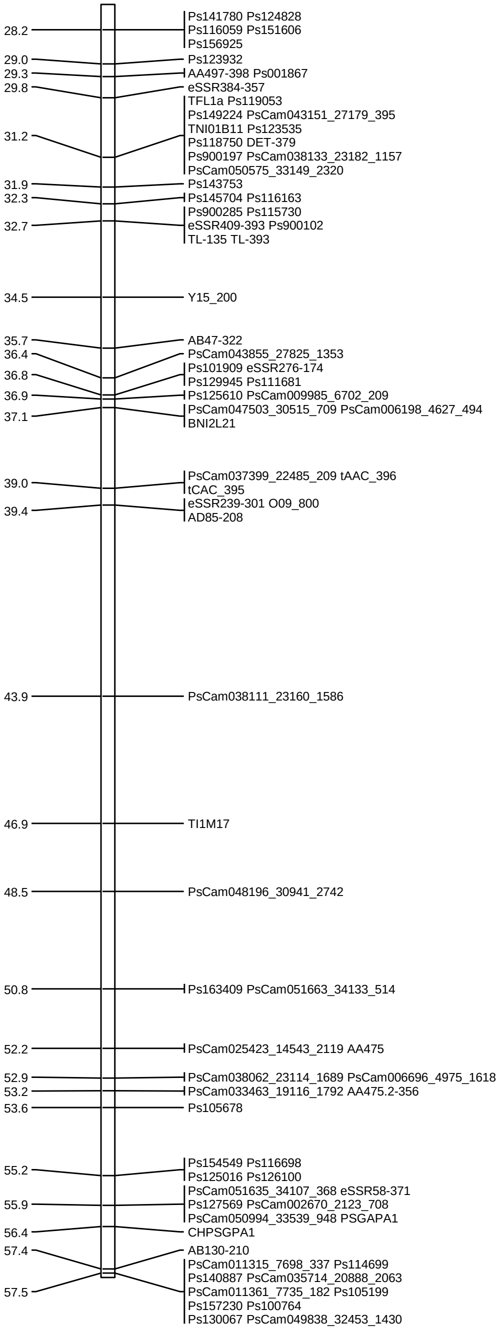

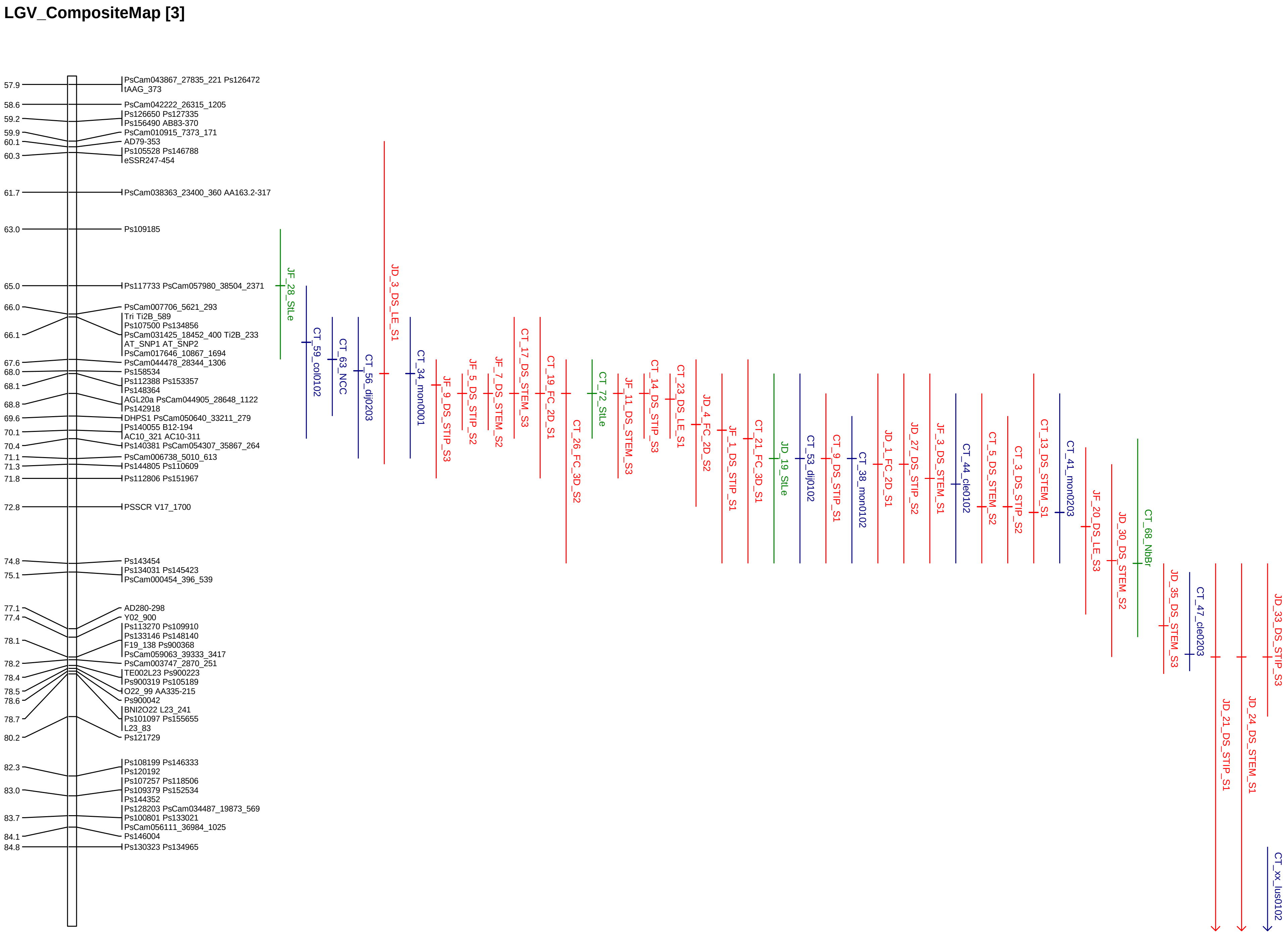
LGV_CompositeMap [3]
57.9 PsCam043867_27835_221 Ps126472
tAAG_373
58.6 PsCam042222_26315_1205
59.2 Ps126650 Ps127335
Ps156490 AB83-370
59.9 PsCam010915_7373_171
60.1 AD79-353
60.3 Ps105528 Ps146788
eSSR247-454
61.7 PsCam038363_23400_360 AA163.2-317
63.0 Ps109185
65.0 Ps117733 PsCam057980_38504_2371
66.0 PsCam007706_5621_293
Tri Ti2B_589
Ps107500 Ps134856
66.1 PsCam031425_18452_400 Ti2B_233
AT_SNP1 AT_SNP2
PsCam017646_10867_1694
67.6 PsCam044478_28344_1306
68.0 Ps158534
68.1 Ps112388 Ps153357
Ps148364
68.8 AGL20a PsCam044905_28648_1122
Ps142918
69.6 DHPS1 PsCam050640_33211_279
70.1 Ps140055 B12-194
AC10_321 AC10-311
70.4 Ps140381 PsCam054307_35867_264
71.1 PsCam006738_5010_613
71.3 Ps144805 Ps110609
71.8 Ps112806 Ps151967
72.8 PSSCR V17_1700
74.8 Ps143454
75.1 Ps134031 Ps145423
PsCam000454_396_539
77.1 AD280-298
77.4 Y02_900
Ps113270 Ps109910
78.1 Ps133146 Ps148140
F19_138 Ps900368
PsCam059063_39333_3417
78.2 PsCam003747_2870_251
78.4 TE002L23 Ps900223
Ps900319 Ps105189
78.5 O22_99 AA335-215
78.6 Ps900042
BNI2O22 L23_241
78.7 Ps101097 Ps155655
L23_83
80.2 Ps121729
82.3 Ps108199 Ps146333
Ps120192
83.0 Ps107257 Ps118506
Ps109379 Ps152534
Ps144352
83.7 Ps128203 PsCam034487_19873_569
Ps100801 Ps133021
PsCam056111_36984_1025
84.1 Ps146004
84.8 Ps130323 Ps134965
JF_28_StLe
CT_59_col0102
CT_63_NCC
CT_56_dij0203
JD_3_DS_LE_S1
CT_34_mon0001
JF_9_DS_STIP_S3
JF_5_DS_STIP_S2
JF_7_DS_STEM_S2
CT_17_DS_STEM_S3
CT_19_FC_2D_S1
CT_26_FC_3D_S2
CT_72_StLe
JF_11_DS_STEM_S3
CT_14_DS_STIP_S3
CT_23_DS_LE_S1
JD_4_FC_2D_S2
JF_1_DS_STIP_S1
CT_21_FC_3D_S1
JD_19_StLe
CT_53_dij0102
CT_9_DS_STIP_S1
CT_38_mon0102
JD_1_FC_2D_S1
JD_27_DS_STIP_S2
JF_3_DS_STEM_S1
CT_44_cle0102
CT_5_DS_STEM_S2
CT_3_DS_STIP_S2
CT_13_DS_STEM_S1
CT_41_mon0203
JF_20_DS_LE_S3
JD_30_DS_STEM_S2
CT_68_NbBr
JD_35_DS_STEM_S3
CT_47_cle0203
JD_21_DS_STIP_S1
JD_24_DS_STEM_S1
JD_33_DS_STIP_S3
CT_xx_lus0102

LGV_CompositeMap [3]

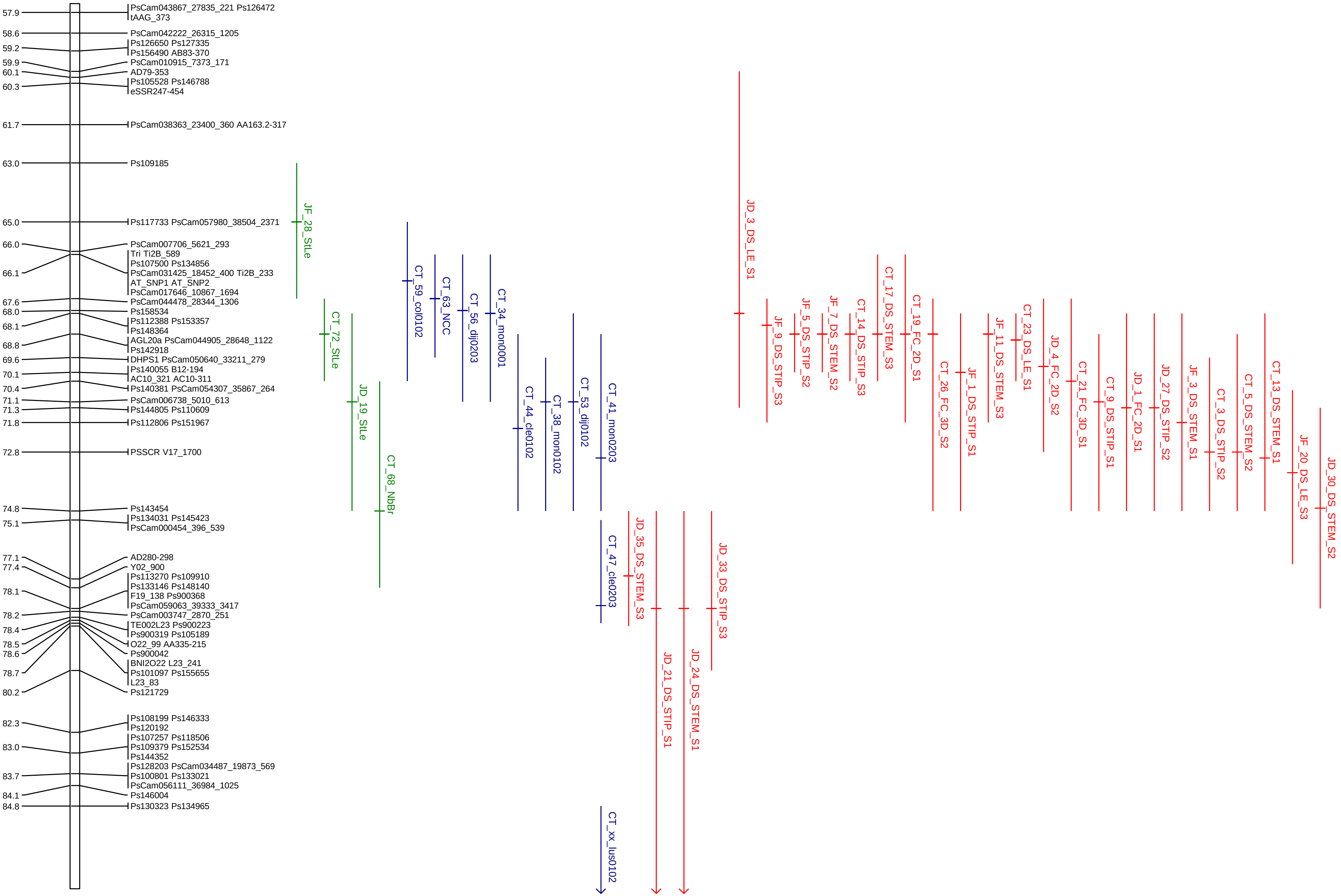

LGV_CompositeMap [4]

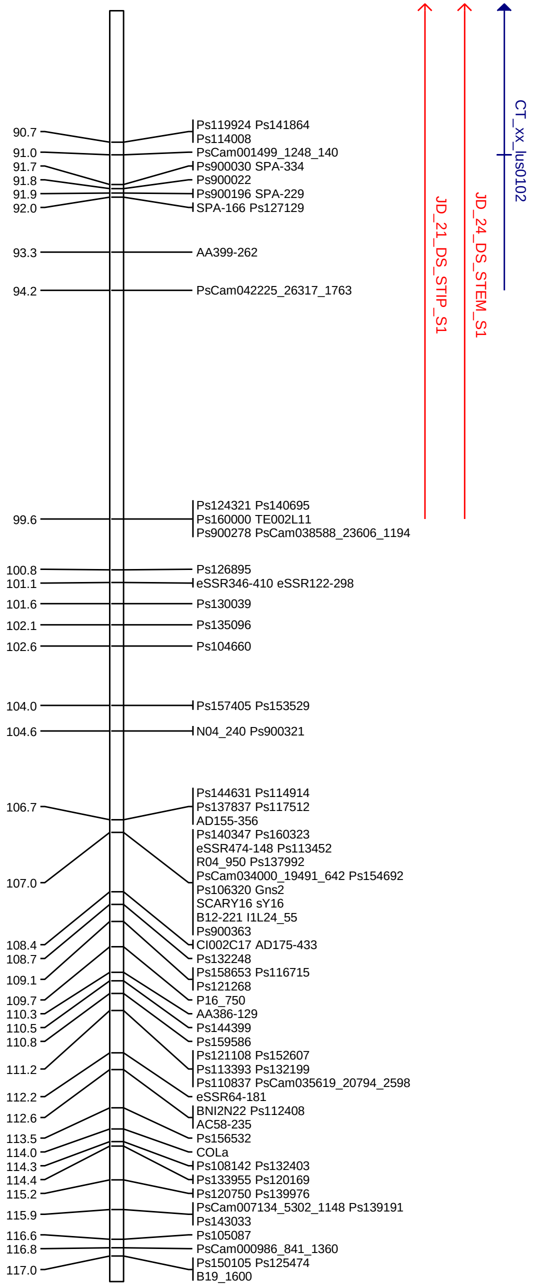

LGV_CompositeMap [5]

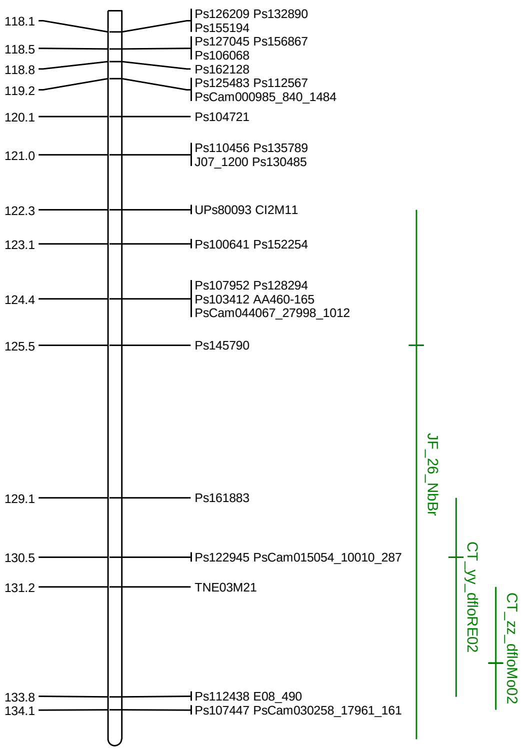

LGVI_CompositeMap [1]

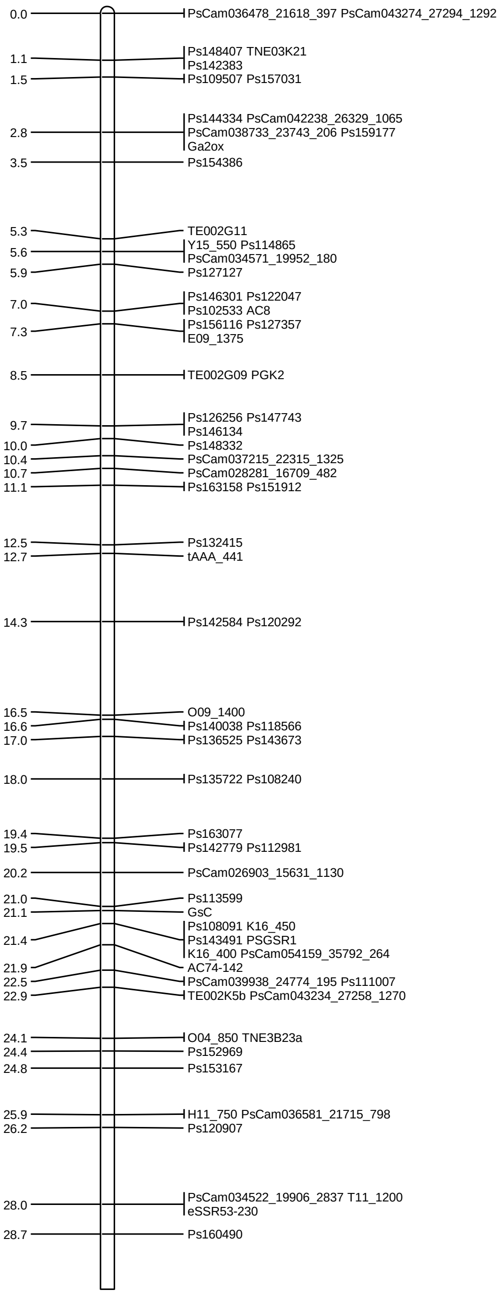

LGVI_CompositeMap [2]

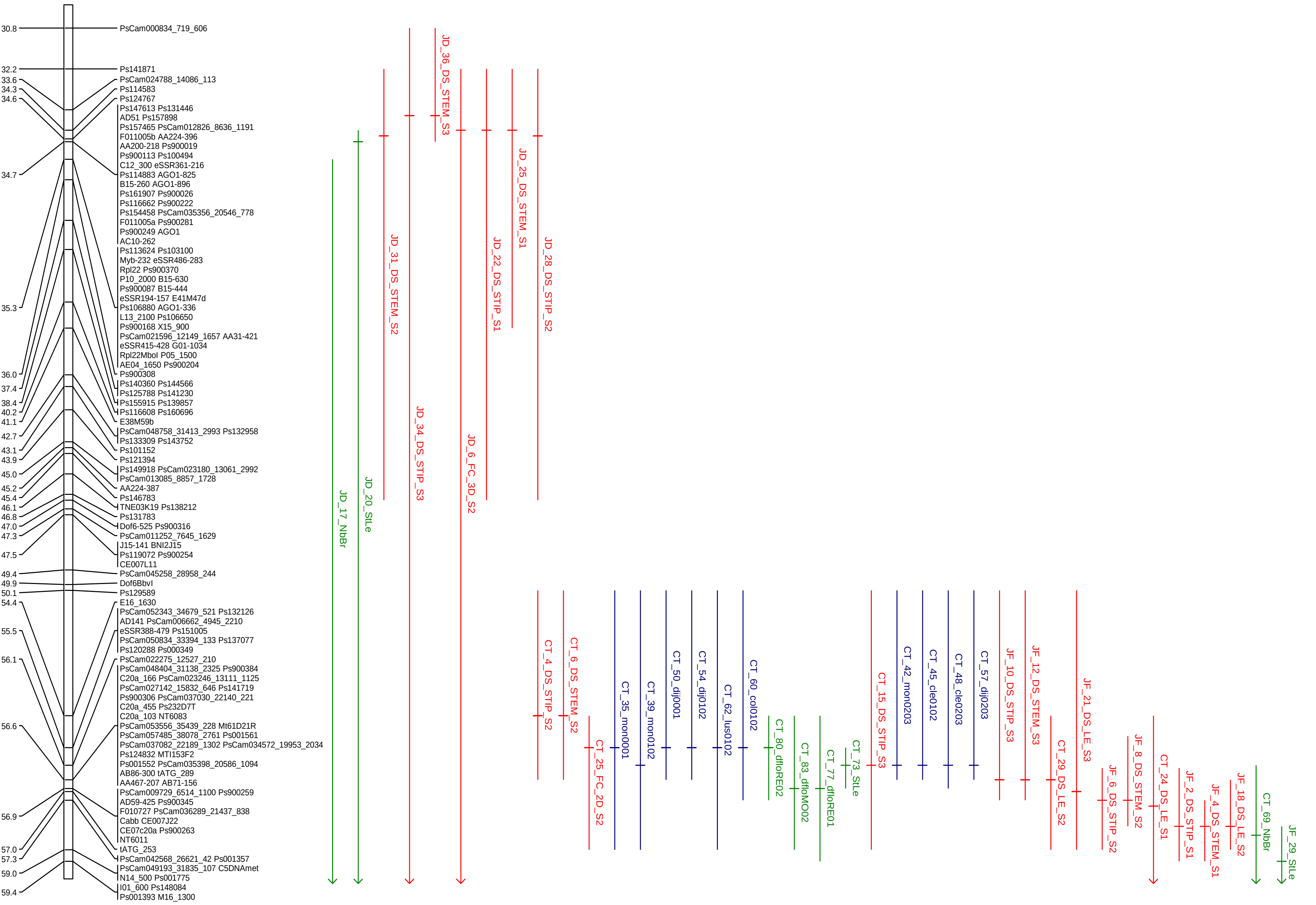

LGVI_CompositeMap [3]

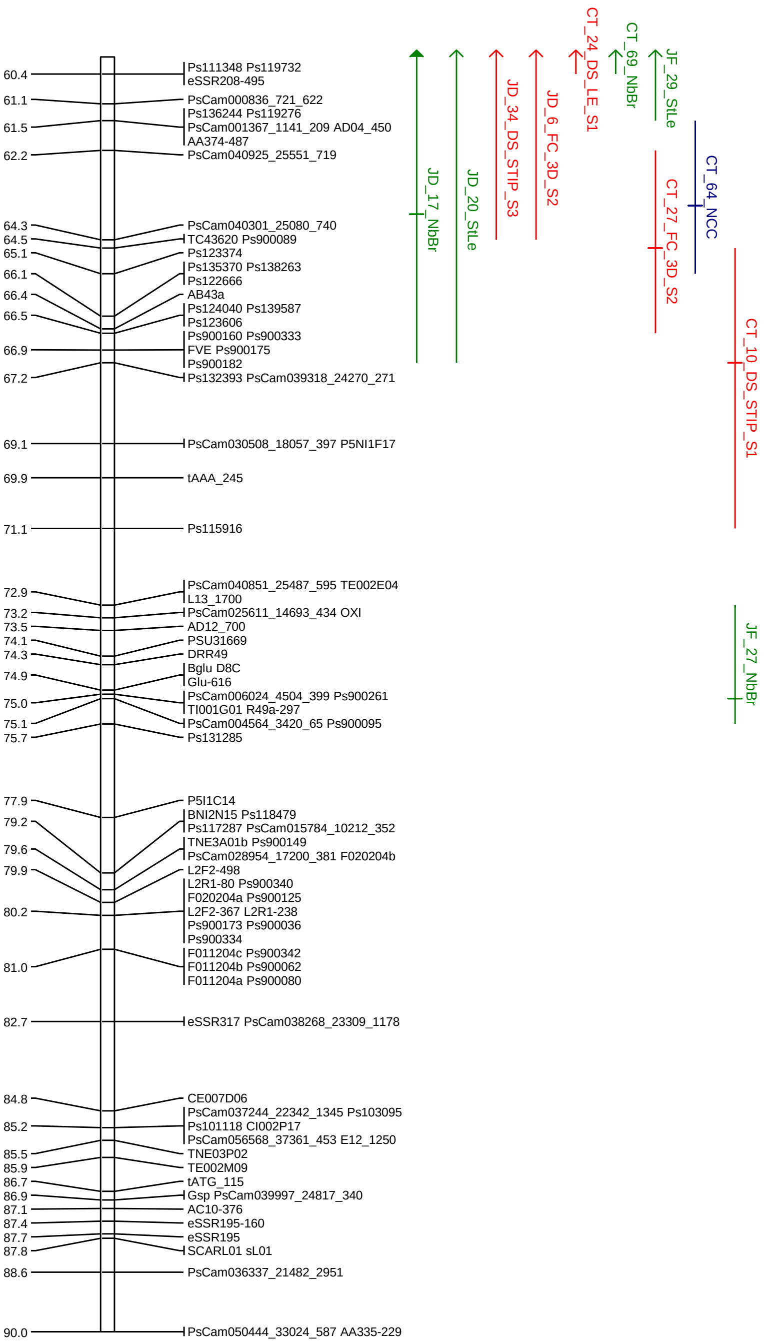

LGVI_CompositeMap [4]

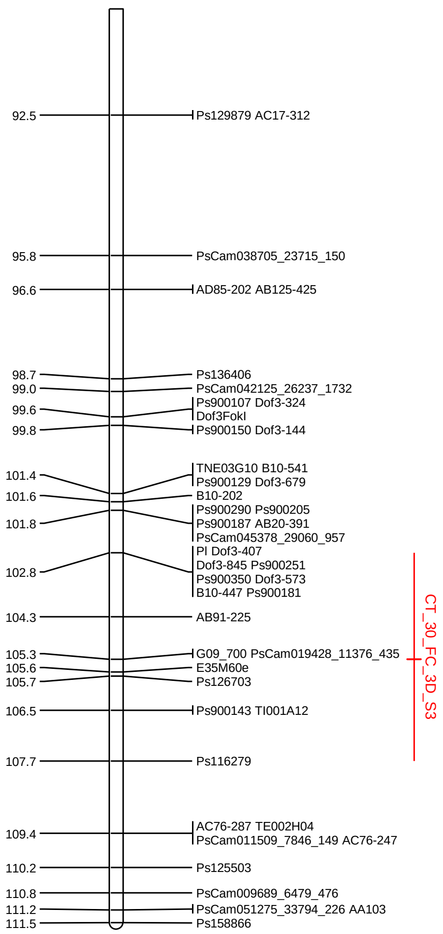

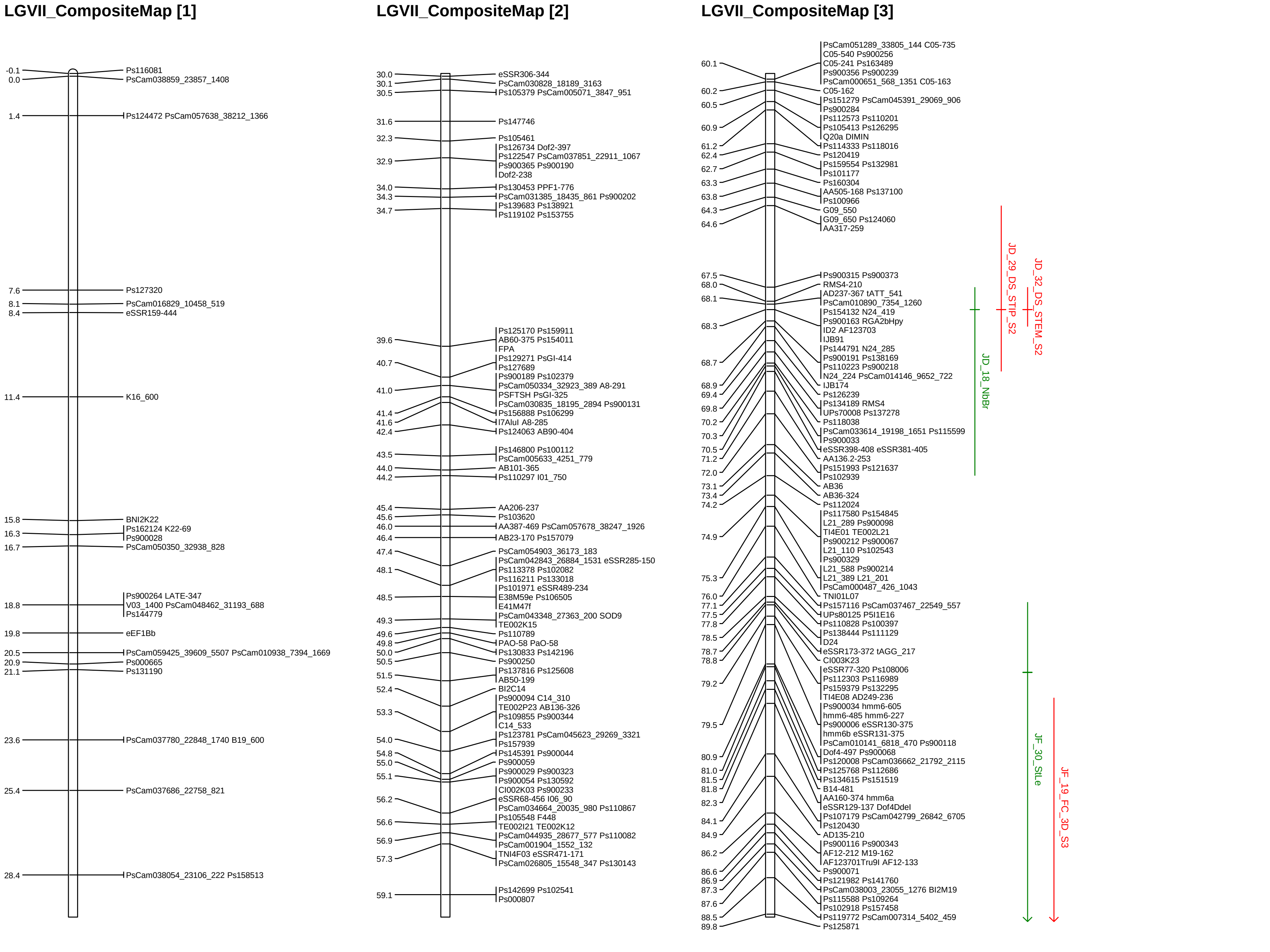
LGVII_CompositeMap [1]
-0.1
0.0
1.4
7.6
8.1
8.4
11.4
15.8
16.3
16.7
18.8
19.8
20.5
20.9
21.1
23.6
25.4
28.4
Ps116081
PsCam038859_23857_1408
Ps124472 PsCam057638_38212_1366
Ps127320
PsCam016829_10458_519
eSSR159-444
K16_600
BNI2K22
Ps162124 K22-69
Ps900028
PsCam050350_32938_828
Ps900264 LATE-347
V03_1400 PsCam048462_31193_688
Ps144779
eEF1Bb
PsCam059425_39609_5507 PsCam010938_7394_1669
Ps000665
Ps131190
PsCam037780_22848_1740 B19_600
PsCam037686_22758_821
PsCam038054_23106_222 Ps158513
LGVII_CompositeMap [2]
30.0
30.1
30.5
31.6
32.3
32.9
34.0
34.3
34.7
39.6
40.7
41.0
41.4
41.6
42.4
43.5
44.0
44.2
45.4
45.6
46.0
46.4
47.4
48.1
48.5
49.3
49.6
49.8
50.0
50.5
51.5
52.4
53.3
54.0
54.8
55.0
55.1
56.2
56.6
56.9
57.3
59.1
eSSR306-344
PsCam030828_18189_3163
Ps105379 PsCam005071_3847_951
Ps147746
Ps105461
Ps126734 Dof2-397
Ps122547 PsCam037851_22911_1067
Ps900365 Ps900190
Dof2-238
Ps130453 PPF1-776
PsCam031385_18435_861 Ps900202
Ps139683 Ps138921
Ps119102 Ps153755
Ps125170 Ps159911
AB60-375 Ps154011
FPA
Ps129271 PsGI-414
Ps127689
Ps900189 Ps102379
PsCam050334_32923_389 A8-291
PSFTSH PsGI-325
PsCam030835_18195_2894 Ps900131
Ps156888 Ps106299
I7AluI A8-285
Ps124063 AB90-404
Ps146800 Ps100112
PsCam005633_4251_779
AB101-365
Ps110297 I01_750
AA206-237
Ps103620
AA387-469 PsCam057678_38247_1926
AB23-170 Ps157079
PsCam054903_36173_183
PsCam042843_26884_1531 eSSR285-150
Ps113378 Ps102082
Ps116211 Ps133018
Ps101971 eSSR489-234
E38M59e Ps106505
E41M47f
PsCam043348_27363_200 SOD9
TE002K15
Ps110789
PAO-58 PaO-58
Ps130833 Ps142196
Ps900250
Ps137816 Ps125608
AB50-199
BI2C14
Ps900094 C14_310
TE002P23 AB136-326
Ps109855 Ps900344
C14_533
Ps123781 PsCam045623_29269_3321
Ps157939
Ps145391 Ps900044
Ps900059
Ps900029 Ps900323
Ps900054 Ps130592
CI002K03 Ps900233
eSSR68-456 I06_90
PsCam034664_20035_980 Ps110867
Ps105548 F448
TE002I21 TE002K12
PsCam044935_28677_577 Ps110082
PsCam001904_1552_132
TNI4F03 eSSR471-171
PsCam026805_15548_347 Ps130143
Ps142699 Ps102541
Ps000807
LGVII_CompositeMap [3]
60.1
60.2
60.5
60.9
61.2
62.4
62.7
63.3
63.8
64.3
64.6
67.5
68.0
68.1
68.3
68.7
68.9
69.4
69.8
70.2
70.3
70.5
71.2
72.0
73.1
73.4
74.2
74.9
75.3
76.0
77.1
77.5
77.8
78.5
78.7
78.8
79.2
79.5
80.9
81.0
81.5
81.8
82.3
84.1
84.9
86.2
86.6
86.9
87.3
87.6
88.5
89.8
PsCam051289_33805_144 C05-735
C05-540 Ps900256
C05-241 Ps163489
Ps900356 Ps900239
PsCam000651_568_1351 C05-163
C05-162
Ps151279 PsCam045391_29069_906
Ps900284
Ps112573 Ps110201
Ps105413 Ps126295
Q20a DIMIN
Ps114333 Ps118016
Ps120419
Ps159554 Ps132981
Ps101177
Ps160304
AA505-168 Ps137100
Ps100966
G09_550
G09_650 Ps124060
AA317-259
Ps900315 Ps900373
RMS4-210
AD237-367 tATT_541
PsCam010890_7354_1260
Ps154132 N24_419
Ps900163 RGA2bHpy
ID2 AF123703
IJB91
Ps144791 N24_285
Ps900191 Ps138169
Ps110223 Ps900218
N24_224 PsCam014146_9652_722
IJB174
Ps126239
Ps134189 RMS4
UPs70008 Ps137278
Ps118038
PsCam033614_19198_1651 Ps115599
Ps900033
eSSR398-408 eSSR381-405
AA136.2-253
Ps151993 Ps121637
Ps102939
AB36
AB36-324
Ps112024
Ps117580 Ps154845
L21_289 Ps900098
TI4E01 TE002L21
Ps900212 Ps900067
L21_110 Ps102543
Ps900329
L21_588 Ps900214
L21_389 L21_201
PsCam000487_426_1043
TNI01L07
Ps157116 PsCam037467_22549_557
UPs80125 P5I1E16
Ps110828 Ps100397
Ps138444 Ps111129
D24
eSSR173-372 tAGG_217
CI003K23
eSSR77-320 Ps108006
Ps112303 Ps116989
Ps159379 Ps132295
TI4E08 AD249-236
Ps900034 hmm6-605
hmm6-485 hmm6-227
Ps900006 eSSR130-375
hmm6b eSSR131-375
PsCam010141_6818_470 Ps900118
Dof4-497 Ps900068
Ps120008 PsCam036662_21792_2115
Ps125768 Ps112686
Ps134615 Ps151519
B14-481
AA160-374 hmm6a
eSSR129-137 Dof4DdeI
Ps107179 PsCam042799_26842_6705
Ps120430
AD135-210
Ps900116 Ps900343
AF12-212 M19-162
AF123701Tru9I AF12-133
Ps900071
Ps121982 Ps141760
PsCam038003_23055_1276 BI2M19
Ps115588 Ps109264
Ps102918 Ps157458
Ps119772 PsCam007314_5402_459
Ps125871
JD_29_DS_STIP_S2
JD_32_DS_STEM_S2
JD_18_NbBr
JF_30_StLe
JF_19_FC_3D_S3

LGVII_CompositeMap [4]

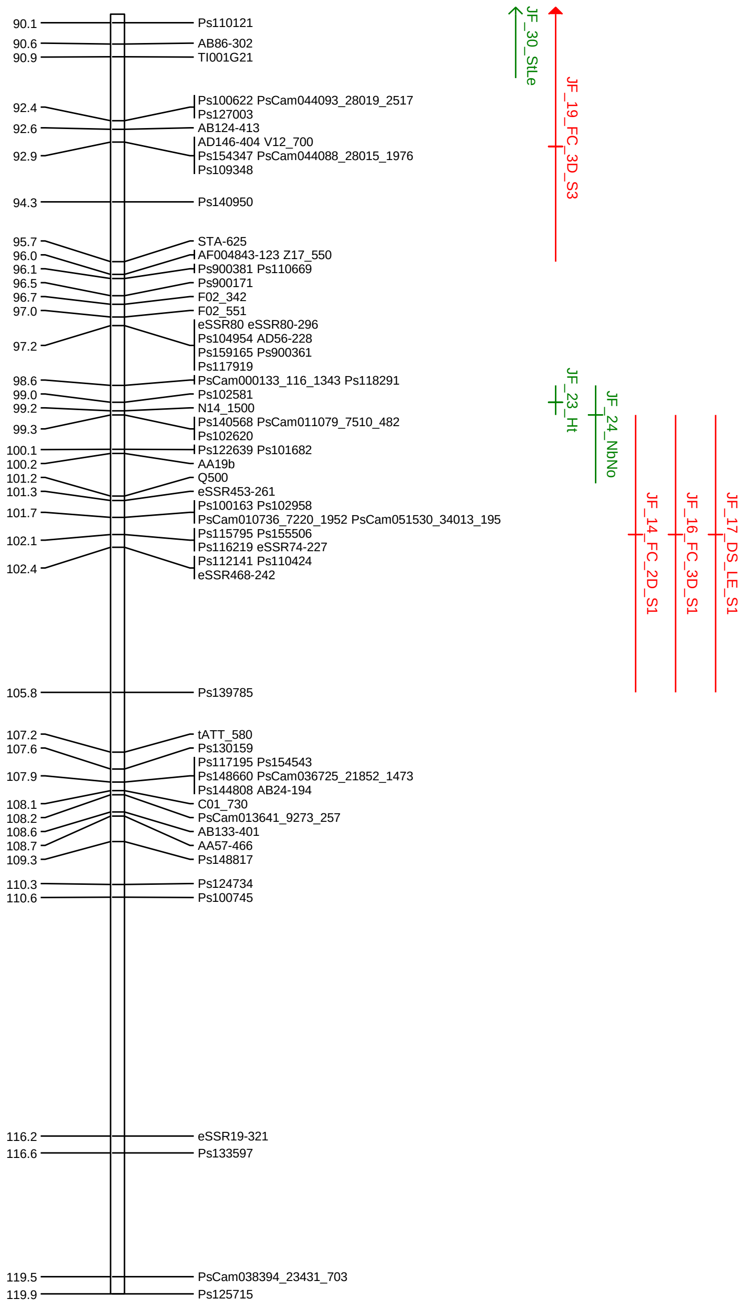

LGVII_CompositeMap [5]

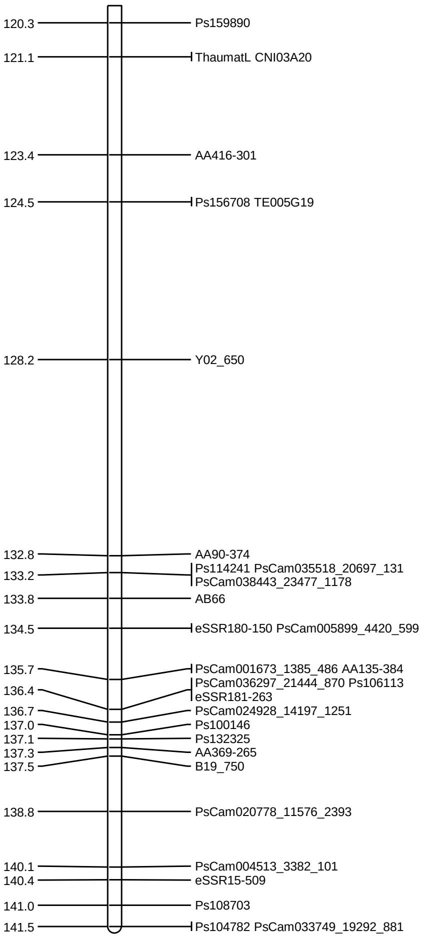

Supplement: Supplementary file 1 [file genes-14-01399-s001.zip › genes-2286623 - Figure S3.pdf]
