# Supplementary material for: Five Regions of the Pea Genome Co-Control Partial Resistance to D. pinodes, Tolerance to Frost, and Some Architectural or Phenological Traits
Source: Genes (Basel). 2023 Jul 4;14(7):1399. doi: 10.3390/genes14071399 (PMC10379203; doi:10.3390/genes14071399)
Supplement: Supplementary file 1 [file genes-14-01399-s001.zip › genes-2286623 - Table S1.pdf]

Additional File 3

Table S1 **Broad sense heritability for *D. pinodes* disease severity under controlled conditions and architectural traits variables for ChxTe, JIxDP, and JIxFP RIL populations**

Criteria	Champagne x Terese	JI296 x DP	JI296 x FP
**FC_2D_S1**	0.81	0.87	0.72
**FC_3D_S1**	0.56	-	0.68
**DS_LE_S1**	0.90	0.85	0.79
**FC_2D_S2**	0.67	0.78	0.29
**FC_3D_S2**	0.65	0.79	0.36
**DS_LE_S2**	0.71	0.79	0.56
**FC_2D_S3**	-	0.54	0.58
**FC_3D_S3**	0.67	0.60	0.58
**DS_LE_S3**	0.81	0.53	0.57
**DS_STIP_S1**	0.78	0.62	0.44
**DS_STEM_S1**	0.68	0.61	0.31
**DS_STIP_S2**	0.68	0.91	0.66
**DS_STEM_S2**	0.62	0.90	0.53
**DS_STIP_S3**	0.68	0.64	0.35
**DS_STEM_S3**	0.62	0.60	0.37
**Ht**	0.97	0.97	0.84
**NbNo**	0.49	0.22	0.28
**NbBr**	0.92	0.85	0.79
**StLe**	0.96	0.85	0.86

Disease severity nomenclature : DS : AUDPC of disease severity assessed on whole plantlet stipules (STIP) or stems (STEM) or on detached stipules (LE) ; FC : flecks coalescence assessed on detached stipules two (2D) or three (3D) days after inoculation ; Sx : *D. pinodes* strain of high (S1), intermediate (S2) or low (S3) aggressiveness on pea ;
Architectural traits nomenclature : StLe : stipule length ; NbBr : number of branches ; NbNo : number of nodes ; Ht : plant height (see Material and Methods section for details)
