# Supplementary figures and images for: Five Regions of the Pea Genome Co-Control Partial Resistance to D. pinodes, Tolerance to Frost, and Some Architectural or Phenological Traits
Source: Genes (Basel). 2023 Jul 4;14(7):1399. doi: 10.3390/genes14071399 (PMC10379203; doi:10.3390/genes14071399)

## LG1\_CT

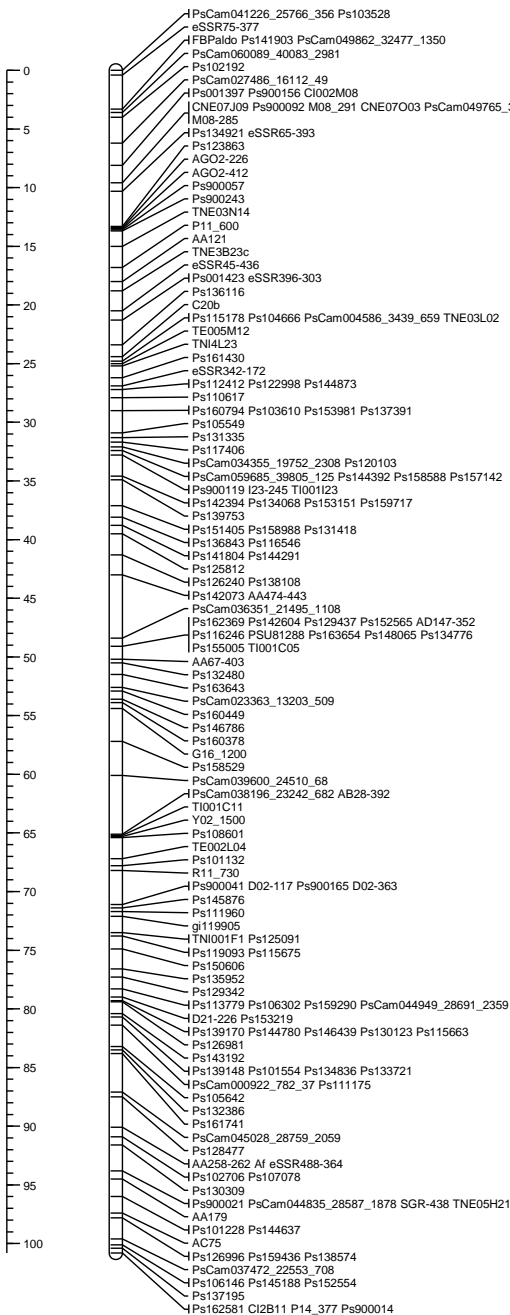

## LG1\_JF

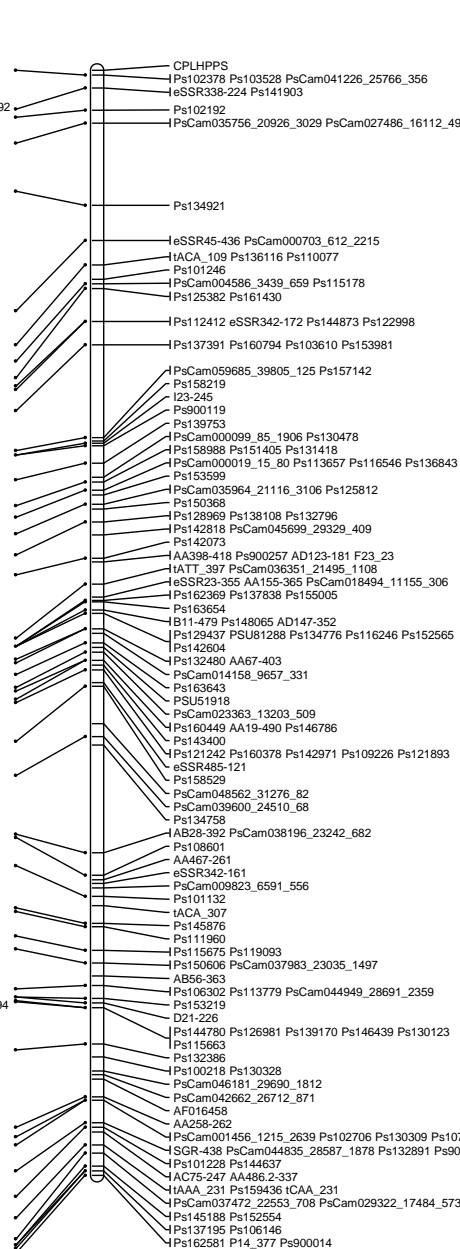

## LG1\_JD

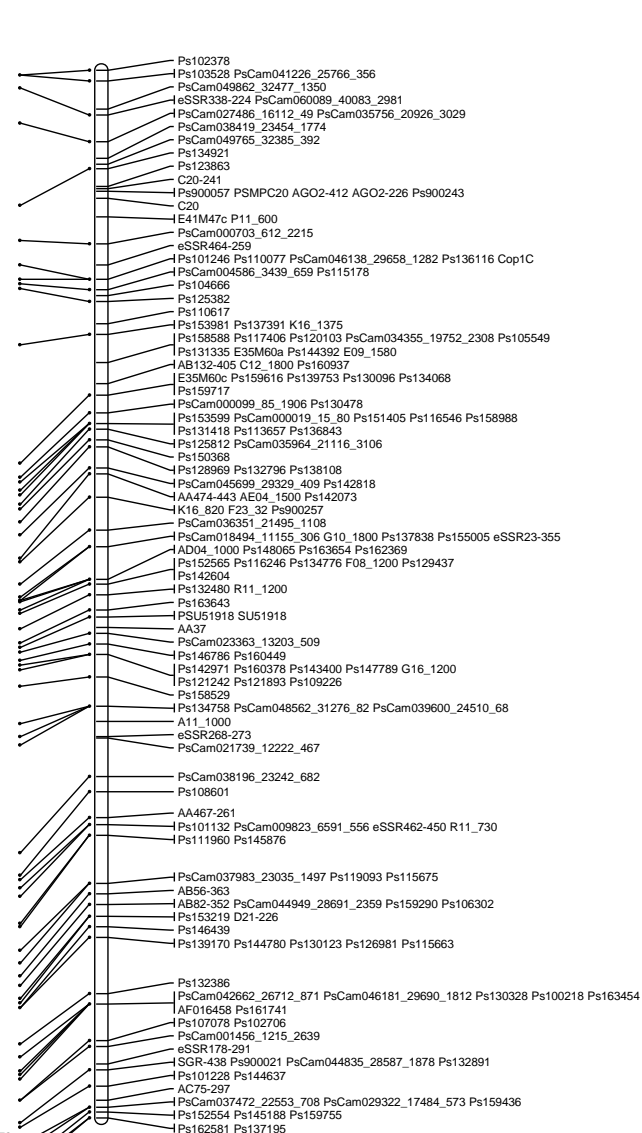

## LG2\_CT

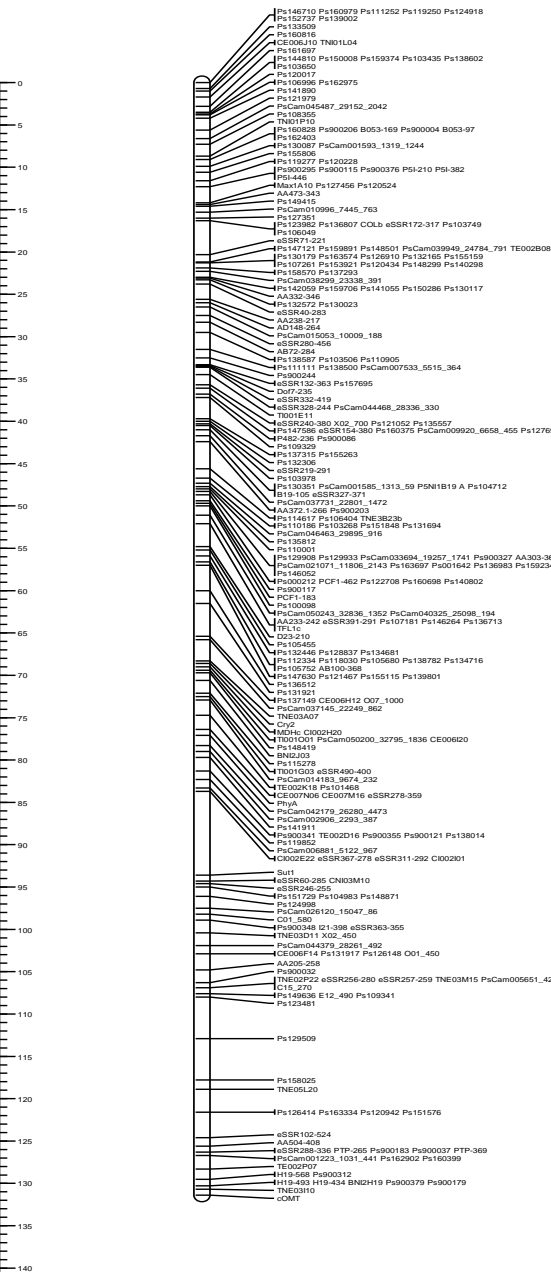

## LG2\_JF

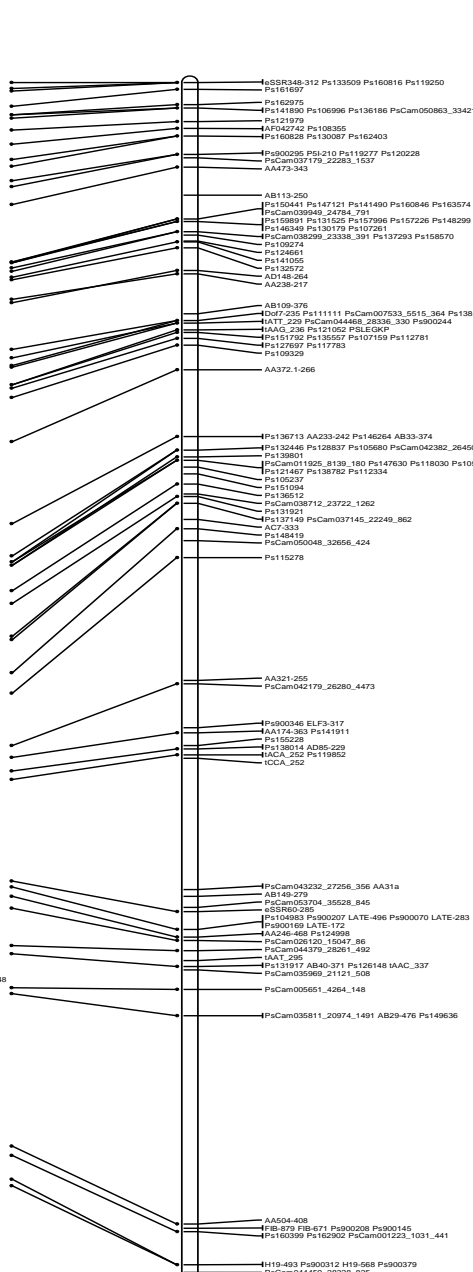

## LG2\_JD

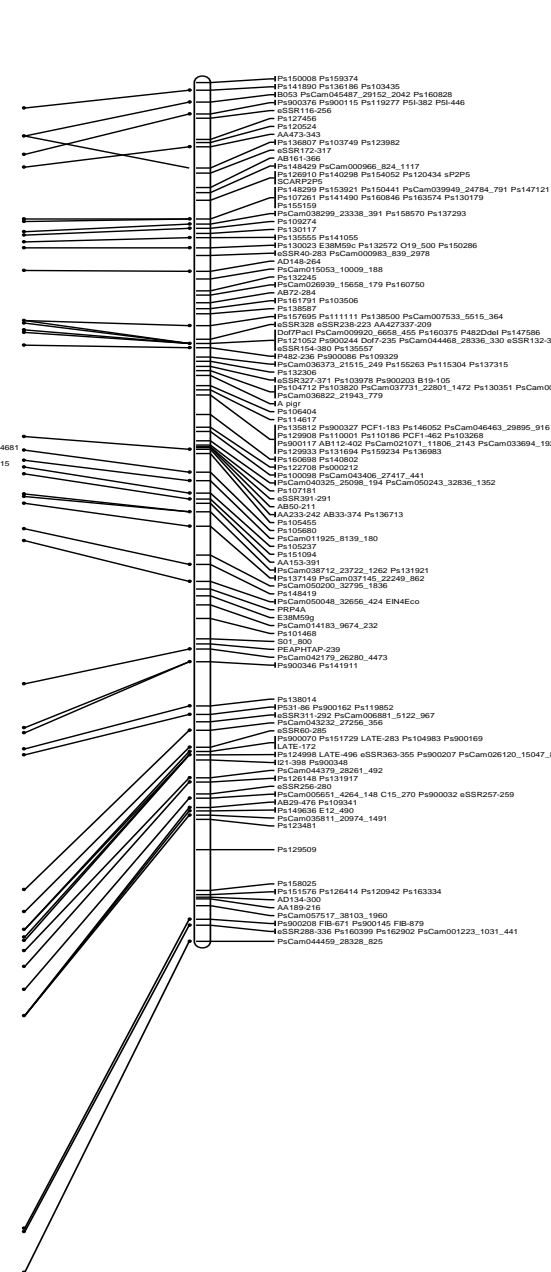

[illegible][illegible][illegible]

**LG4\_JD**

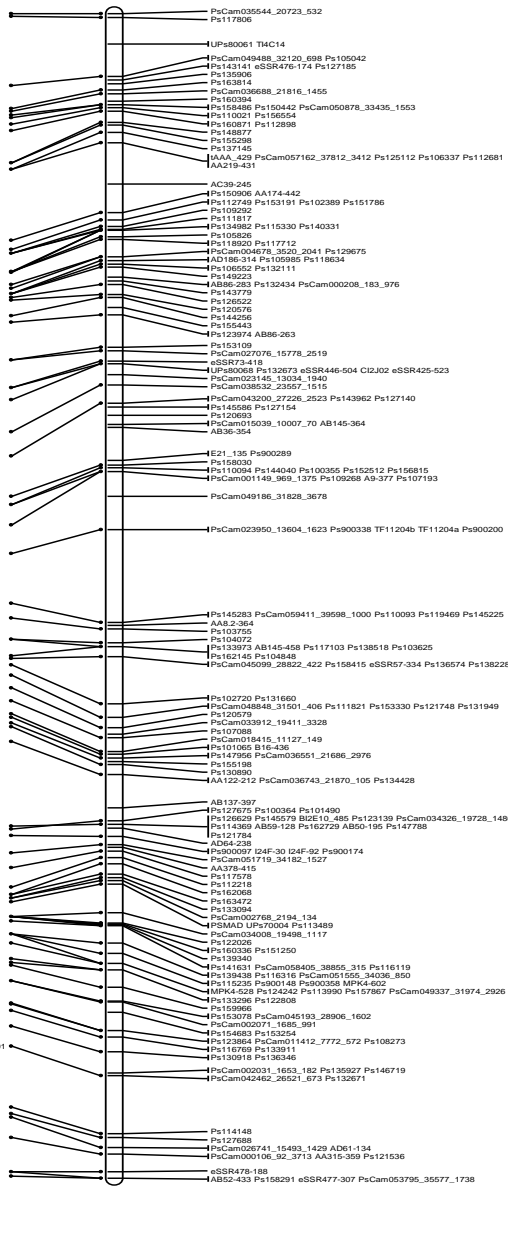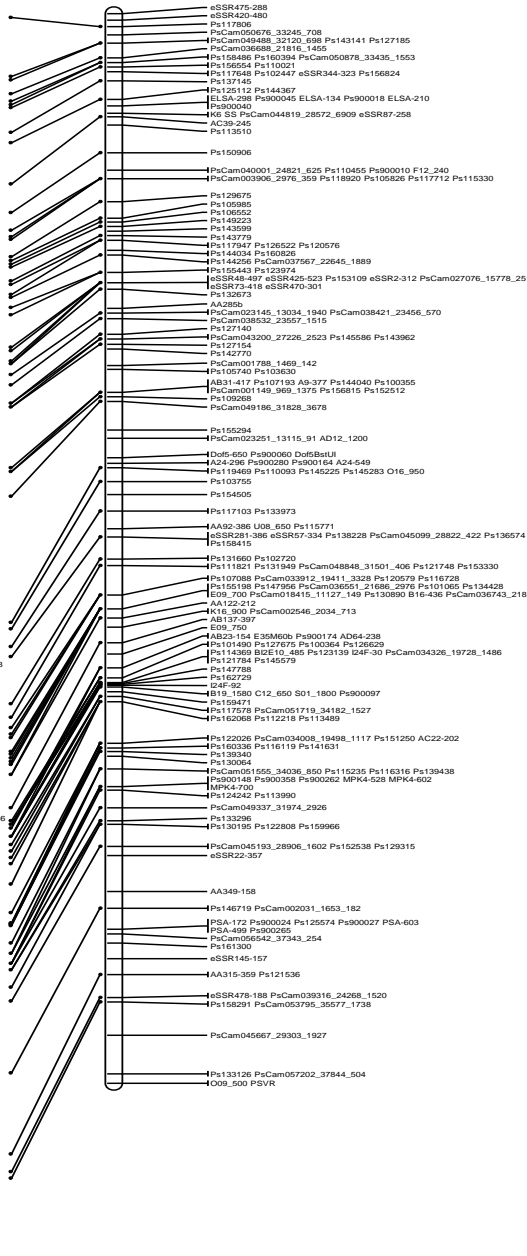

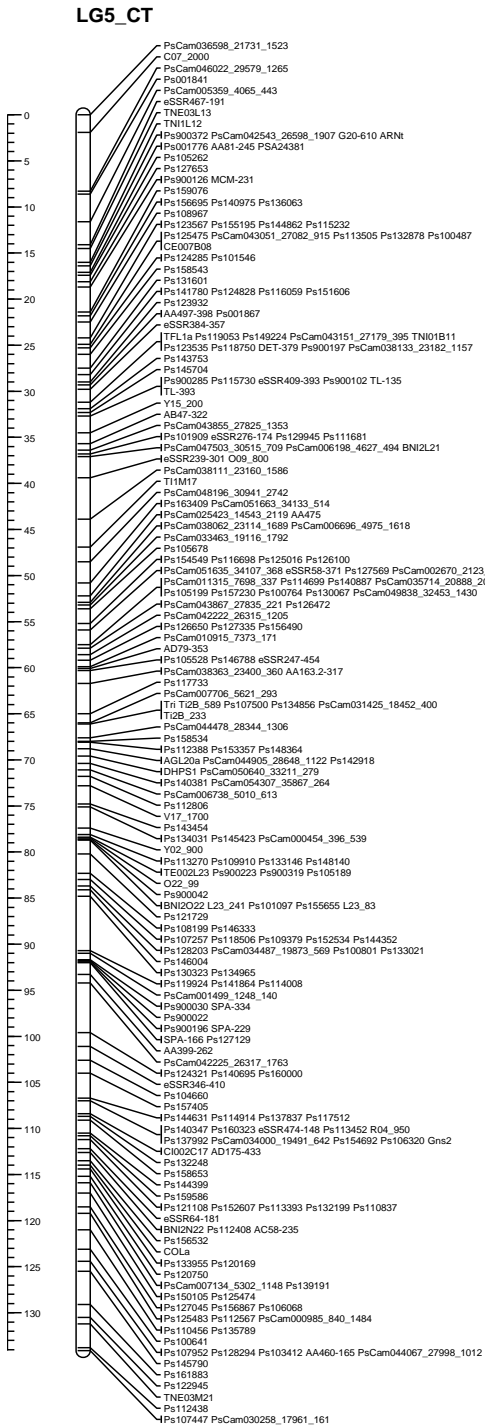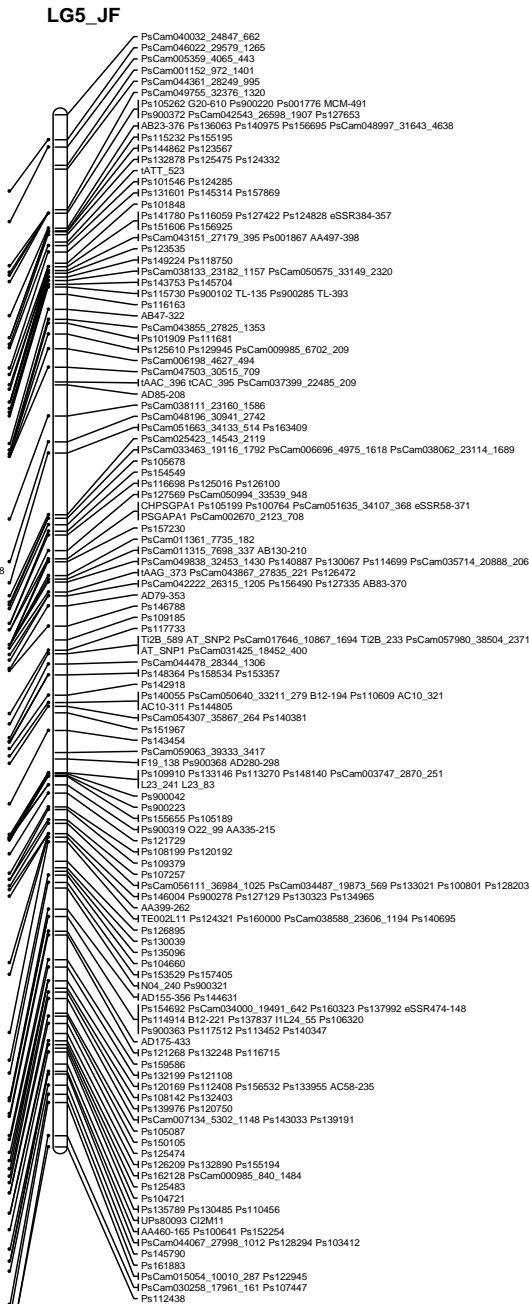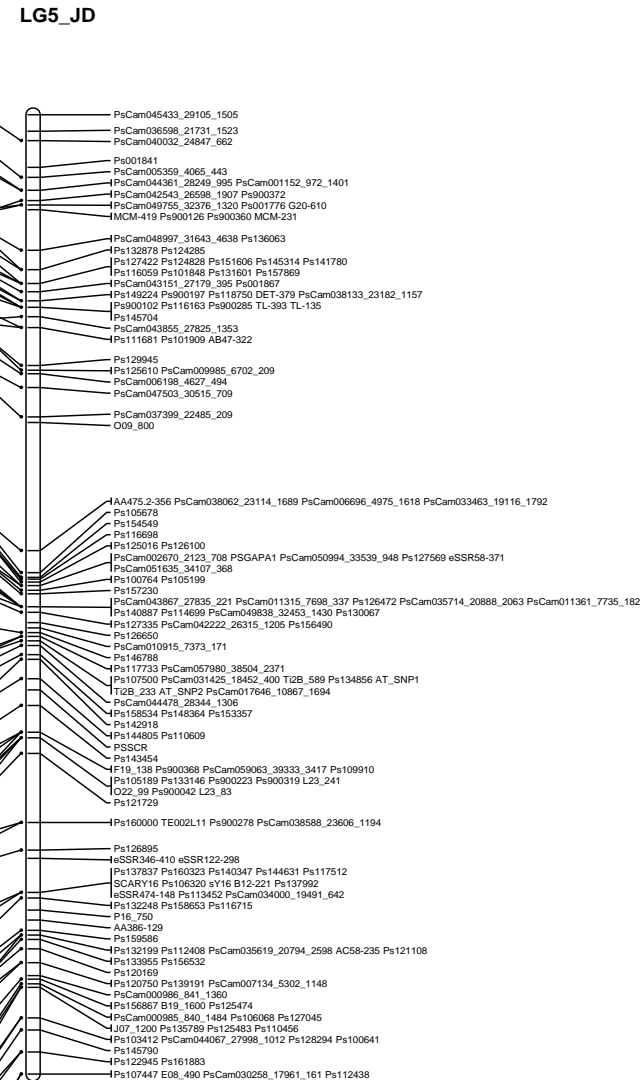

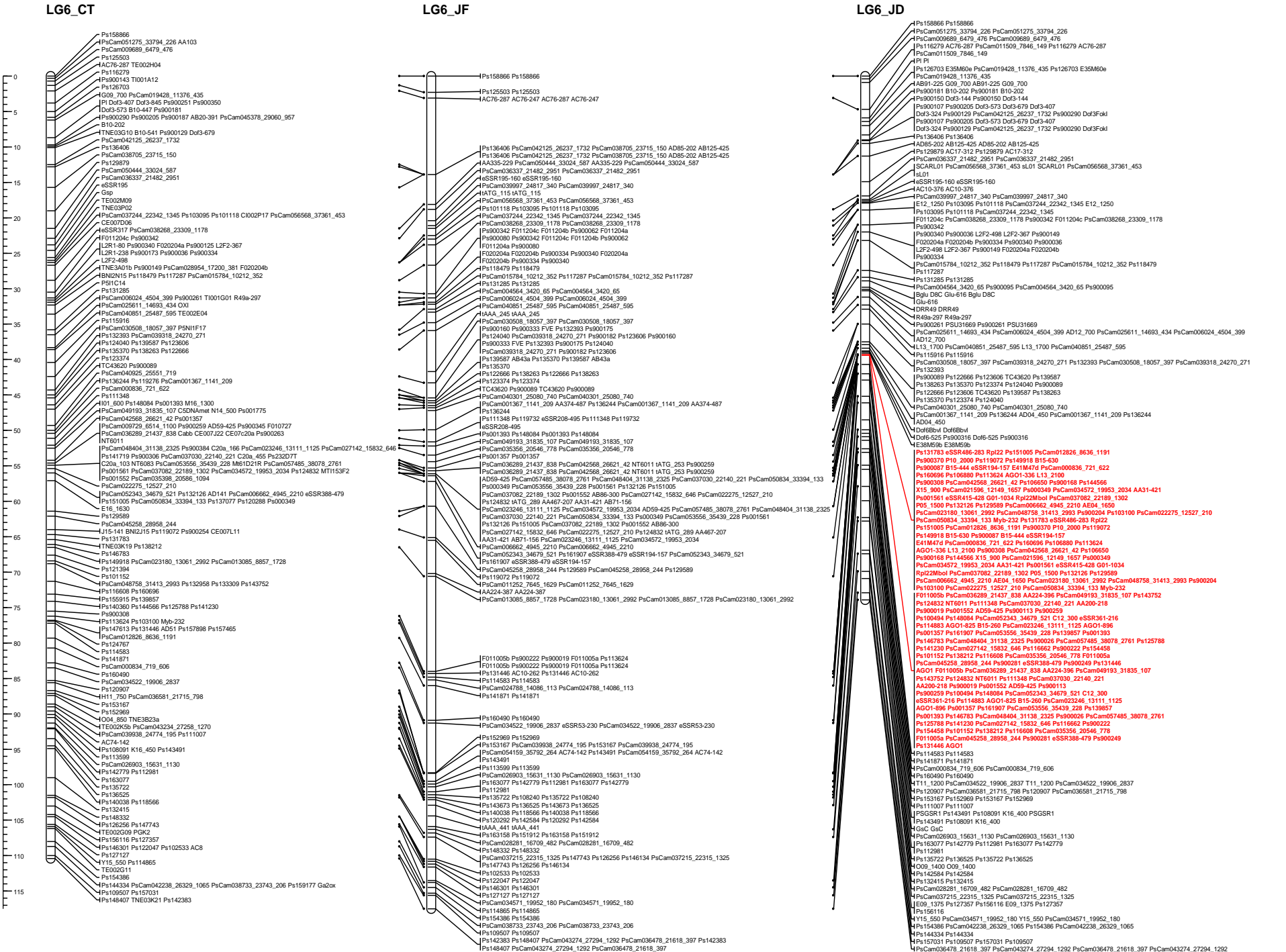

## LG7\_CT

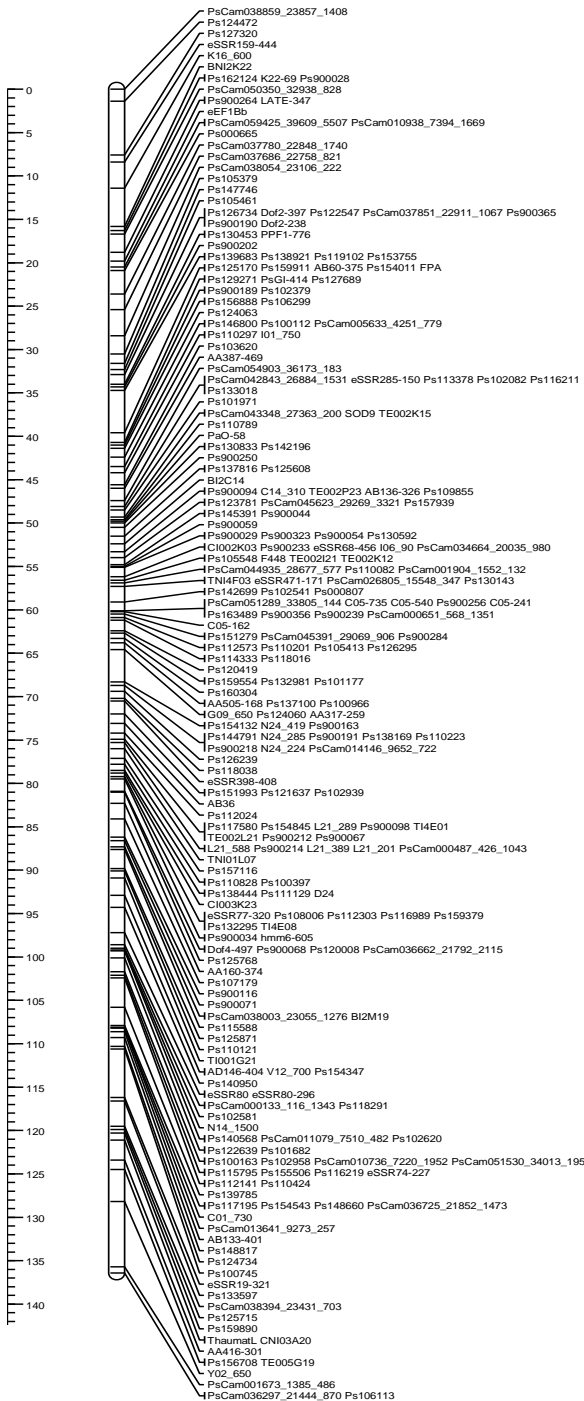

## LG7\_JF

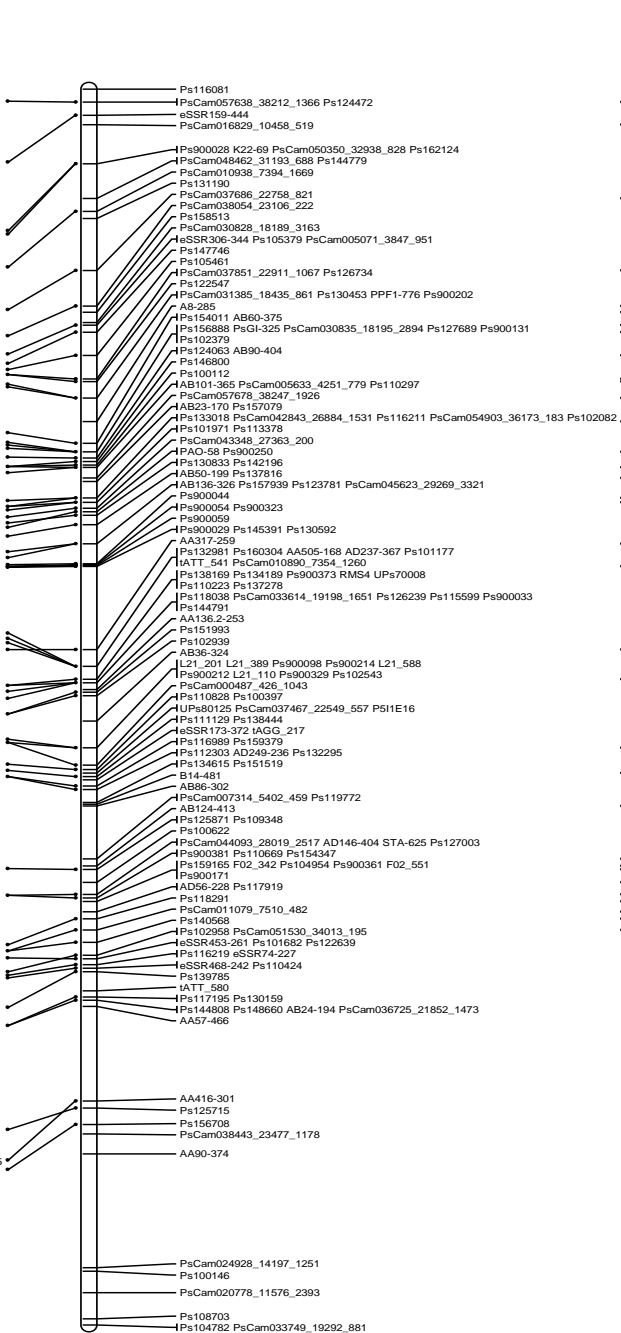

## LG7.1\_JD

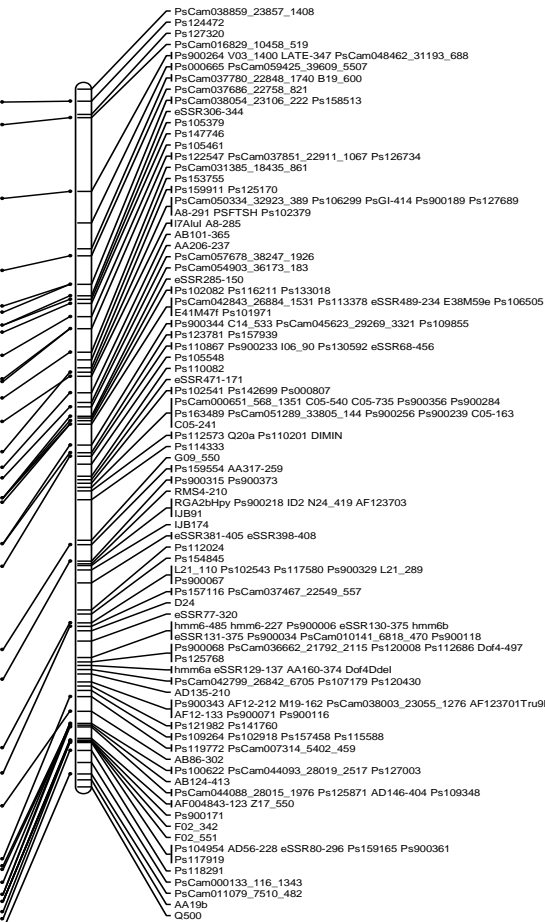

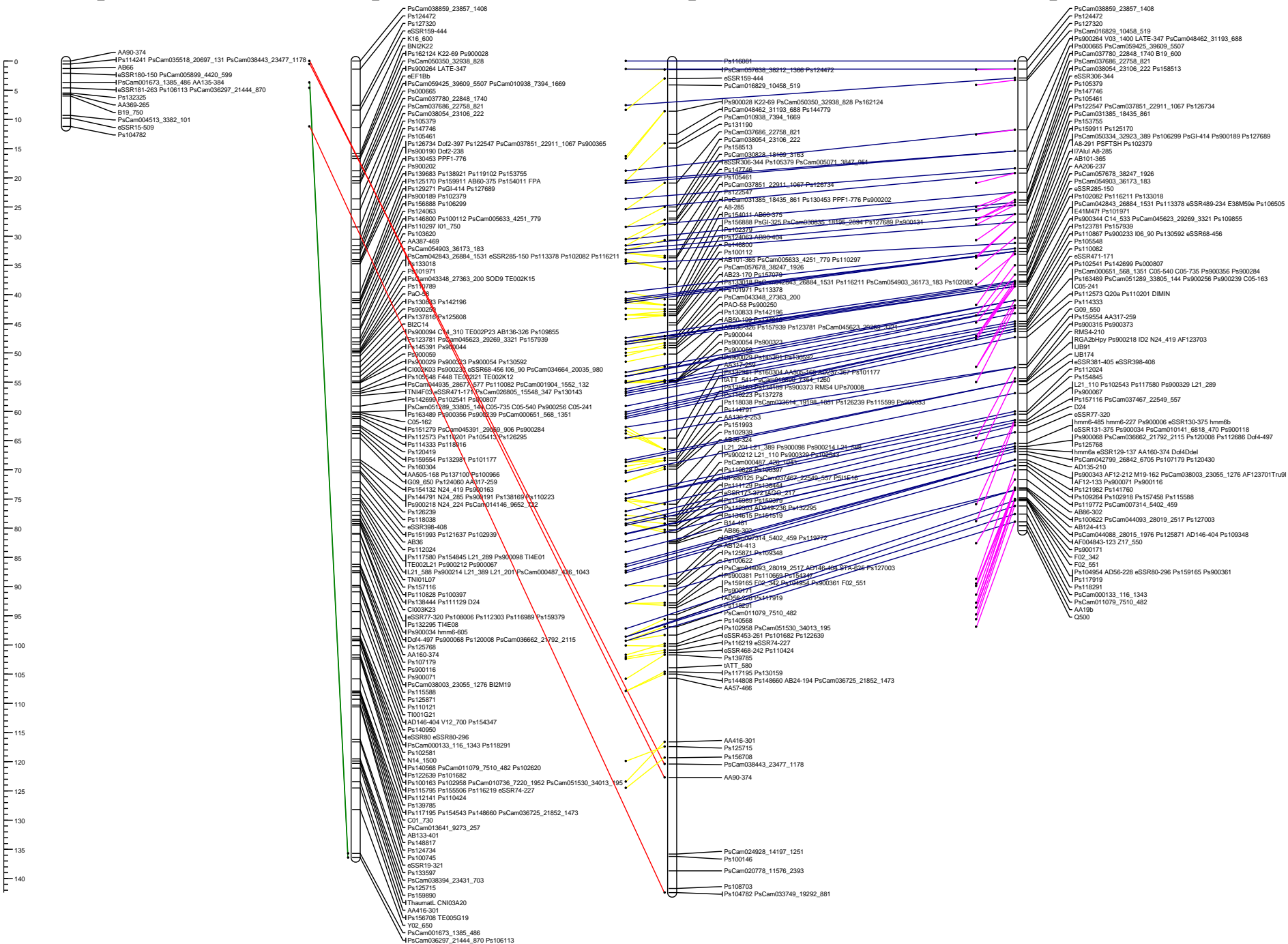

Supplement: Supplementary file 1 [file genes-14-01399-s001.zip › genes-2286623 - Figure S1.pdf]
